# Sulphadoxine-pyrimethamine plus azithromycin for the prevention of low birthweight in Papua New Guinea: a randomised controlled trial

**DOI:** 10.1186/s12916-014-0258-3

**Published:** 2015-01-16

**Authors:** Holger W Unger, Maria Ome-Kaius, Regina A Wangnapi, Alexandra J Umbers, Sarah Hanieh, Connie SN Li Wai Suen, Leanne J Robinson, Anna Rosanas-Urgell, Johanna Wapling, Elvin Lufele, Charles Kongs, Paula Samol, Desmond Sui, Dupain Singirok, Azucena Bardaji, Louis Schofield, Clara Menendez, Inoni Betuela, Peter Siba, Ivo Mueller, Stephen J Rogerson

**Affiliations:** Department of Medicine (Royal Melbourne Hospital), The University of Melbourne, Post Office Royal Melbourne Hospital, Parkville, Victoria 3050 Australia; Papua New Guinea Institute of Medical Research, PO Box 60, Goroka, Eastern Highlands Province 441 Papua New Guinea; Walter and Eliza Hall Institute (WEHI), Parkville, Victoria 3052 Australia; Institute of Tropical Medicine, Nationalestraat 155, 2000 Antwerpen, Belgium; Barcelona Centre for International Health Research (CRESIB), Hospital Clínic-Universitat de Barcelona, Rossello, 132, 7th floor, 08036 Barcelona, Spain; Australian Institute of Tropical Health and Medicine, Faculty of Medicine, Health, and Molecular Sciences, James Cook University, Townsville, Queensland 4811 Australia; Department of Medical Biology, The University of Melbourne, Parkville, Victoria 3010 Australia

**Keywords:** Intermittent preventive treatment, Malaria, Pregnancy, Preterm delivery, Sexually transmitted infections

## Abstract

**Background:**

Intermittent preventive treatment in pregnancy has not been evaluated outside of Africa. Low birthweight (LBW, <2,500 g) is common in Papua New Guinea (PNG) and contributing factors include malaria and reproductive tract infections.

**Methods:**

From November 2009 to February 2013, we conducted a parallel group, randomised controlled trial in pregnant women (≤26 gestational weeks) in PNG. Sulphadoxine-pyrimethamine (1,500/75 mg) plus azithromycin (1 g twice daily for 2 days) (SPAZ) monthly from second trimester (intervention) was compared against sulphadoxine-pyrimethamine and chloroquine (450 to 600 mg, daily for three days) (SPCQ) given once, followed by SPCQ placebo (control). Women were assigned to treatment (1:1) using a randomisation sequence with block sizes of 32. Participants were blinded to assignments. The primary outcome was LBW. Analysis was by intention-to-treat.

**Results:**

Of 2,793 women randomised, 2,021 (72.4%) were included in the primary outcome analysis (SPCQ: 1,008; SPAZ: 1,013). The prevalence of LBW was 15.1% (305/2,021). SPAZ reduced LBW (risk ratio [RR]: 0.74, 95% CI: 0.60–0.91, *P* = 0.005; absolute risk reduction (ARR): 4.5%, 95% CI: 1.4–7.6; number needed to treat: 22), and preterm delivery (0.62, 95% CI: 0.43–0.89, *P* = 0.010), and increased mean birthweight (41.9 g, 95% CI: 0.2–83.6, *P* = 0.049). SPAZ reduced maternal parasitaemia (RR: 0.57, 95% CI: 0.35–0.95, *P* = 0.029) and active placental malaria (0.68, 95% CI: 0.47–0.98, *P* = 0.037), and reduced carriage of gonorrhoea (0.66, 95% CI: 0.44–0.99, *P* = 0.041) at second visit. There were no treatment-related serious adverse events (SAEs), and the number of SAEs (intervention 13.1% [181/1,378], control 12.7% [174/1,374], *P* = 0.712) and AEs (intervention 10.5% [144/1,378], control 10.8% [149/1,374], *P* = 0.737) was similar. A major limitation of the study was the high loss to follow-up for birthweight.

**Conclusions:**

SPAZ was efficacious and safe in reducing LBW, possibly acting through multiple mechanisms including the effect on malaria and on sexually transmitted infections. The efficacy of SPAZ in the presence of resistant parasites and the contribution of AZ to bacterial antibiotic resistance require further study. The ability of SPAZ to improve pregnancy outcomes warrants further evaluation.

**Trial registration:**

ClinicalTrials.gov NCT01136850 (06 April 2010).

**Electronic supplementary material:**

The online version of this article (doi:10.1186/s12916-014-0258-3) contains supplementary material, which is available to authorized users.

## Background

Infants born with low birthweight (LBW, <2,500 g) due to pre-term delivery (PTD, <37 weeks) and/or fetal growth restriction are at increased risk of morbidity and mortality [1,2]. Malaria in pregnancy is an important cause of fetal growth restriction, PTD, and adverse birth outcomes (miscarriage, stillbirth) [3], primarily due to inflammatory processes secondary to sequestration of *Plasmodium falciparum* (*P.f.*)*-*infected red blood cells in the placenta [4]. Many women with placental malaria are asymptomatic and such an infection can be undetectable by peripheral blood examination [2]. In areas of moderate-to-high endemicity, primigravidae are most at risk [5]. The burden of malaria and LBW is highest in low-income countries and, worldwide, 125.2 million pregnant women were at risk of infection in 2007 [6].

Intermittent preventive treatment of malaria in pregnancy (IPTp), namely the periodic administration of a curative dose of an antimalarial, provides intermittent chemoprophylaxis and clears asymptomatic infections. The World Health Organisation recommends monthly courses of sulphadoxine-pyrimethamine (SP) from second trimester in areas of Africa with moderate-to-high malaria transmission [7]. Although IPTp-SP remains generally efficacious, increasing drug resistance has generated a need for new IPTp candidates [8,9]. Azithromycin (AZ) is an azalide antibiotic with favourable antimalarial properties and a good safety profile in all trimesters of pregnancy [10]. AZ combined with SP (SPAZ) reduced PTD and malaria at delivery in one study in Malawi [11,12]. Further, AZ has the potential to simultaneously clear sexually transmitted infections (STIs) and reproductive tract infections that are known to increase the risk of PTD and adverse pregnancy outcomes [13]. The antimalarial effect of AZ has been associated with blood levels at 96 h, and doses of 4 to 4.5 g over 2 to 3 days appear to be required to obtain appropriate levels [10,14,15].

To date, IPTp has been studied only in Africa, where *P.f.* predominates, and IPTp use outside of Africa is currently not endorsed by the World Health Organisation [7]. However, many pregnant women elsewhere are at risk of malaria in areas, such as Papua New Guinea (PNG), where both *P.f.* and *P. vivax* (*P.v.*) are endemic [3,16]. *P.v.* also causes adverse pregnancy outcomes, through less well understood mechanisms [17].

We evaluated the efficacy and safety of IPTp with SPAZ to prevent LBW and to reduce the prevalence of malaria and anaemia at delivery in PNG.

## Methods

### Ethics

Ethical approval for the study protocol (Additional file 1) was obtained from the PNG Institute of Medical Research (PNGIMR) Institutional Review Board, the PNG Medical Research Advisory Council, and the Melbourne Health Human Research Ethics Committee. The trial was registered with the United States National Institutes of Health Clinical Trials Registry (NCT01136850, registered 06 April 2010) and has been reported according to CONSORT guidelines (Additional file 2). Because there is currently insufficient evidence to support a general recommendation for the use of IPTp-SP outside Africa [7], and because SP alone is often ineffective against *P.v.* [18], which causes around 40% of malaria infections in PNG, we compared SPAZ-IPTp to a single course of SP and chloroquine (CQ) to eliminate infection. The study was conducted in accordance with Good Clinical Practice guidelines (ICH GCP E6). External monitoring was provided by one independent monitor and the Data Safety Monitoring Board (DSMB). All participants provided informed written consent. The legal age of consent for women in PNG is 16 years. The trial was registered in April 2010, after 336 women had joined the study, as a result of miscommunication between the principal investigator and the on-site clinical team.

### Interventions

The trial had two treatment arms with a 1:1 allocation ratio. In the intervention arm, women received three courses of SP (3 tablets [500/25 mg] given once, Micro Labs Ltd., India) and AZ (2 tablets [500 mg] twice daily for 2 days, Pfizer, USA), at minimum intervals of 4 weeks. Women assigned to the control arm received one course of SP (3 tablets, 500/25 mg) and CQ (3 or 4 tablets [150 mg], daily for 3 days, Medopharm, India) at enrolment, followed by monthly courses of placebo equivalent (J. Bonal S.A., Spain). Women who did not already own an insecticide-treated net were given one at enrolment if available; local stock-outs meant that 8% of women did not own, or receive, bed nets.

Intake of SP and the first of four doses of AZ (1 g; intervention arm), and intake of SP (or placebo equivalent) and the first of three doses of CQ (or placebo equivalent; control arm) was supervised at an antenatal clinic. Drugs were administered with dry biscuits, and women were observed for a minimum of 30 minutes following ingestion of study medications. Treatment was rescheduled for first trimester pregnancies and women with a positive malaria rapid diagnostic test. Adherence to the remaining three doses of AZ or two doses of CQ (or CQ placebo equivalent) for first and second courses was assessed retrospectively at second and third study visits, respectively, through help of a questionnaire. Drug levels were not taken.

### Objectives and outcome measures

The primary objective was to compare efficacy of IPTp with SPAZ with a single treatment course of SPCQ to prevent LBW.

The primary outcome measure was the proportion of live born, singleton infants without congenital malformations with LBW. Secondary outcome measures included mean birthweight, the proportion of women with malaria and anaemia at delivery, and the proportion of women who delivered a preterm infant. Safety outcomes included the number of adverse events (AEs) and, specifically, the number of maternal deaths, stillbirths, neonatal deaths, and infants with congenital abnormalities.

### Trial design, setting, and participants

We conducted a single-blinded block-randomised controlled trial.

The control arm was adapted from the policy for prevention of malaria in pregnancy in PNG when the trial was designed, which involved clearance of infection with a single dose of SP plus CQ for 3 days at first antenatal visit, followed by weekly doses of CQ until delivery (PNG standard of care) [16]. Because *P.f.* is highly resistant to CQ in PNG [19,20], and compliance was poor, we instead endeavoured to provide all participants with insecticide-treated nets. To allow for participant blinding, women assigned to control treatment were provided placebo doses of SPCQ at subsequent study visits. A previous survey of molecular markers of SP resistance in children from the study area demonstrated absence of ‘high’ and ‘super’ resistant *P.f.* and a low prevalence of the *dhps* K540E mutation (20%) [18,21]. The dose and regimen for AZ was selected based on previous pharmacokinetic and tolerability studies [10], and to give adequate drug levels at 96 hours to clear *P.f.* and *P.v*. [14,22].

Pregnant women were enrolled between 23 November 2009 and 15 August 2012 at nine antenatal clinics in the Madang and Sumkar districts of Madang Province, PNG (Additional file 3). Pregnancy outcome follow-up was concluded on 28 February 2013. Participating health facilities with labour wards included Modilon General Hospital, Yagaum Hospital, and the health centres in Alexishafen and Mugil.

In a 2006 survey at one of the participating health centres, prevalence at first antenatal visit of *P.f.* and *P.v.* was 30.3% and 8.1% (by light microscopy), respectively, and the prevalence of LBW amongst women not using insecticide-treated nets was 17% (unpublished data). The study area experiences year-round malaria transmission and was considered hyper-endemic at the time the trial was designed [23]. *Chlamydia trachomatis*, *Neisseria gonorrhoeae*, and syphilis are thought to be common in pregnant women in PNG [24]. Antenatal HIV prevalence at the provincial hospital (Modilon General Hospital) was 1.1% (2009 – 2012, unpublished audit data).

### Screening, enrolment, and follow-up procedures

Community campaigns were held to raise awareness of the trial. All pregnant women presenting for their first antenatal visit at participating health centres were invited to attend group information sessions and were screened. Interested women were excluded if they had i) gestation >26 weeks by abdominal palpation, ii) haemoglobin <6 g/dL and symptomatic as a result of anaemia, iii) previous serious adverse reaction to study medications, iv) permanent disability and chronic medical conditions, v) known multiple pregnancy, vi) unavailable for follow-up, or vii) age <16 years. We collected detailed information on reasons for non-inclusion at screening for a subset of antenatal recruitment clinic sessions (n = 279), during which 30.8% (860/2,793) of all women randomised to treatment were enrolled. Due to logistic constraints, we were unable to gather demographic and clinical background data on women who were screened but not enrolled. Written informed consent was obtained, a focussed clinical examination performed, and socio-demographic and clinical data collected using standardised case report forms. A venous blood sample was taken and peripheral blood smears were prepared. Women with malaria symptoms and/or haemoglobin <9 g/dL (HemoCue Ltd, Angelholm, Sweden; accuracy of 0.1 g/dL) were tested using a malaria rapid diagnostic test (CareStart™ P.f/Pan combo, AccessBio, USA). Anaemia and malaria were treated with iron/folate supplements and albendazole, and quinine (in first trimester, 300 mg, 2 tablets orally 3 times daily for 7 days) or artemether-lumefantrine (in second and third trimesters, 20/120 mg, 4 tablets 6 times over 3 days), as per national protocol [25]. Women treated for malaria had their study medication administration rescheduled 2 weeks later. Women were screened for syphilis (Syphicheck-WB, QualPro, India) and treated with 2.4 MU of benzathine penicillin G if found positive.

We were able to offer a sub-set of participants an obstetric ultrasound (Logiqbook XP, General Electric Medical Systems, UK) within a week of enrolment; technical problems with our ultrasound machine precluded scanning for the entire trial period. Fetal biometry alone was used to estimate gestational age (GA), as the majority of women were unable to report their last menstrual period and/or menstrual cycle characteristics, and Ballard scores correlated poorly with GA in our cohort. For women presenting early, GA was estimated as per British Medical Ultrasound Guidelines [26]. Women who had their first scan after mid-second trimester had their GA estimated according to Hadlock et al. [27]. The earliest scan available for each woman was selected to estimate GA at delivery. At subsequent scheduled study visits, peripheral blood smears and samples were collected, and routine antenatal examination performed.

At the second treatment visit, a self-collected vaginal swab was obtained for testing for *C. trachomatis*, *N. gonorrhoeae*, and *Trichomonas vaginalis* for a subset of women. Once available, participants were notified of results and referred for treatment.

Participants were followed-up until delivery, and birthweights measured by study nurses to the nearest 10 g using digital infant scales (Cupid 1, Charder Medical, Taiwan). Time elapsed between birth and birthweight measurements was documented, and newborns were checked for congenital abnormalities. Deliveries <22 gestational weeks were categorised as miscarriages. Maternal haemoglobin was measured, peripheral blood placental impression and cord blood smears prepared, and placental biopsies were taken. Women were invited to re-attend at 4 to 6 weeks postpartum with their baby. A team of community liaison officers, reporters, and nurses was dedicated to the follow-up of women who did not present for delivery at a participating health centre within one month of the estimated delivery date in order to establish pregnancy outcome.

### Adverse events monitoring and reporting

Case report forms were completed for maternal and neonatal AEs detected at scheduled antenatal and postpartum visits, at delivery, and during non-scheduled visits. The study clinician on-call was alerted by the nursing team upon detection of a possible serious adverse event (SAE), whereby cases were clinically evaluated and reported shortly thereafter, but allowing for a maximum time frame of 24 hours for reporting of cases detected at distal study sites. AEs were considered SAEs if fulfilling one of the following criteria: the event resulted in death, was a congenital abnormality, resulted in hospitalisation or prolongation of existing hospitalisation, was life-threatening, resulted in persistent/significant disability, or was deemed serious for other medically significant reasons by the study physicians. SAE reports were completed for mothers who experienced miscarriages or stillbirths. Analysis of all AEs was by actual drugs received (hence adjusted for crossover) and included all women who received trial medications (n = 2,752). Drug-related AEs represented drug side effects. Reports of side effects at each treatment course (including placebo administration in the control arm) were considered as separate AEs.

A detailed report of each SAE was sent immediately to the DSMB, and the Malaria in Pregnancy Consortium and Pfizer drug safety groups. Assessments of the relationship between AEs and study medication were undertaken by the investigators and reports forwarded to the DSMB and an independent clinician for scrutinisation.

### Laboratory evaluations

Labelled blood and impression smears were air-dried and stained with 4% Giemsa for 30 minutes. Thick smears were used to count the number of asexual parasites per 200 leukocytes (or per 500 if <10 parasites/200 leukocytes), assuming 8,000 leukocytes/μL of blood; slides were declared negative if no parasite was seen in 200 oil-immersion fields. Two microscopists read each slide, and third reads were performed to resolve discrepant results. When species discrepancies remained after third reads, qPCR was performed on maternal venous blood, and these results were considered definitive [28]. A sample of 10 mL of venous blood was collected from each participant at both enrolment and delivery into Lithium Heparin vacutainers (BD, USA), and plasma was separated and stored at −80°C until further analyses. Light microscopy and qPCR were undertaken at the PNGIMR. Vaginal swabs were stored, extracted, and analysed by qPCR for presence of beta globin (positive control), *C. trachomatis*, *N. gonorrhoeae*, and *T. vaginalis* as described elsewhere [29].

A placental biopsy (2.5 × 2.5 × 1 cm) was collected and included the thickness of the placenta from the maternal to the fetal side without reaching the fetal membrane. Biopsies were stored at room temperature in 10% neutral buffered formalin and trimmed to fit histology cassettes. Cassettes were transported to Melbourne, Australia, where they were wax embedded. Histological sections were stained with Giemsa and coverslipped. Slides were returned to the PNGIMR for analysis. A subset of 423 placental biopsies were read at CRESIB, Barcelona, Spain, by Prof J Ordi, who also provided quality control on slide reading. Placental malaria was staged according to the presence/absence of three histological features: infected erythrocytes, hemozoin in monocytes/macrophages, and hemozoin in fibrin deposits [30,31]. Placental malaria was classified as active (parasites detected) or past infection (malaria pigment without parasites) [30,31].

### Randomisation and masking

Following enrolment, women were randomly allocated to SPCQ/placebo or SPAZ using a randomisation list prepared by an independent statistician in Stata 10.0 (StataCorp, USA). Each treatment (SPCQ/placebo or SPAZ) was randomly assigned to four different treatment codes, resulting in a total of eight treatment codes (letters A–H). A block randomisation procedure was subsequently used, with blocks of 32, each containing four women assigned to each treatment code. Study drugs were packaged and labelled at the PNGIMR by staff not involved in the trial, and allocation codes were kept in a locked file cabinet offsite. Women were randomised to treatment codes using pre-made, opaque, consecutively numbered envelopes. The treatment code was revealed after completion of enrolment and immediately prior to treatment. Taste and colour of placebo and active medication differed at times; enrolled women were not told of their allocation, but it was impossible to blind clinical staff directly involved with drug administration. All other personnel (laboratory and administrative staff, data entry clerks) were blinded to the assignments. The allocation code was broken at completion of laboratory analyses and upon finalisation of data collection, entry, and cleaning. The statistical analysis was performed after the database was locked, but was not blind to treatment allocation.

### Sample size calculations

The sample size calculation was based on the assumption that SPAZ lowers the proportion of LBW babies by 30% compared to a single course of SP and CQ when given in conjunction with insecticide-treated nets (12.0% to 8.4%). Taking into consideration 20% loss to follow-up, a power of 80%, and 95% confidence, 1,396 women per arm were needed to demonstrate efficacy (Additional file 1).

### Statistical analysis

Data was double-entered into case report form-specific databases (FoxPro 9.0, Microsoft, USA). Individual databases were merged and data analysed using Stata 12.0 (StataCorp, USA). Both intention-to-treat (ITT) and per-protocol (PP) analyses were carried out for the primary outcome (LBW), birthweight, malaria, and anaemia at delivery. ITT analysis included all women randomised to treatment, except those that were retrospectively excluded because of incomplete consent forms. Women who experienced unintentional crossover were assessed by original assigned groups. PP analysis only included women who received two or three treatments without crossover. Safety analyses were performed for all women who received treatment, including those with incomplete consent forms, and were done according to actual treatment received at enrolment (adjusted for crossover). Women with incomplete consent forms (lack of appropriate signature, date, and/or witness if illiterate) had been screened, had received counselling, had provided written informed consent, and were randomised to treatment. As per the trial monitor’s recommendations, these women were retrospectively excluded from all trial analyses bar those pertaining to drug safety.

Only birthweights from singleton pregnancies of live births ≥22 weeks’ gestation with no congenital abnormality and measured within 7 days of delivery were included in birthweight analyses [32].

To assess the distribution characteristics of continuous variables, data were graphed as a kernel density plot including a normal density and the Shapiro-Wilk test was performed. Linearity of continuous data was assessed through visual inspection of scatter plots. Univariate comparisons of variables were subsequently performed using the χ^2^ test or Fisher’s exact test for categorical data, the Student’s *t*-test or ANOVA for parametric data, and Mann Whitney-U or Kruskal-Wallis tests for nonparametric data. Unadjusted risk ratios (RR) were calculated for primary and secondary categorical outcomes including LBW, and difference in means were calculated for continuous parametric data including birthweight and haemoglobin. *P* <0.05 was considered significant.

All factors with a tendency for association with LBW when analysed univariately (defined as *P* <0.1) were included in a multivariable Poisson regression model with robust error variance as a starting model for a backward stepwise elimination model selection procedure [33]. A multivariable linear regression was used to calculate an adjusted difference in mean birthweight. Multivariate analyses for LBW/birthweight were performed in order to validate the effect of drug treatment on the primary outcome observed in crude analysis. For women with known pregnancy outcome but missing birthweight, multiple imputation was performed to derive estimated birthweights by drawing 20 imputed data sets from a Bayesian posterior predictive distribution of the missing data [34].

*A priori* tests for interaction (defined as *P* <0.15 of interaction term) between the intervention and gravidity (categorised as primigravida, multigravida), bed net use prior to enrolment (categorised as non-user, and user of a bed net [untreated and treated combined]), maternal height (categorised as low [<150 cm] and normal height [≥150 cm]) and maternal ethnic grouping (categorised as maternal highlander parentage and non-highlander parentage) were performed on the final multivariable model for LBW using the Wald test. Stratified analyses for the primary outcome using the same variables were undertaken.

Lastly, we estimated the population-attributable fraction of LBW due to malaria using established methodology [1]. This calculation was based on the assumption that LBW can only be attributed to malaria if there was evidence of placental malaria (past or active infection).

### Role of funding source and ethical approval

This trial was supported by the Malaria in Pregnancy Consortium (funded by the Bill & Melinda Gates Foundation, 46099), and the Pregvax Consortium (European Union’s Seventh Framework Programme FP7-2007-HEALTH, PREGVAX 201588, and the Spanish Government EUROSALUD 208 Programme). Azithromycin was provided by Pfizer Inc. as part of an Investigator-Initiated Research grant (WS394663). Funding sources did not have any involvement in study design, collection, analysis, and interpretation of data, and compilation and submission of this report.

## Results

**Figure 1:**
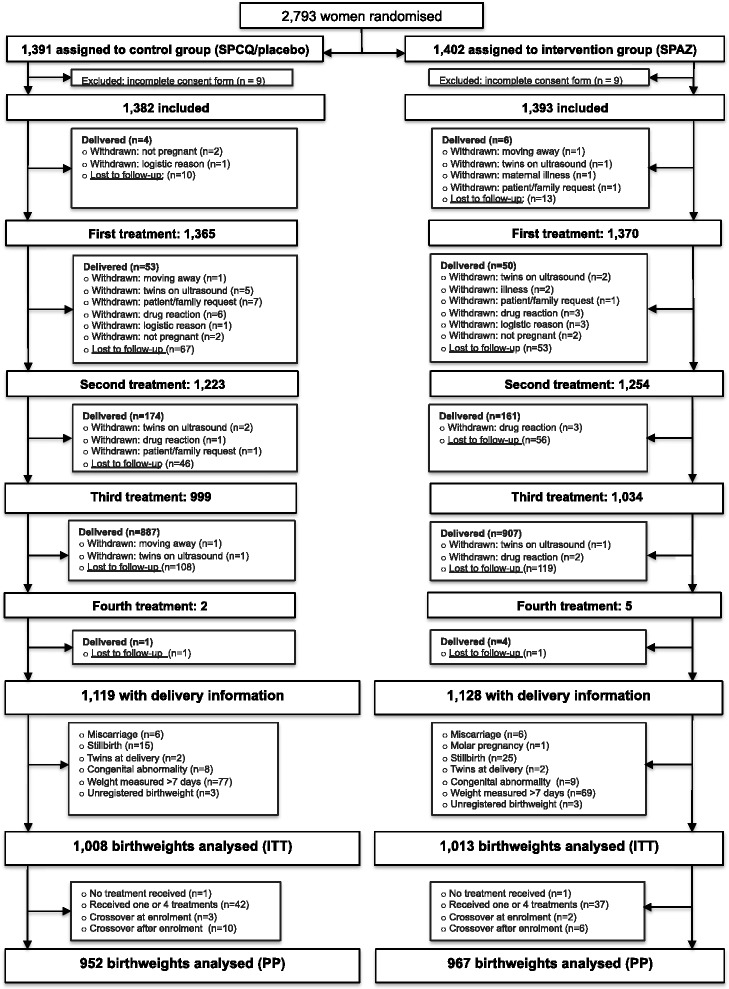
**Trial profile.** IPTp, Intermittent preventive treatment in pregnancy; SP, Sulphadoxine-pyrimethamine; CQ, Chloroquine; AZ, Azithromycin; ITT, Intention-to-treat analysis; PP, Per protocol analysis.

**Figure 2:**
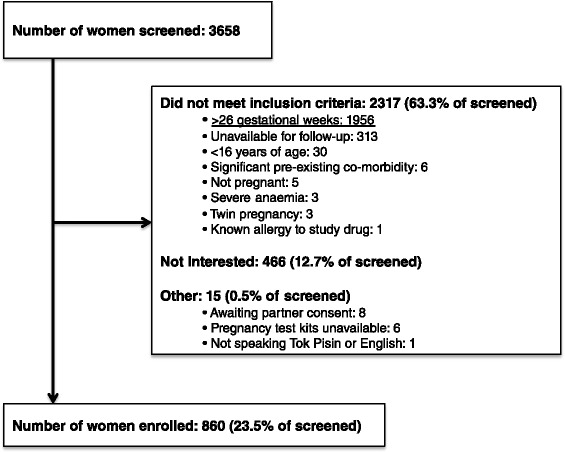
**Detailed screening data for 279 clinic sessions (held at nine antenatal clinics) during which 860 of 2,793 trial participants (30.8%** **) were recruited.**

Of women screened at antenatal clinics, 2,793 were enrolled between November 2009 and August 2012. The trial flowchart is outlined in Figure 1: 18 women were excluded due to incomplete consent forms, leaving 2,775 women (99.4%) in the ITT cohort at baseline. Their demographic and clinical characteristics were similar across treatment arms and are presented in Table 1. Overall, 50.2% (1,390/2,770) of participants were primigravida, 62.2% (1,720/2,764) resided in rural areas, 81.4% (2,157/2,650) were anaemic (Hb <11 g/dL), and 7.4% (204/2,766) had microscopy-detected malaria parasitaemia. The principal reason for exclusion at screening was advanced GA (Figure 2).

**Table 1:** **Baseline characteristics of study participants**

**Characteristic**	**Control (SPCQ and placebo)**		**Intervention (SPAZ)**	
	**(n = 1,382)**		**(n = 1,393)**	
Age, years	24.5	[5.4]	24.4	[5.5]
Height, cm; n = 2,729	154.3	[6.0]	154.1	[5.8]
Body mass index, kg/m^2^; n = 2,721	22.8	[2.9]	22.8	[2.9]
Mid-upper arm circumference, cm; n = 2,714	23.9	[2.5]	24.0	[2.6]
Fundal height, cm; n = 2,771	21.1	[4.2]	21.0	[4.3]
Haemoglobin (Hb), g/dL; n = 2,650	9.7	[1.5]	9.7	[1.5]
Anaemia, Hb <11 g/dL	1,058/1,313	(80.6)	1,099/1,337	(82.2)
Syphilis	15/1,122	(1.3)	20/1,166	(1.7)
**No. of previous pregnancies**				
0	681	(49.4)	709	(51.0)
1	292	(21.2)	279	(20.1)
≥2	406	(29.4)	403	(29.0)
Previous adverse pregnancy outcome	103/1,375	(7.5)	119/1,389	(8.6)
**Used bed net before enrolment (2 wks)**				
Not used	322	(23.4)	347	(25.0)
Used, without insecticide	588	(42.7)	561	(40.4)
Used, insecticide treated	466	(33.9)	481	(34.6)
Given new bed net at enrolment	873/1,375	(63.5)	849/1,385	(61.3)
Used antimalarials in this pregnancy	146/1,340	(10.9)	161/1,366	(11.8)
**Peripheral parasitaemia by microscopy**				
Any species^a^	100/1,376	(7.3)	104/1,390	(7.5)
*Plasmodium falciparum*	87	(6.3)	90	(6.5)
*Plasmodium vivax*	11	(0.8)	12	(0.9)
Smoker	249/1,380	(18.0)	275/1,392	(19.8)
Rural residence	844/1,375	(61.4)	876/1,389	(63.1)
Literate	1,223/1,376	(88.9)	1,248/1,391	(89.7)
Income-generating activity (woman)	728/1,304	(55.8)	729/1,326	(55.0)
Income-generating activity (partner)	939/1,358	(69.2)	947/1,378	(68.7)
Highlander parentage	99/1,380	(7.2)	78/1,393	(5.6)

Data are mean [standard deviation], or number (%).

^a^Includes two *P. malariae* in the control, and one *P. malariae*, one *P. ovale*, and one mixed *P. falciparum* and *P. ovale* infections in the intervention group.

Of women in the ITT cohort 81.0% (2,247/2,775) had birth outcome information collected (follow-up completed in February 2013), and 72.8% (2,021/2,775) had birthweights of congenitally normal live singletons measured within 7 days of delivery and were therefore eligible for inclusion in the primary outcome analysis. For 1,013 women who were randomised to SPAZ and included in the ITT birthweight analysis, the mean number of doses of SPAZ was 2.79 ± 0.5 (median 3, range 0–4). Women who were excluded from the ITT birthweight analysis (n = 754) were more commonly malaria infected at baseline, had lower literacy, more commonly resided in rural areas, and had lower fundal heights compared to those included (Additional file 4), but had similar baseline characteristics when assessed according to treatment arm (Additional file 4). There was no significant difference in the proportion of women excluded from ITT birthweight analyses by trial arm (SPCQ 27.1% [374/1,382] vs. SPAZ 27.3% [380/1,393], *P* = 0.898; Figure 1; Additional file 4). Amongst exclusions, 62.9% (474/754) of women were lost for pregnancy outcome follow-up: their proportion did not differ between treatment arms (SPCQ 16.8% [232/1,382] vs. SPAZ 17.4% [242/1,393], *P* = 0.682).

The overall prevalence of LBW was 15.1% (305/2,021) and the mean birthweight was 2,943 ± 479 g. Amongst women who had pregnancy dating by ultrasound, 8.6% (113/1,320) delivered preterm. Overall, 3.1% (63/2,045), 18.8% (278/1,472), and 7.4% (109/1,472) of women had peripheral parasitaemia, placenta malaria (past and active), and active placental malaria, respectively, and 74.4% (1,389/1,868) were anaemic (Hb <11 g/dL) at delivery.

Compared to controls, women who received SPAZ had a lower risk of LBW (RR: 0.74, 95% CI: 0.60–0.91, *P* = 0.005), translating into an absolute risk reduction (ARR) of 4.5% (95% CI: 1.4–7.6) and a number needed to treat of 22 (Table 2). Using imputed birthweights for 152 women with pregnancy outcome but missing birthweight, the RR for LBW with SPAZ was 0.74 (95% CI: 0.60–0.92, *P* = 0.007). When adjusted for factors associated with LBW on univariate analysis, such as infant gender, gravidity, bed net use, maternal height, and maternal ethnic origin (Additional file 4), a similar RR (95% CI) was obtained: 0.72 (0.59–0.89; *P* = 0.002; Table 2). PP analyses yielded comparable results (Table 2).

**Table 2:** **LBW, preterm delivery, and mean birthweight, by treatment group**

**Outcome**	**ITT analysis**				**PP analysis**			
	**Control (SPCQ and placebo)**	**Intervention** **(SPAZ)**	**Risk ratio or ∆** **mean (95%** **CI)**	***P***	**Control (SPCQ and placebo)**	**Intervention** **(SPAZ)**	**Risk ratio or ∆** **mean (95%** **CI)**	***P***
LBW (Birthweight < 2,500 g), unadjusted	175/1,008 (17.4)	130/1,013 (12.8)	0.74 (0.60–0.91)	**0.005**	159/952 (16.7)	122/967 (12.6)	0.76 (0.61–0.94)	**0.011**
LBW (Birthweight < 2,500 g), adjusted^a^	–	–	0.72 (0.59–0.89)	**0.002**	–	–	0.72 (0.58–0.89)	**0.003**
Birthweight (g)	2,921.6	2,963.5	41.9	**0.049**	2,928.7	2,967.9	39.2	0.068
unadjusted	[2,890.1–2,953.1]	[2,936.0–2,991.0]	(0.2–83.6)		[2,986.9–2,960.4]	[2,940.3–2,995.4]	(−2.8–81.2)	
Birthweight (g)	2,916.6	2,969.0	52.4	**0.009**	2922.9	2974.1	51.2	**0.011**
adjusted^b^	[2,888.8–2944.3]	[2,941.4–2,996.6]	(13.2–91.6)		[2,895.0–2,950.9]	[2,946.4–3,001.8]	(11.7–90.6)	
PTD (<37 GWs), n (%), unadjusted, best available dating ultrasound	69/652 (10.6)	44/668 (6.6)	0.62 (0.43–0.89)	**0.010**	63/622 (10.1)	40/651 (6.1)	0.61 (0.41–0.89)	**0.009**

Data are number (%) or mean [95% CI], unless stated otherwise; ITT, Intention-to-treat; PP, Per-protocol; GW, Gestational weeks; CI, Confidence interval. *P* <0.05 marked in bold.

^a^Adjusted for infant gender, gravidity, no. of treatment courses (ITT only), clinic location, season of delivery, bed net use, mid-upper arm circumference, height, maternal highlander heritage.

^b^Adjusted for infant gender, gravidity, no. of treatment courses (ITT only), clinic location, mid-upper arm circumference, height, maternal highlander heritage.

**Figure 3:**
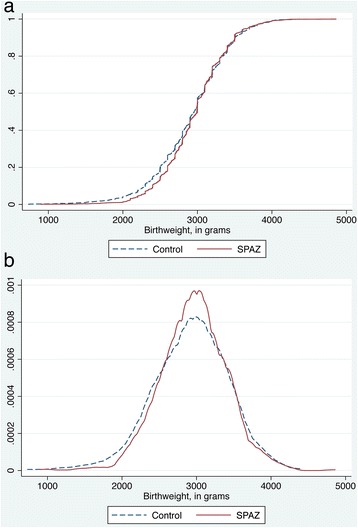
**Cumulative frequency (a) and Kernel density plot (b) of birthweight, by trial arm (ITT analysis).**

Mean birthweight was 41.9 g higher (95% CI: 0.2–83.6; *P* = 0.049) in the intervention arm, and when adjusted for potential confounders a similar result was obtained (52.4, 95% CI: 13.2–91.6, *P* = 0.009; Table 2). The observed difference in mean birthweight was largely explained by lower birthweights in the control compared to intervention in first quartile of the overall birthweight distribution (Mann Whitney-U test, *P* = 0.001; Figure 3). In a subset of women who had ultrasound pregnancy-dating, a reduction in PTD in the intervention arm was noted (RR: 0.62, 95% CI: 0.43–0.89, *P* = 0.010; ARR: 4.0%, 95% CI: 1.0–7.0; Table 2).

We observed no statistically proven interaction on LBW between the intervention and gravidity (*P* = 0.728), bed net use prior to enrolment (*P* = 0.172), maternal height (*P* = 0.818), and maternal highlander parentage (*P* = 0.238). Stratification for the aforementioned variables suggested that women who benefitted most may have been those in their first pregnancy, of low height, of highlander parentage, and who reported bed net use at enrolment (Additional file 4).

Women receiving the intervention were at lower risk of peripheral (RR: 0.57, 95% CI: 0.35–0.95; *P* = 0.029; ARR: 1.7%, 95% CI: 0.1–3.2) and placental blood parasitaemia (RR: 0.52, 95% CI: 0.28–0.97, *P* = 0.034; ARR: 1.6%, 95% CI: 0.1–3.2) as well as active placental infection (RR: 0.68, 95% CI: 0.47–0.98, *P* = 0.037; ARR: 2.9%, 95% CI: 0.2–5.5; Table 3). There was no significant difference in mean Hb or in proportion of women with anaemia (Table 3). The population-attributable fraction of LBW due to malaria was estimated at 7.4% overall, and 0.9% and 12.1% in the intervention and control arms, respectively.

**Table 3:** **Malaria infection and anaemia at delivery, by treatment groups**

**Outcome**	**ITT analysis**						**PP analysis**					
	**Control**		**Intervention**		**Risk ratio**	***P***	**Control**		**Intervention**		**Risk ratio**	***P***
	**(SPCQ and placebo)**		**(SPAZ)**		**(95%** **CI)**		**(SPCQ and placebo)**		**(SPAZ)**		**(95%** **CI)**	
Peripheral blood (light microscopy)												
All infections	40/1,022	(3.9)	23/1,023	(2.3)	0.57 (0.35–0.95)	**0.029**	39/962	(4.1)	22/972	(2.3)	0.56 (0.33–0.93)	**0.024**
*P. falciparum*	29	(2.8)	18	(1.8)	0.62 (0.35–1.11)	0.104	28	(2.9)	17	(1.8)	0.60 (0.33–1.09)	0.090
*P. vivax*	11	(1.1)	5	(0.5)	0.45 (0.16–1.30)	0.142	11	(1.1)	5	(0.5)	0.45 (0.16–1.29)	0.127
Placental blood (light microscopy)												
All infections	29/847	(3.4)	15/841	(1.8)	0.52 (0.28–0.97)	**0.035**	28/800	(3.5)	14/803	(1.7)	0.50 (0.26–0.94)	**0.028**
*P. falciparum*	25	(3.0)	15	(1.8)	0.60 (0.32–1.14)	0.115	24	(3.0)	14	(1.7)	0.58 (0.30–1.12)	0.098
*P. vivax*	1	(0.1)	0	(0.0)	–	–	1	(0.1)	0	(0.0)	–	–
Cord blood (light microscopy)^a^	4/744	(0.5)	3/744	(0.4)	0.75 (0.17–3.34)	1.000	4/695	(0.6)	3/716	(0.4)	0.73 (0.16–3.24)	0.681
Placental malaria (histology)												
Active and past infection^b^	150/736	(20.4)	128/736	(17.4)	0.85 (0.69–1.06)	0.143	138/697	(19.8)	122/702	(17.4)	0.88 (0.71–1.10)	0.245
Active infection^c^	65/736	(8.8)	44/736	(6.0)	0.68 (0.47–0.98)	**0.037**	62/697	(8.9)	41/702	(5.8)	0.66 (0.45–0.96)	**0.029**
Anaemia (Hb <11 g/dL)	689/935	(73.7)	700/933	(75.0)	1.02 (0.97–1.07)	0.508	647/883	(73.3)	663/889	(74.6)	1.02 (0.96–1.08)	0.532
Severe anaemia (Hb <7 g/dL)	44/935	(4.7)	37/933	(4.0)	0.84 (0.55–1.29)	0.432	39/883	(4.4)	34/889	(3.8)	0.87 (0.55–1.36)	0.531

Data are number (%). *P* <0.05 marked in bold.

^a^All *P. falciparum.*

^b^Detection of parasites or malaria pigment.

^c^Detection of parasites.

In self-collected vaginal swabs obtained from a subset of participants at second visit, carriage of *N. gonorrhoeae* was lower in women receiving SPAZ (SPCQ 8.2% [55/674] vs. SPAZ 5.4% [37/688], RR: 0.66, 95% CI: 0.44–0.99, *P* = 0.041), while carriage of *C. trachomatis* (SPCQ 4.5% [30/674] vs. SPAZ 3.8% [26/688], RR: 0.85, 95% CI: 0.51–1.42, *P* = 0.532) and *T. vaginalis* (SPCQ 21.8% [147/674] vs. SPAZ 21.5% [148/688], RR: 0.99, 95% CI: 0.81–1.21, *P* = 0.894) did not differ significantly.

We detected 204 maternal and 151 neonatal SAEs, none of which were drug-related and there were no significant differences in numbers between treatment groups (Table 4). There were three maternal deaths (all due, or probably due, to postpartum haemorrhage). We did not observe significant differences in the proportion of maternal deaths, miscarriages, stillbirths, congenital abnormalities, and neonatal deaths between trial arms (Table 4).

**Table 4:** **Safety of trial interventions: adverse events**

**Outcome**	**Control (SPCQ and placebo) n = 1,374**		**Intervention (SPAZ) n = 1,378**		**Risk ratio (95%** **CI)**	***P***
**All adverse events**	414	(30.1)	397	(28.8)	0.96 (0.85–1.07)	0.447
**All serious adverse events (SAE)**	174	(12.7)	181	(13.1)	1.04 (0.85–1.26)	0.712
*Maternal* ^a^	100	(7.3)	104	(7.5)	1.04 (0.80–1.35)	0.788
No. admitted^b^	94	(6.8)	90	(6.5)	0.96 (0.72–1.26)	0.745
No. of mothers with 2 SAEs	4	(0.3)	1	(0.1)	0.25 (0.03–2.23)	0.218
No. drug-related SAEs	0	(0.0)	0	(0.0)	–	–
*Characteristics of maternal SAEs* ^c^						
Maternal death	1	(0.1)	2	(0.2)	1.99 (0.18–22.0)	>0.999
Spontaneous abortion	4	(0.3)	1	(0.1)	0.24 (0.03–2.23)	0.218
Stillbirth	15	(1.1)	25	(1.8)	1.66 (0.88–3.14)	0.113
Emergency caesarean section	24	(1.8)	24	(1.7)	1.00 (0.57–1.75)	0.992
Hypertensive disorders of pregnancy	11	(0.8)	12	(0.9)	1.09 (0.48–2.46)	>0.999
Malaria	6	(0.4)	3	(0.2)	0.50 (0.12–1.99)	0.314
Other infections	10	(0.8)	6	(0.4)	0.60 (0.22–1.64)	0.330
Anaemia	3	(0.2)	2	(0.2)	0.66 (0.11–3.97)	0.687
Placenta praevia	2	(0.2)	3	(0.2)	1.50 (0.25–8.94)	>0.999
Antepartum haemorrhage	3	(0.2)	4	(0.3)	1.33 (0.30–5.93)	>0.999
Preterm labour	16	(1.2)	6	(0.4)	0.31 (0.11–0.85)	**0.034**
Preterm premature rupture of membranes	13	(1.0)	4	(0.3)	0.31 (0.10–0.94)	**0.030**
Prolonged prelabour rupture of membranes	2	(0.2)	1	(0.1)	0.50 (0.05–5.49)	0.624
Induction of labour (post-dates)	0	(0.0)	4	(0.3)	–	0.125
Postpartum haemorrhage	16	(1.2)	23	(1.7)	1.43 (0.76–2.70)	0.333
Other^d^	3	(0.2)	3	(0.2)	1.00 (0.20–4.93)	>0.999
*Neonatal*	74	(5.4)	77	(5.6)	1.04 (0.76–1.42)	0.816
No. admitted^b^	61	(4.4)	67	(4.9)	1.10 (0.78–1.54)	0.599
No. of babies with two SAEs	0	(0.0)	1	(0.0)	–	>0.999
No. drug-related SAEs	0	(0.0)	0	(0.0)	–	-
*Characteristics of neonatal SAEs* ^c^						
Congenital abnormality^e^	8	(0.6)	10	(0.7)	1.25 (0.49–3.14)	0.814
Neonatal death	19	(1.4)	11	(0.8)	0.58 (0.28–1.21)	0.140
Prematurity	15	(1.1)	9	(0.7)	0.60 (0.26–1.36)	0.227
Low birthweight	20	(1.5)	10	(0.7)	0.50 (0.23–1.06)	0.069
Infection	42	(3.1)	37	(2.7)	0.88 (0.57–1.36)	0.570
Birth asphyxia	16	(1.2)	18	(1.3)	1.12 (0.57–2.19)	0.736
Meconium aspiration syndrome	10	(0.7)	14	(1.0)	1.40 (0.62–3.13)	0.416
Cephalohaematoma	3	(0.2)	6	(0.4)	1.99 (0.50–7.96)	0.507
Jaundice	2	(0.2)	3	(0.2)	1.50 (0.25–8.94)	>0.999
**All non-serious adverse events (AEs)**	240	(17.5)	216	(15.7)	0.94 (0.80–1.11)	0.456
No. women with two AEs	10	(0.7)	13	(0.9)	1.30 (0.57–2.95)	0.535
No. drug-related maternal AE^f^	149	(10.8)	144	(10.5)	0.96 (0.78–1.20)	0.737
No. drug-related formal withdrawals^g^	7	(0.5)	8	(0.6)	1.14 (0.41–3.13)	>0.999
No. women with two drug-related AEs	3	(0.2)	9	(0.7)	2.99 (0.81–11.03)	0.145
*Characteristics of drug-related maternal AEs*						
Vomiting	82	(6.0)	74	(5.4)	0.90 (0.66–1.22)	0.498
Dizziness	56	(4.1)	33	(2.4)	0.59 (0.39–0.90)	**0.013**
Nausea	37	(2.7)	43	(3.1)	1.16 (0.75–1.79)	0.544
Pruritus	17	(1.2)	9	(0.7)	0.53 (0.24–1.18)	0.120
Weakness	13	(1.0)	13	(0.9)	1.00 (0.46–2.14)	0.994
Abdominal pain	3	(0.2)	12	(0.9)	4.32 (1.23–15.13)	**0.021**
Headache	8	(0.6)	5	(0.4)	0.62 (0.20–1.90)	0.422
Diarrhoea	1	(0.1)	3	(0.2)	2.99 (0.31–28.7)	0.625
Facial swelling (mild)	3	(0.2)	1	(0.1)	0.33 (0.04–3.19)	0.374
Feeling hot	3	(0.2)	0	(0.0)	–	0.124
Dyspepsia	0	(0.0)	4	(0.3)	–	0.125
Loss of appetite	0	(0.0)	2	(0.2)	–	0.500
Other	5	(0.4)	7	(0.1)	1.40 (0.44–4.39)	0.774

Data are n (%). Five women with SAEs, and six with drug-related AEs (occurring after administration of the first, correct, treatment) had unintentional treatment crossover and were analysed as per original assignment. *P* <0.05 marked in bold.

^a^Include stillbirths and miscarriages.

^b^Admission/prolongation of admission because of SAE.

^c^SAE reported because of one or more of the following.

^d^Trauma (2), vaginal haematoma (1), attempted suicide (1), gestational diabetes (1), hyperemesis gravidarum, possible appendicitis (1).

^e^Major: spina bifida (1), talipes equinovarus (5), cheilo- and palatoschisis [one with concomitant polydactyly] (2), prune belly syndrome (1), hypospadias (1), trisomy 21 (1), pulmonary atresia (1), multiple abnormalities of unknown cause (2), unilateral hand deformity (1) polydactyly (1), oligodactyly (1); minor: pectus carinatum (1).

^f^In the control group 19 women reported a reaction after taking placebo tablets.

^g^All due to nausea/vomiting after taking the study medication.

There were a total of 293 drug-related AEs. The overall number of drug-related AEs did not differ between treatment groups, despite the fact that women in the control arm received placebo following their baseline treatment course (*P* = 0.737; Table 4). Amongst women reporting drug-related AEs, common side effects included vomiting (5.7%; 156/2,752), dizziness (3.2%; 89/2,752), nausea (2.9%; 80/2,752), itching (1.0%; 26/2,752), weakness (1.0%; 26/2,752), and abdominal pain (0.6%; 15/2,752). Women receiving control treatment more frequently experienced dizziness (*P* = 0.013) and abdominal pain was more commonly reported by women who had SPAZ (*P* = 0.021; Table 4).

Amongst women in the SPAZ arm, there was no difference in the proportion of women-infant pairs affected by at least one SAE (1 treatment: 14/117 [12.0%], 2 treatments: 23/223 [10.3%], 3+ treatments: 122/1,038 [11.8%], *P* [comparison across groups] = 0.821), and there was no difference across groups when maternal SAEs (1 treatment: 13/117 [11.1%], 2 treatments: 15/223 [6.7%], 3+ treatments: 75/1,038 [7.2%], *P* [comparison across groups] = 0.285) and neonatal SAEs (1 treatment: 4/117 [3.4%], 2 treatments: 12/223 [5.4%], 3+ treatments: 60/1,038 [5.8%], *P* [comparison across groups] = 0.567) were evaluated separately. Similarly, the proportion of women-infant pairs affected by at least one SAE did not differ significantly by number of treatment visits in the control arm (1 visit: 14/144 [9.7%], 2 visits: 17/224 [7.6%], 3+ visits: 116/1,006 [11.5%], *P* [comparison across groups] = 0.208).

The proportion of women reporting at least one drug-related AE increased with number of doses received, both for SPAZ (1 dose: 4/117 [3.4%], 2 doses: 15/223 [6.7%], 3+ doses: 116/1,038 [11.2%], *P* = 0.007) and SPCQ/placebo (1 dose: 7/144 [4.9%], 2 doses: 12/224 [5.4%], 3+ doses 127/1,006 [10.6%], *P* <0.001). Only 1.9% (47/2,466) and 1.1% (24/2,133) of women reported not taking the remaining doses of the treatment courses provided at enrolment and second study visit courses (AZ or CQ/placebo CQ), respectively. There were no significant differences in adherence between treatment arms (enrolment treatment course: intervention 1.8% [22/1,255] vs. control 2.1% [25/1,211], *P* = 0.572; second visit treatment course: intervention 1.2% [13/1,081] vs. control 1.1% [11/1,052], *P* = 0.446).

## Discussion

We found that IPTp with SPAZ significantly reduced the risk of LBW and PTD, and increased mean birthweight compared to a single treatment course of SPCQ. Both treatments were well tolerated.

To our knowledge, this is the first successful trial of IPTp outside of sub-Saharan Africa, or in an area where both *P.f.* and *P.v.* are endemic. The intervention reduced malaria infection at delivery, yet overall prevalence was substantially lower than previously reported [23]. IPTp with SPAZ appears to be beneficial against malaria in settings like PNG, although there were too few *P.v.* infections to evaluate the effect of the intervention on these. HIV infection is uncommon in Madang, precluding assessment of SPAZ in HIV-infected women.

Few participants reported problems with adherence to either study regime, despite the high dose of AZ. We only observed the first dose of each treatment course, and did not measure drug levels, hence non-adherence may be underestimated. The proportion of women reporting side effects in the intervention arm was small, despite the high dose of azithromycin (4 g) compared to other trials of IPTp testing AZ-based combinations [11,15,35]. This might be due to our split daily dosing, choice of partner drug for AZ, and provision of a dry biscuit prior to treatment at the antenatal clinic. Furthermore, the number of episodes during which women reported side-effects were similar between both groups, even though women randomised to the control arm had received placebo medication at follow-up visits.

The beneficial effect of SPAZ on LBW and placental malaria may, in part, be because of an imbalance in trial design as women in the intervention arm received more SP doses. Our control group was designed to match the current PNG protocol when the trial was designed. Although PNG policy now advocates three monthly doses of SP, a direct comparison with this regime is not possible: the impact of monthly SP on LBW in PNG is unknown. A recent meta-analysis demonstrated that three or more doses of SP are better than one or two for the prevention of malaria and LBW in African women [36]. If SPAZ were compared to monthly SP, the effect on LBW might be less striking. SP has not been shown to prevent PTD [37], which was significantly less frequent in women receiving SPAZ than SPCQ. The small number of women who received only one course of SPAZ or SPCQ and were followed up for the primary outcome (n = 75) precludes further analysis to quantify the benefit of adding AZ to SP. Interaction and stratified analyses did not indicate a difference in the efficacy of treatment by gravidity: the study was not powered and was not designed to convincingly demonstrate this.

The low overall prevalence of malaria (and low population attributable fraction of LBW due to malaria), as well as the reduction in PTD in the intervention arm, suggests that the effect of SPAZ on reducing LBW is mediated by other mechanisms in addition to its antimalarial effect. A greater impact on LBW might be observed in settings where malaria prevalence is higher than in this study, and women share other common risk factors. SPAZ may prevent LBW by clearing STIs and ascending reproductive tract infections [22], which are common in PNG [24]. Vaginal swabs were collected from a subset of 400 study participants at enrolment. Of these, 11% carried *C. trachomatis*, and 10% carried *N. gonorrhoeae* [29]*.* There were only modest differences in carriage of *C. trachomatis* and *N. gonorrhoeae* at second treatment visit between women receiving SPAZ and SPCQ. Carriage of *N. gonorrhoeae* was lower in women receiving SPAZ, while carriage of *C. trachomatis* did not differ significantly by treatment arm. This suggests that an impact on STI carriage explains only a part of the positive effect of SPAZ on LBW. AZ may also exert immunomodulatory effects favouring fetal growth [38].

Two previous studies of SPAZ-IPTp in rural Malawi gave conflicting results. In one, monthly SP and 1 g of AZ given twice reduced the risk of PTD, LBW, and malaria compared to two doses of SP, to a similar extent to our study [11,12]. In the other, addition of AZ 1 g twice to three doses of SP-IPTp did not significantly reduce PTD or malaria; LBW was not reported and mean birthweight was 40 g higher in the AZ arm [35]. Both studies used lower doses of AZ than our trial (4 g). Taken together, the studies suggest SPAZ may have beneficial effects on PTD, depending on the population characteristics.

One concern with use of SP is emergence of drug resistance that limits efficacy or could even exacerbate infection [39]. Parasites from this study have not been typed for drug resistance markers, but contemporaneous parasites from children and adults in the same locale have recently been analysed [18]. ‘Highly resistant’ patterns of molecular markers (quintuple mutations in the *dhfr* and *dhps* genes) and ‘super resistant’ parasites (also featuring *dhfr* 164 or *dhps* 581 mutations) [21] have not been detected, and the prevalence of *dhps* 540 mutations associated with drug failure in young children [21] was <20%. By contrast, *P.v.* is frequently resistant to SP [18], but was rare in this cohort.

Potential adverse consequences of AZ use may include selection for (probably temporary) increases in carriage of AZ-resistant organisms, especially *Streptococcus pneumoniae* [40], and a possible association between macrolide use in late pregnancy and increased risk of infantile hypertrophic pyloric stenosis [41]. Such potential effects require careful monitoring in future studies of AZ for indications such as IPTp.

There are several limitations to our study. First, birthweights eligible for inclusion in the primary outcome analysis were available for only 72% of women randomised to treatment. Reassuringly, loss to follow-up rates did not differ between control and intervention arm. Furthermore, there were no major differences in the background characteristics of women that were lost to follow-up when compared to those who were not (Additional file 4). However, women lost to follow-up were more likely to be young and malaria infected at enrolment. It is hence possible that this may have led to an underestimation of the effect of SPAZ on LBW, given these women are at higher risk of placental malaria [42], and may be at higher risk of carrying bacterial STIs [43], and so would benefit most from the intervention. Furthermore, the reasons for not presenting for delivery remain unknown for 474 women, which may include AEs. Second, the proportion of primigravidae was high (50.2%), suggesting that there was selection bias, which could result in overestimating the effect SPAZ had on LBW. Third, only one quarter of women screened were ultimately enrolled and randomised, the principal reason for exclusion being presentation at advanced GA. This raises questions as to the representativeness of the study sample. Due to logistic reasons, we were unable to collect background demographic data for a substantial proportion of those women that were screened but not enrolled. It is possible that these women were more likely to be multigravid, and were more likely to be excluded because they tended to present at more advanced gestation. This may result in overestimation of the effect, although this could be less marked in circumstances of relatively low malaria prevalence. Not all women had ultrasound dating, and amongst those who did, many presented relatively late: use of later scans will underestimate GA in small-for-gestational-age babies and overestimate GA in macrosomic babies. Reassuringly, there was a similar degree of reduction in PTD in the SPAZ arm amongst women who had early dating scans, although this was not statistically significant (due to a lack of power) (SPCQ 6.6% [23/333] vs. SPAZ 3.9% [13/327], RR: 0.58, 95% CI: 0.30–1.12, *P* = 0.097). In addition, we did not measure in detail some potentially important confounders, including women’s socioeconomic status. Lastly, due to funding constraints, we were unable to evaluate the impact of treatment on reducing chorioamnionitis, an important risk factor for PTD. Strengths of the study include the large sample size, random group allocation, the demonstration of benefit from IPTp in a setting outside Africa, and the impact of SPAZ on clinically-important endpoints of LBW and PTD.

The main reason for not meeting trial eligibility criteria was advanced GA, and there was a treatment-independent benefit of number of study visits in reducing LBW (Additional file 4). The more often, and earlier, women attend antenatal care, the more they will benefit from interventions that may reduce the risk of LBW other than IPTp, as well as maximise the benefit from IPTp [36]. It is therefore of utmost importance that access to, and early first attendance at, antenatal care is improved whilst interventions to improve birth outcomes are rolled out to prevent compromising the effectiveness of IPTp. Women excluded from the primary outcome analysis tended to be younger and were more likely to be illiterate and reside in rural areas; such women might derive most benefit from interventions such as IPTp, especially when combined with early attendance at antenatal clinics [44].

## Conclusions

Our findings suggest that IPTp with SPAZ reduces the risk of LBW in a setting of low-to-moderate malaria prevalence; it might have greater benefit in areas with higher malaria burden. Future research will evaluate the impact of SPAZ on pneumococcal antibiotic resistance, the latter being a potential barrier to implementation. Promising interventions to reduce LBW and PTD in countries such as PNG are rare; SPAZ is one such candidate worthy of further evaluation.

## Additional files

Additional file 1:
**Study protocol.**
Additional file 2:
**CONSORT checklist.**
Additional file 3:
**Map of study area including location of recruitment antenatal clinics.**
Additional file 4: Table S1. Comparison of baseline characteristics of study participants included in, and excluded from, the intention-to-treat (ITT) birthweight analysis. **Table S2.** Comparison of baseline characteristics of study participants excluded from the ITT birthweight analysis, by treatment arm (n = 754). **Table S3.** Factors associated with low birthweight on crude analysis. **Table S4.** Stratified analysis of low birthweight for gravidity, maternal height, maternal ethnic parentage, and bed net use before enrolment, by treatment group.

## Abbreviations

AEs: Adverse events
ARR: Absolute risk reduction
AZ: Azithromycin
CQ: Chloroquine
DSMB: Data Safety Monitoring Board
GA: Gestational age
IPTp: Intermittent preventive treatment of malaria in pregnancy
ITT: Intention-to-treat
LBW: Low birthweight
*P.f.*: *Plasmodium falciparum*
PNG: Papua New Guinea
PNGIMR: PNG Institute of Medical Research
PP: Per-protocol
PTD: Pre-term delivery
*P.v.*: *P. vivax*
RR: Risk ratios
SAE: Serious adverse event
SPAZ: AZ combined with SP
SP: Sulphadoxine-pyrimethamine
STIs: Sexually transmitted infections
