# Supplementary material for: Sulphadoxine-pyrimethamine plus azithromycin for the prevention of low birthweight in Papua New Guinea: a randomised controlled trial
Source: BMC Med. 2015 Jan 16;13:9. doi: 10.1186/s12916-014-0258-3 (PMC4305224; doi:10.1186/s12916-014-0258-3)
Supplement: Additional file 1: — Study protocol. [file 12916_2014_258_MOESM1_ESM.doc]

**Intermittent preventive treatment with azithromycin-containing regimens for the prevention of malarial infections and anaemia and the control of sexually transmitted infections in pregnant women in Papua New Guinea.**

**Sponsored by:**

University of Melbourne and Papua New Guinea Institute of Medical Research

**Principal Investigators:**

Stephen J Rogerson, FRACP PhD

Ivo Mueller, PhD

Peter Siba, PhD

Clara Menendez, MD PhD

**Co-Investigators**

James Beeson, FRACP, PhD

Graham Brown, FRACP MPH PhD

Timothy Davis, FRACP PhD

Harin Karunajeewa, FRACP

Lahui Gaita, MBBS MMed

Glen Mola, FRANZCOG

Peter Zimmerman, PhD

**Funded by:**

Bill & Melinda Gates Foundation, Global Health Program

European Union Framework Programme 7

**Draft or Version Number:**

Version 1.5

**Day Month Year**

14/07/2009

Statement of Compliance

The study will be carried out in accordance with Good Clinical Practice (GCP) as required by the following:

- ICH GCP E6
- Completion of Human Subjects Protection Training

SIGNATURE PAGE

The signatures below document the approval of this protocol and the attachments, and provide the necessary assurances that this trial will be conducted according to all stipulations of the protocol, including all statements regarding confidentiality and according to local legal and regulatory requirements and to the principles outlined in applicable U.S. federal regulations and ICH guidelines.

| Sponsors Representative (PNG Institute of Medical Research) | | | |
| --- | --- | --- | --- |
| Signed: |  | Date: |  |
|  | Name Peter Siba  Title Prof, Director PNG Institute of Medical Research |  |  |
| Principal Investigator –, Department of Medicine, University of Melbourne: | | | |
| Signed: |  | Date: |  |
|  | Name Stephen Rogerson  Title A/Prof |  |  |
| Principal Investigator / Project Leader –Papua New Guinea Institute of Medical Research: | | | |
| Signed: |  | Date: |  |
|  | Name Ivo Mueller,  Title Dr. |  |  |

[1 Key Roles 1](#__RefHeading___Toc189366799)

[2 Background Information and Scientific Rationale 4](#__RefHeading___Toc189366800)

[2.1 Background Information 4](#__RefHeading___Toc189366801)

[2.2 Rationale 9](#__RefHeading___Toc189366802)

[2.3 Potential Risks and Benefits 10](#__RefHeading___Toc189366803)

[2.3.1 Potential Risks 10](#__RefHeading___Toc189366804)

[2.3.2 Known and Potential Benefits 11](#__RefHeading___Toc189366805)

[3 Objectives 13](#__RefHeading___Toc189366806)

[3.1 Objectives 13](#__RefHeading___Toc189366807)

[3.2 Outcome measures 14](#__RefHeading___Toc189366808)

[Primary Outcome Measure 14](#__RefHeading___Toc189366809)

[Secondary Outcome Measures 15](#__RefHeading___Toc189366810)

[4 Study Design 16](#__RefHeading___Toc189366811)

[5 Study Population 18](#__RefHeading___Toc189366812)

[5.1 Selection of the Study Population 18](#__RefHeading___Toc189366813)

[5.2 Inclusion/Exclusion Criteria 18](#__RefHeading___Toc189366814)

[6 Study Procedures/Evaluations 20](#__RefHeading___Toc189366815)

[6.1 Study Procedures 20](#__RefHeading___Toc189366816)

[6.1.1 Field Study Team 21](#__RefHeading___Toc189366817)

[6.1.2. Participant Database and Study Visit Activities 22](#__RefHeading___Toc189366818)

[6.1.3 First contact and pre-screening 22](#__RefHeading___Toc189366819)

[6.1.4 Enrolment and Treatment 22](#__RefHeading___Toc189366820)

[6.1.5 Delivery and Assessment of IPTp intervention 26](#__RefHeading___Toc189366821)

[6.1.6 Long-term follow-up following intervention 28](#__RefHeading___Toc189366822)

[6.1.7 Passive Surveillance 29](#__RefHeading___Toc189366823)

[6.1.8 Treatment of Sexually Transmitted Infections 32](#__RefHeading___Toc189366824)

[6.2 Laboratory Evaluations 32](#__RefHeading___Toc189366825)

[6.2.1 Laboratory Evaluations/Assays 32](#__RefHeading___Toc189366826)

[6.2.2 Special Assays or Procedures 35](#__RefHeading___Toc189366827)

[6.2.3 Specimen Collection, Preparation, Handling and Shipping 35](#__RefHeading___Toc189366828)

[6.3 Randomisation and Blinding 37](#__RefHeading___Toc189366829)

[6.3.1 Randomisation procedures 37](#__RefHeading___Toc189366830)

[6.3.2 Pre-packaging of drugs 38](#__RefHeading___Toc189366831)

[6.3.3 Allocation Concealment and Blinding 38](#__RefHeading___Toc189366832)

[7 Study Schedule 39](#__RefHeading___Toc189366833)

[7.1 Enrolment 40](#__RefHeading___Toc189366834)

[7.2 Treatment & Follow-up Visits 40](#__RefHeading___Toc189366835)

[7.3 Early Termination Visit 42](#__RefHeading___Toc189366836)

[7.4 Criteria for Discontinuation or Withdrawal of a Subject 42](#__RefHeading___Toc189366837)

[8 Assessment of Outcome MeasureS 43](#__RefHeading___Toc189366838)

[8.1 Specification of the Appropriate Outcome Measures 43](#__RefHeading___Toc189366839)

[8.1.1 Primary Outcome Measures 43](#__RefHeading___Toc189366840)

[8.1.2 Secondary Outcome Measures 43](#__RefHeading___Toc189366841)

[9 Safety assessment and reporting 46](#__RefHeading___Toc189366842)

[9.1 Definition of Adverse Event (AE) 46](#__RefHeading___Toc189366843)

[9.2 Definition of Serious Adverse Event (SAE) 47](#__RefHeading___Toc189366844)

[9.3 Reporting Procedures 47](#__RefHeading___Toc189366845)

[9.3.1 Serious Adverse Event Detection and Reporting 47](#__RefHeading___Toc189366846)

[9.3.2 Procedures to be followed in the Event of Abnormal Laboratory Test Values or Abnormal Clinical Findings 48](#__RefHeading___Toc189366847)

[9.3.3 Type and Duration of the Follow-up of Subjects after Adverse Events 48](#__RefHeading___Toc189366848)

[9.4 Halting Rules 49](#__RefHeading___Toc189366849)

[10 Clinical Monitoring Structure 50](#__RefHeading___Toc189366850)

[10.1 Site Monitoring Plan 50](#__RefHeading___Toc189366851)

[11 Statistical Considerations 51](#__RefHeading___Toc189366852)

[11.1 Study Outcome Measures 51](#__RefHeading___Toc189366853)

[11.1.1 Definitions 51](#__RefHeading___Toc189366854)

[11.1.2 Primary Outcome Measures 53](#__RefHeading___Toc189366855)

[11.1.3 Secondary Outcome Measures 53](#__RefHeading___Toc189366856)

[11.2 Sample Size Considerations 54](#__RefHeading___Toc189366857)

[11.3 Participant Enrolment and Follow-Up 56](#__RefHeading___Toc189366858)

[11.4 Analysis Plan 57](#__RefHeading___Toc189366859)

[11.4.1 Primary Outcome Measures 57](#__RefHeading___Toc189366860)

[11.4.2 Secondary Outcome Measures 57](#__RefHeading___Toc189366861)

[12 Access to Source Data/Documents 58](#__RefHeading___Toc189366862)

[13 Quality Control and Quality Assurance 59](#__RefHeading___Toc189366863)

[14 Ethics/Protection of Human Subjects 60](#__RefHeading___Toc189366864)

[14.1 Declaration of Helsinki 60](#__RefHeading___Toc189366865)

[14.2 Institutional Review Board 60](#__RefHeading___Toc189366866)

[14.3 Informed Consent Process 60](#__RefHeading___Toc189366867)

[14.4 Exclusion of Women, Minorities, and Children (Special Populations) 62](#__RefHeading___Toc189366868)

[14.5 Subject Confidentiality 62](#__RefHeading___Toc189366869)

[14.6 Future Use of Stored Specimens 63](#__RefHeading___Toc189366870)

[15 Data Handling and Record Keeping 64](#__RefHeading___Toc189366871)

[15.1 Data Management Responsibilities 64](#__RefHeading___Toc189366872)

[15.2 Data Capture Methods 65](#__RefHeading___Toc189366873)

[15.3 Types of Data 65](#__RefHeading___Toc189366874)

[15.4 Timing/Reports 66](#__RefHeading___Toc189366875)

[15.5 Study Records Retention 66](#__RefHeading___Toc189366876)

[15.6 Protocol Deviations 66](#__RefHeading___Toc189366877)

[16 Publication Policy 68](#__RefHeading___Toc189366878)

[17 Preparatory study 69](#__RefHeading___Toc189366879)

[18 Data and Sample Sharing with P. vivax in Pregnacy (Pregvax) study 70](#__RefHeading___Toc189366880)

[19 Literature References 72](#__RefHeading___Toc189366881)

[20 SUPPLEMENTS/APPENDICES 75](#__RefHeading___Toc189366882)

[I. Study Follow-up Schedule Diagram 76](#__RefHeading___Toc189366883)

[II. Clinical Definitions 77](#__RefHeading___Toc189366884)

[III. Informed Consent – Study Information Brochure (English) 79](#__RefHeading___Toc189366885)

[IV. Informed Consent – Signature Form (English) 88](#__RefHeading___Toc189366886)

[V. Preliminary Study: Informed Consent – Study Information Brochure (English) 91](#__RefHeading___Toc189366887)

[VI. Preliminary Study: Informed Consent – Signature Form (English) 96](#__RefHeading___Toc189366888)

| AE | Adverse Event |
| --- | --- |
| ART | Artesunate |
| AZ | Azithromycin |
| BCG | Bacillus Calmette-Guerin (Tuberculosis Vaccine) |
| BI | Burnet Institute |
| CGHD | Centre of Global Health & Disease, Case Western Reserve University |
| CQ | Chloroquine |
| CRF | Case Report Form |
| DALY | Disability- Adjusted Life-Year |
| DHFR | Dihydrofolate reductase |
| DHPS | Dihydropteroate synthase |
| DSMB | Data and Safety Monitoring Board |
| EPI | Extended Program of Immunization |
| EU | European Union |
| FGR | Fetal growth restriction |
| FP7 | Framework programme 7 |
| GA | Gestational assessment |
| GCP | Good Clinical Practice |
| GW | Gestation weeks |
| Hb | Haemoglobin |
| HC | Health Centre |
| HEO | Health Extension Officer |
| HIV | Human Immunodeficiency Virus |
| HPLC | High performance liquid chromatography |
| IPTp | Intermittent Preventive Treatment in Pregnancy |
| ITN | Insecticide Treated Net |
| LDR | Ligase Detection Reaction |
| LM | Light microscopy |
| LMP | Last menstrual period |
| MalariaGEN | Malaria Genomic Epidemiology Network |
| MCH | Maternal and child health |
| MSP1 | Merozoite Surface Protein 1 |
| MSP2 | Merozoite Surface Protein 2 |
| MOI | Multiplicity of Infection |
| N | Number (typically refers to subjects) |
| NIGH | Nossal Institute of Global Health |
| OPV | Oral Polio Vaccine |
| *P. falciparum* | Plasmodium falciparum |
| *P. vivax* | Plasmodium vivax |
| PCR | Polymerase Chain Reaction |
| PBMC | Peripheral Blood Mononuclear Cells |
| PET | Pre-eclamptic toxaemia |
| PI | Principal Investigator |
| PK | Pharmacokinetics |
| PNG | Papua New Guinea |
| PNGIMR | PNG Institute of Medical Research |
| PTD | Pre-term delivery |
| RDT | Rapid Diagnostic Test |
| RPR | Rapid plasma reagin |
| SAE | Serious Adverse Event |
| SOP | Standard Operating Procedure |
| SP | Sulfadoxine-pyrimethamine |
| STI | Sexually Transmitted Infections |
| TPHA | Treponema pallidum haemagglutination assay |
| UoM | University of Melbourne |
| USS | Ultrasound scan |
| UWA | University of Western Australia |
| VCT | Voluntary counselling and testing (for HIV) |
| WAZ | Weight-for-Age nutritional Z-score |
| WEHI | The Walter and Eliza Hall Institute of Medical Research |
| WHO | World Health Organization |

**Title**: Intermittent preventive treatment with azithromycin-containing regimes for the prevention of malaria and anaemia and the control of sexually transmitted infections in pregnant women in Papua New Guinea

**Population**: 2792 pregnant women who are residents of malaria-endemic areas of Madang Province, in Papua New Guinea.
50 non-pregnant community controls

**Number of Sites**: 7 field sites (Modilon Hospital, Sisirak, Jumba and Town Clinics, Madang, and Mugil, Alexishafen and Yagaum Health Centres, PNG), 5 laboratory sites (Goroka and Madang, PNG; University of Melbourne and WEHI, Melbourne, Australia; Case Western Reserve University, Cleveland USA, Hospital Clinic, Barcelona)

**Study Duration**: 48 months

**Subject Duration**: up to 18 months

**Objectives**:

The **primary** **objective** of this project is to:

1. Determine whether IPT with SP + AZ is safe and whether it decreases the incidence of low birth weight (LBW; <2500 g) compared to standard treatment (i.e. a single treatment course of SP and chloroquine at booking, in conjunction with ITN use).

**Secondary objectives** of the study include:

1. To compare mean birth weight of babies born to women on SP+AZ and controls.
2. To examine the effect of SP + AZ on prevalence of *P falciparum* at delivery in peripheral, placental and cord blood films and on placental histology, and at second visit by PCR only in peripheral blood films.
3. To compare maternal haemoglobin concentrations and anaemia prevalence and severity between groups
4. To investigate the prevalence and consequences (maternal haemoglobin, birth weight and placental pathology) of *P. vivax* infection in pregnancy
5. To study immune responses to *P. vivax* in pregnant women and non-pregnant women, with and without current *P. vivax* infection
6. To determine the incidence of symptomatic malaria by passive case detection in each group.
7. To compare the proportion of women carrying azithromycin-sensitive sexually transmitted infections at second treatment visit between groups.
8. To document the incidence and severity of Adverse Events possibly or probably associated with study medications
9. To examine the prevalence of drug resistance markers in parasites infecting women in late pregnancy
10. To measure the prevalence and antibiotic sensitivity patterns of *S. pneumoniae* in nasopharyngeal swabs collected at delivery
11. To measure the impact of IPTp on development of immunity to malaria in pregnancy
12. To document maternal, perinatal and infant mortality rates in each group.

**Schematic of Study Design**: See Appendix I

# Key Roles

**Individuals**: **Principal Investigators**:

**Stephen Rogerson, FRACP, PhD**, Department of Medicine (RMH/WH), University of Melbourne, 4th Floor Clinical Science Building, Royal Parade, Parkville, VIC 3050, Australia. Tel. +61 3 8344 3259, Fax: +61 3 9347 1863, email sroger@unimelb.edu.au

**Ivo Mueller, PhD**, Project Leader, Scientific Head Vector Borne Disease Unit, PNGIMR, P.O. Box 60, Goroka, Papua New Guinea, Tel. +675 732 2800 ext 234, Fax: +675 732 1998, email ivomueller@fastmail.fm

**Peter Siba, PhD**, Director and Prof. of Health Research, PNGIMR, P.O. Box 60, Goroka, Papua New Guinea, Tel. +675 732 1469, Fax: +675 732 1998, email [Peter.Siba@pngimr.org.pg](mailto:Peter.Siba@pngimr.org.pg)

**Clara Menendez, MD PhD**, Fundació Clínic per a la Recerca Biomèdica,

Barcelona email Menendez@clinic.ub.es

**Co - Investigators**:

**James Beeson, FRACP PhD**, Dept. of Infections & Immunity, Walter & Eliza Hall Institute of Medical Research, 1 G Royal Parade, Parkville, VIC 3050, Australia, Tel. +61 3 9345 2555 Fax: +61 3 9347 0852, email [beeson@wehi.edu.au](mailto:beeson@wehi.edu.au)

**Graham Brown, FRACP MPH PhD**, Nossal Institute of Global Health, University of Medicine, Parkville VIC 3010. Tel + 61 3 8344 5490 Fax + 61 3 9347 1863 email: gvb@unimelb.edu.au

**Tim Davis, Prof, FRACP PhD.,** School of Medicine & Pharmacology, University of Western Australia, Fremantle Hospital, PO Box 480, Fremantle 6959, Western Australia. T: +618 9431 3229, F: +618 9431 2977, e: [tdavis@cyllene.uwa.edu.au](mailto:tdavis@cyllene.uwa.edu.au)

**Harin Karunajeewa, FRACP**, School of Medicine & Pharmacology, University of Western Australia, Fremantle Hospital, PO Box 480, Fremantle 6959, Western Australia. **T**: +618 9431 3229, **F**: +618 9431 2977, **E**: [harin@cyllene.uwa.edu.au](mailto:harin@cyllene.uwa.edu.au)

**Glen Mola, FRANZCOG,** Department of Obstetrics and Gynaecology, University of Papua New Guinea

**Lahui Gaita, MBBS MMed,** Chief Obstetrician, Modilon Hospital, PO Box 2119, Madang, MAD 511 Papua New Guinea **T** +852 2022 - **F:** +852 3716

**Peter Zimmerman, PhD,** Center for Global Health and Diseases, Case Western Reserve University, CASE School of Medicine, 10900 Euclid Avenue, Wolstein Research Building, 4th Floor, Cleveland, Ohio, 44106-7286, USA. T: +1 216-368-0508, F: +1 216-368-4825, e: [peter.zimmerman@case.edu](mailto:peter.zimmerman@case.edu)

**Medical Monitors:**The following will compose the Data Safety Monitoring Board for the study:

Dr James McCarthy, Queensland Institute of Medical Research

Dr Inoni Betuela, Papua New Guinea Institute of Medical Research

Dr Julie Simpson, MEGA, School of Population Health, University of Melbourne (Biostatistician)

**Institutions**:

**Department of Medicine (RMH/WH), University of Melbourne**, 4th Floor Clinical Science Building, Royal Parade, Parkville, VIC 3050, Australia, Associate Professor Stephen Rogerson

**PNG Institute of Medical Research (PNGIMR)**, P. O. Box 60, Goroka, Papua New Guinea, Prof. Peter Siba & Dr. Ivo Mueller

**Fundació Clínic per a la Recerca Biomèdica (FCRB)** Profesor Clara Menendez

**The Walter & Eliza Hall Institute of Medical Research**, 1 G Royal Parade, Parkville, VIC 3050, Australia, Dr. James Beeson

**Scho**o**l of Medicine & Pharmacology, University of Western Austral**ia, Fremantle Hospital, PO Box 480, Fremantle 6959, Western Australia. Prof Tim Davis and Dr Harin Karunajeewa.

**Nossal Institute for Global Health, University of Melbourne**, Alan Gilbert Building, University of Melbourne, VIC 3010. Prof Graham Brown.

**Centre of Global Health & Diseases (CGHD), Case Western Reserve University**, CASE School of Medicine, 10900 Euclid Avenue, Wolstein Research Building, 4th Floor, Cleveland, Ohio 44106-7286, USA, A/Prof. Peter Zimmerman

**Protocol Statisticians:**

Dr Ivo Mueller

An additional full time statistician to be appointed for years 3 and 4.

**Protocol Data Manager:**

**Thomas Adiguma**, BSc, PNG Institute of Medical Research, P.O. Box 378, Madang, MAD 511, Papua New Guinea, T: +675-852-2909, F: +675-852-3289, e: [Thomas.Adiguma@pngimr.org.pg](mailto:Thomas.Adiguma@pngimr.org.pg)

**Protocol Epidemiologist:**

Dr Ivo Mueller

**Protocol Clinicians:**

Senior clinician: Dr Sarah Hanieh, FRACP

Junior clinicians: Dr Maria Ome MBBS

Dr Regina Wangnapi MBBS.

**Community Advisory Board Liaison:**

**Dr Ivo Mueller and John Taime**, Administrative Manager, PNG Institute of Medical Research, P.O. Box 378, Madang, MAD 511,Papua New Guinea T: +675-852-2909, F: +675-852-3289, [John.Taime@pngimr.org.pg](mailto:John.Taime@pngimr.org.pg)

# Background Information and Scientific Rationale

## Background Information

**Burden of malaria in pregnancy**

In malaria-endemic areas, young children and pregnant women are at especially high risk for malaria morbidity and mortality. Consequences of malaria in pregnancy in endemic areas include maternal anaemia (predisposing to maternal mortality) and low birth weight (LBW, predisposing to infant mortality). Globally, over 40 million women are exposed to the risk of malaria infection when pregnant each year, and up to 200,000 infant deaths and 10,000 maternal deaths may be consequent upon malaria in pregnancy ^1,2^.

**Burden of malaria in PNG**

In Papua New Guinea (PNG), malaria is the leading cause of outpatient attendances nationally, the third commonest cause of admission, second commonest cause of death, and causes the greatest burden of DALYs (at 4894/100,000 per year). Maternal deaths (to which malaria in pregnancy contributes significantly) are the fifth leading cause of DALYs. Our intervention is highly likely to benefit the population of PNG, and addresses a PNG Government-identified major health issue. Precise estimates of the burden of malaria in pregnancy are not available, but comparative studies between malaria-endemic coastal PNG and the malaria-free highlands suggest malaria is responsible for significant proportions of anemia and LBW in pregnancy in coastal areas ^3^. In published studies, 70% of Madang women were anemic, 17% had severe anemia (Hb <7 g/dl), and 20% had LBW babies. More recent data show 59% of women are anemic, hemoglobin <10 g/dl; 13% have severe anemia (hemoglobin <7 g/dl) and 22% of babies were LBW^4^. This is among women receiving current standard PNG treatment including SP and chloroquine treatment and chloroquine weekly, which was the control regime in PNG until this year. Maternal haemoglobin concentrations and infant birth weights were lower when the placenta was malaria infected than when it was not ^5^. It is known that *P. falciparum* is a major cause of low birth weight and maternal anaemia, but what is less well understood is the consequences of *P vivax* infection in pregnancy. *P. vivax*  is common in pregnant women in PNG.

**Tools to control malaria in pregnancy**

Presently we have two principal tools to control malaria in pregnancy, and to reduce related mortality: insecticide-treated materials, and antimalarial drugs, given as regular prophylaxis or as intermittent preventive treatment for pregnancy (IPTp). PNG is now supplying long-lasting ITNs to all malaria-exposed individuals through the Global Fund; given the very high levels of chloroquine resistance and poor compliance of women with weekly CQ, ITNs are now seen as a preferred alternative to chloroquine prophylaxis. IPTp involves the regular administration of treatment doses of an appropriate antimalarial agent, at 2 or more time points during pregnancy (WHO Draft Strategic Framework). The leading candidate for IPTp in Africa is sulfadoxine-pyrimethamine (SP). In several studies, SP in two or more doses decreased malaria prevalence in peripheral and placental blood and reduces maternal anaemia and improves birth weight ^6-10^. The risk of malaria is highest in first pregnancy, and some suggest limiting prophylaxis to this group. However, drugs that effectively prevent malaria may also restrict the development of immunity to pregnancy malaria, placing women in later pregnancies at risk of disease. The interplay between drug control of disease and immune response is presently poorly understood. There is presently no information on the impact of IPTp on *P vivax* in pregnancy. If vivax infection causes significant morbidity, choice of antimalarials will need to be guided both by drug sensitivity of *P falciparum* and by *P vivax* sensitivity.

**Drug resistance**

Resistance of *P falciparum* to SP is common in parts of South East Asia and South America, but is presently rare in PNG. SP resistance is associated with particular mutations in the two enzymes the drug targets, dihydrofolate reductase (DHFR) and dihydropteroate synthase (DHPS). These mutations can be analysed using molecular techniques, and the presence of 5 specific mutations (quintuple mutants in amino acids 51, 59 and 108 in DHFR and 437 and 540 in DHPS) is strongly associated with clinical failure of SP ^11^. In PNG clinical efficacy is still high (93% adequate clinical response in *P. falciparum* infections ^12^) and DHFR and DHPS quintuple mutants were not detected ^13^ SP (in combination with chloroquine) is now a key component of the PNG national malaria treatment policy. Our recent studies suggest that SP is an appropriate drug to use in PNG, but that ongoing monitoring of clinical and laboratory parameters will be necessary in determining whether effectiveness is maintained. Markers of drug resistance in the laboratory (like our DHFR and DHPS mutations) can be compared to clinical response, so that simple laboratory screening would allow us to monitor the ongoing effects of our intervention. Data from these studies will contribute to the proposed World Antimalarial Resistance Network, if this is funded. The efficacy of SP/CQ against *P. vivax* is not known, but earlier studies reported significant levels of CQ resistance in *P. vivax* isolates in PNG and neighbouring West Papua.

**Combination treatment to prevent resistance**

The impact of drug resistance to SP on its efficacy in pregnancy is not well understood ^14^ SP resistance of *P falciparum* in PNG is low grade, and the resistance profile of *P. vivax* is essentially unknown in PNG, and generally displays great geographic heterogeneity. Addition of a partner drug to SP may prevent emergence of clinically significant resistance. Evidence is now available that azithromycin (AZ) is an effective adjunctive agent for malaria treatment, in both pregnant and non-pregnant individuals, and for both *falciparum* and *vivax* infection. Data from a case management study in Malawi show azithromycin 1 g for 2 days to substantially improve cure rates over SP alone (hazard ratio for D28+ PCR-confirmed parasitological failure 0.19 (CI 0.06 to 0.63) ^15^. Data from non-pregnant adults suggest greater benefit with higher doses, of 3 g total. Azithromycin monotherapy 1 g/day for 3 days cured 89% of *P. vivax* in India ^16^, while combination azithromycin (1 g/day for 3 days) and chloroquine cured 97% of *P. falciparum* infections ^17^. Currently, one study of IPTp using SP + AZ is in progress in Malawi (Clinical Trials identifier NCT00131235) and another is planned in Tanzania (Mutabingwa, Duffy and colleagues).

**Importance of accurate speciation**

Accurate speciation of infection by microscopy is extremely hard ^18^, and is particularly difficult in pregnancy, as placental blood contains mature stages of *P. falciparum*, virtually impossible to distinguish from *P. malariae* trophozoites. We will deploy PCR-LDR analysis to accurately identify the burden of each malaria species in placental and peripheral blood ^19^. Molecular diagnosis dramatically increases sensitivity and accuracy of diagnosis of *P. vivax*, and PCR-LDR allows rapid, sensitive, specific and quantitative measure of parasitemia. This will allow us to identify morbidities associated with each species and to determine the efficacy of IPTp in a setting that is highly representative of malaria-endemic countries throughout Asia and Oceania.

**Measuring the burden of *P. vivax* in pregnancy**

In pregnancy, the consequences of *P. vivax* are still poorly understood. In Thailand, *P. vivax* had a significant negative impact on birth weight despite prompt treatment ^20^, but these findings were obtained in a population with limited malaria exposure and immunity, and have yet to be replicated. In Africa more than 90% of malaria infections are due to *P. falciparum*, but among the 1.5 billion people exposed to malaria outside Africa, *P. vivax* causes over half the disease episodes ^21^. In PNG, infection with *P. vivax* is found in around 20% of blood films in community surveys in the Wosera ^22^ and 10% in Madang. By PCR, 30% of Madang Province adults have P vivax, 20% *P. malariae* and 5-10% *P. ovale*, alone or combined with other species ^23^. In young children rates of infection are even higher. The prevalence in pregnancy is unknown, but data from Thailand suggests that it may be increased ^20^. Therefore, the results of African IPTp trials cannot be directly applied to areas with high levels of *P. vivax* malaria. There are two reasons for this. First, *P. vivax* may not respond well to SP, so women receiving SP IPT may still experience *P. vivax* related morbidity. Second, some evidence suggests that *P. vivax* may protect against *P. falciparum* disease in certain groups of patients ^22,24-26^. In pregnancy, this potential for interaction has only been studied in Thailand, where women experiencing *P. vivax* infection in early pregnancy were 52% less likely to subsequently develop *P. falciparum* malaria ^20^.

Given the dramatic impact of IPTp in Africa and the detrimental effects of malaria during pregnancy observed in PNG ^3,5^, SP IPTp could still be a highly cost effective public health intervention. It will be important to determine whether prevention of *P. vivax* will require specific prophylaxis, and whether SP+AZ adequately controls vivax in pregnant women.

**Second line treatment**

Women who are found by passive case detection to be infected with malaria will receive a treatment course of Coartem. Coartem has been adopted as the first line treatment for uncomplicated malaria in all age groups (including pregnant women in 2^nd^ and 3^rd^ trimester) in PNG.

**Effects of prophylaxis on development of pregnancy-specific immunity**

In holoendemic areas women in their first and second pregnancies are at particular risk of malaria, and immunity to placental malaria develops with increasing gravidity. If IPTp delays the acquisition of maternal immunity, this could increase the risk to mother and child during later pregnancies and thus reduce its overall effectiveness as a public health intervention. One measure of immunity is the presence of antibodies that recognize variant surface antigens (VSA) on the surface of infected erythrocytes. Antibodies to VSA expressed by placental parasites may protect against LBW and anemia ^27,28^, and their levels are highest in malaria-infected women ^29^. IPTp and ITN use might decrease or prevent the natural evolution of protective antibodies to placental VSA, and render a pregnant woman susceptible to malaria in her next pregnancy. We will determine whether IPTp in one pregnancy decreases development of immunity to pregnancy malaria in that pregnancy, or (among women who enter the study twice in successive pregnancies) in the subsequent one. Moreover, clinical and parasitological effectiveness of IPTp results from a combination of drug sensitivity and host immunity may predict the success of our intervention; conversely, our intervention might in fact impair development of host immunity. It is unclear how IPTp may affect the normal process of acquisition of immunity to malaria in pregnancy.

**Placental histology**

In African women, monocyte infiltrates in the intervillous space are common in first pregnancy, and associated with poor pregnancy outcomes ^30,31^. In our study in Malawi and a study in Tanzania, monocyte numbers were independently predictive of maternal anaemia and of low infant birth weight ^30,32^. The prevalence of placental monocyte infiltrates in areas with significant co-infection with P.vivax such as PNG is presently unknown. These events are believed to be involved in the pathogenesis of complications in pregnancy and act as markers of disease severity. The efficacy of our intervention will therefore be assessed in relation to placental pathologic changes at delivery, as well as to clinical outcomes, to gain insights into how protective benefits may occur biologically.

**Host genetics**

The populations in the study areas show a high degree of genetic adaptation to malaria, with high prevalence of red blood cell (RBC) polymorphisms, such as South-East Asian ovalocytosis (SAO), alpha-thalassaemia or the Gerbich blood group (i.e. glycophorin C deletion). Alpha-thalassaemia and SAO both protect against severe malaria in PNG ^33,34^, while glycophorin C mutations reduce invasion by P .falciparum into red blood cells ^35^. The effects of these polymorphisms on malaria in pregnancy are unknown, but they must be examined as confounders in our assessment of drug efficacy.

**Human genetics and pregnancy outcome**

In PNG, a high proportion of pregnancies may terminate in miscarriage, still birth, or birth of a premature or growth restricted baby. The genetics of both mother and baby may play important roles in determining whether mother and baby can withstand the onslaughts of infectious disease, undernutrition, and obstetric complications that are prevalent, and thus influence the likelihood of successful pregnancy outcome. Maternal survival leads to mothers who are more likely to successfully have further pregnancies, while a surviving child has the opportunity to grow and to reproduce itself. Therefore, there are strong genetic selection pressures that may operate at both levels.

**Sexually Transmitted Infections in PNG**

Sexually transmitted infections are common in Papua New Guinean women. In previous studies, prevalence of active syphilis has varied from 13.6 to 16.7 %, of chlamydia between 19.3 and 20.0 %, of *Neisseria gonorrhoea* between 10.1 and 15.1 % and Trichomonas between 17.6 and 20.0 %, between 32.1% and 35.5% having at least 1 bacterial STI. These studies were from community studies in non-pregnant, low risk women, and were not from Madang Province. Apart from their effects on the woman’s reproductive health, each of these is associated with adverse fetal outcomes including congenital syphilis, ophthalmia neonatorum, pneumonitis, prematurity and low birth weight. All of the bacterial STIs are susceptible to azithromycin treatment. Trichomonas is also common, and does not respond to azithromycin, but the evidence associating it with adverse pregnancy outcome is less compelling. PNG also has an emerging HIV epidemic. HIV seroprevalence in Madang province is around 2%, and HIV transmission is increased in the presence of genital coinfections. Thus, controlling STIs may have several benefits including improving maternal health and pregnancy outcome and decreasing the potential for HIV transmission.

**Azithromycin and STIs**

Azithromycin has excellent activity against syphilis, gonorrhoea and chlamydia. Azithromycin treatment doses to cure these infections are 2g or less (Pitsouni et al Int J Antimicrob Agents; Riedner et al NEJM 2005).

**The Malaria in Pregnancy Consortium**

The Malaria in Pregnancy Consortium, comprised of African and northern research institutions, aims to address some of these issues, and IPTp trials with different objectives are planned in African countries. However, the introduction of IPTp into non-African settings raises several critical issues that need to be addressed. In particular, non-African countries with significant malaria transmission have much higher incidences of non-falciparum malaria than most African countries and the proportion of the disease burden due to *P. vivax* is much greater. The efficacy of IPTp against *P. vivax* is currently unknown. Moreover, different parasite genetics, human genetic adaptations to malaria, and cultural differences may also influence the efficacy and appropriateness of IPTp in non-African settings. In this proposal, we will address these issues by conducting an IPTp study in Papua New Guinea (PNG). As part of this study, we will measure the burden of *P. vivax* infection in pregnancy, and will obtain maternal and child DNA samples to examine host genetic factors which determine successful pregnancy outcome.

**Summary**

**In summary, intermittent preventive therapy during pregnancy is gaining popularity as an intervention for mitigation of the devastating impact of malaria on mothers and their babies. This approach needs validation in a variety of settings, especially in areas where there is a high prevalence of non-falciparum infection. We have many years experience in studying the epidemiology of malaria in PNG and the ability to perform clinical trials such as those proposed, together with the infrastructure necessary to perform the supporting laboratory work which will demonstrate whether our intervention may have negative impact on host immunity or parasite resistance.**

We therefore propose a randomized controlled trial to evaluate the efficacy of IPTp with SP (three tablets; 1500 mg/75 mg) plus AZ (2 g daily in divided doses, i.e. 1 g twice daily for 2 days) against a treatment course of SP and chloroquine to prevent LBW in PNG women sleeping under ITNs.

## Rationale

**Pregnant women in PNG suffer large burdens of malaria and STIs, and face a growing HIV epidemic. Preventive treatment which controls both malaria and common STIs may improve pregnancy outcomes and reproductive health, and decrease susceptibility to HIV infection.**

**The Malaria in Pregnancy Consortium is moving to evaluate novel antimalarial combinations for safety and efficacy in malaria treatment and infection in many of the world’s malaria-endemic regions. Pilot data from A/Prof Rogerson and colleagues suggest that AZ added to SP may be an effective combination treatment for malaria in Malawi where SP resistance is high; in PNG, with lower SP resistance rates this combination promises to be more effective. Moreover, AZ, in doses that are needed to eradicate malaria, will also eradicate STIs such as syphilis, gonorrhoea and chlamydia. In PNG up to 40% of reproductive age women carry one of more of these infections.**

**The burden and consequences of P vivax in pregnancy are poorly understood and have been little studied. The PREGVAX initiative, led by Dr Clara Menendez, will address these knowledge gaps. This multicountry study includes PNG, as a key example of a country with high rates of endemicity and transmission of both P vivax and P falciparum. The study will contribute significantly to our understanding of the importance of P vivax in pregnancy, and will help determine whether IPTp is an effective intervention in an area where there is a high prevalence of P vivax infections, a currently unresolved question.**

**The importance of host genetics as determinants of pregnancy outcome is an unexplored area, but the revolutionary advances in technology to study the human genome has made comprehensive study of this topic possible for the first time. We will form partnerships with other organisations to study factors determining whether pregnant women have successful outcome, including maternal and child survival.**

**This proposal brings together investigators, experience, and resources to conduct a clinical trial of IPTp complemented by careful epidemiologic and laboratory investigations in a highly endemic area of Papua New Guinea, where infections with all 4 human Plasmodium species are common. The studies will be based at the PNG Institute for Medical Research, which has excellent infrastructure and a strong history of malaria research and community-based studies.**

## Potential Risks and Benefits

### Potential Risks

This study is a randomised controlled trial that will compare treatment with a full course of sulfadoxine-pyrimethamine and azithromycin (SP/AZ) at enrolment between 14-26 weeks and at two further visits at least 4 weeks apart, to a single treatment course of SP and chloroquine at first antenatal visit, and SP and AZ placebo at two subsequent visits. All women will be provided with long-lasting insecticide treated nets (ITNs). AZ has not been used extensively in PNG, but has been widely used elsewhere in developed and developing countries for treatment of STIs without evidence of adverse effects. There is a long history of use of both SP and chloroquine in pregnancy, without documented toxicity.

All drug used are usually well tolerated, however mild side effects such as nausea, diarrhoea, abdominal pain, dizziness, itching or mild rashes may occur with any of the drugs used.

Participants and their fetuses and newborn children will be monitored for the occurrence of severe adverse events, including events possibly or probably due to the study medications, by the study physicians. Details of the adverse event protocols are found in Section 9.2

In order to evaluate the effect of the drug intervention on malaria infection, haemoglobin concentrations and pregnancy outcome, blood samples will be taken at enrolment (10 ml) and at subsequent treatment visits (finger prick- 250 ml) and at delivery (10 ml). For a subset of women, an additional 5 ml venous sample (instead of a finger prick) will be taken at 28-34 weeks, for studies of immune responses to *P. vivax* in pregnancy as part of the PregVax Consortium. For a subset of 200 participating women (and 50 non-pregnant community controls), we will collect a further blood sample (10 ml) at 3 months post partum, for studies of P Vivax immunology. At delivery heel prick blood sample will also be collected from each baby. Although remote, there is a small, finite risk of infection associated with finger prick and venous blood draws. However, the PNGIMR has conducted many studies involving routine finger prick and venous blood sampling from the study populations and based on our experience from these studies, very few if any study-related adverse events, as defined below, have occurred or been reported to us.

We do not expect that participants in this study will be at significant risk of adverse consequences in the short or long term due to the study or the interventions provided. On the contrary, in large studies of SP and (separately) of AZ, severe adverse events due to the medication have been reported only extremely rarely (<1 in 10,000 persons). Moreover, both drugs have been used extensively in pregnant women in 2^nd^ and 3^rd^ trimester.

Azithromycin can induce antibiotic resistance in some bacterial species, notably S. pneumoniae (pneumococcus). Nasal swabs will be used to monitor rates of carriage, and resistance, of pneumococci.

### Known and Potential Benefits

The present study has the potential to provide both direct benefits for the study participants and general benefits for populations in malaria endemic areas in the SW Pacific and SE Asia.

For participants:

- in the SP+AZ group we expect a 30% reduction in the prevalence of LBW, and a reduction in the prevalence of malaria infection at delivery, and of severe anaemia compared to SP/CQ treatment.
- In the SP/AZ group we expect to see a reduction in the prevalence of STIs at 28-34 weeks compared to the control group.
- we will provide treated bed nets to all women, which we suspect will decrease the existing burden of malaria by around 25% on their own.

For PNG

- if the intervention proves successful, IPTp with SP +AZ is very likely to be quickly adopted. In order to facilitate such an uptake of IPTp into national treatment policies, the study will liaise closely with national and provincial health authorities.

For the wider scientific community:

- to date IPTp has not been tested in areas with high levels of P vivax malaria, or in areas outside Africa. The results of our trial will thus be highly relevant not only for PNG, but also for other countries with reasonably high levels of non-falciparum malaria.
- Combining malaria and STI interventions in one package has significant potential advantages for reproductive health programmes. Reducing the infectious disease burden in pregnancy may improve mothers’ health, decrease stillbirths and improve pregnancy outcomes,
- This study will contribute importantly to our understanding of the burden and consequences of P vivax infection in pregnancy.
- The genetics study will contribute to a greater understanding of why some pregnancies fail, and how reproductive success may be an important factor shaping the human genome.

# Objectives

**This protocol describes a randomised clinical trial of three dose administration of SP/AZ compared to single dose SP plus chloroquine and two doses of placebo to prevent low birth weight and control malaria and STIs in women using ITNS Madang Province, Papua New Guinea.**

The primary **goals** of the project are to determine whether the administration of intermittent preventive treatment (IPT) with sulphadoxine-pyrimethamine (SP) together with Azithromycin delivered through antenatal services is effective in increasing birth weight and reducing malaria parasitemia, STIs and anaemia in pregnant women. Additional goals include evaluating the burden of malaria disease due to *P. vivax*, examining the influence of IPTp on the prevalence of resistance to SP and (in S pneumoniae) to AZ, studying the human genome in the context of pregnancy outcome, and measuring the influence of IPTp on the acquisition of pregnancy-specific malarial immunity. In women who have malaria infection, we will study the characteristics of the parasites infecting them. **A benefit in our study populations would be used to support a change in PNG national policy for prevention of malaria in pregnancy, and would provide important information for other countries in the region**.

## Objectives

The **primary** **objective** of this proposal is to determine whether the administration of IPTp with SP plus AZ results in a significant decrease in the proportion of infants born with LBW. Key secondary objectives will include increases in mean birth weight and reduction in the incidence of malaria parasitemia, anaemia and sexually transmitted infections in participating women. Together, these will determine whether SP/AZ causes significant improvements in maternal and child health. This will be the first evaluation of IPTp with SP AZ outside Africa and in a setting in which *P. vivax* infection is highly endemic.

In addition, the study will investigate if the intervention may lead to an increase in levels of drug resistance; and evaluate the effect of IPTp on the development of immunity to malaria.

**Secondary objectives** of the study include:

1. To compare mean birth weight of babies born to women on SP+AZ and controls.
2. To examine the effect of SP + AZ on prevalence of *P falciparum* at delivery in peripheral, placental and cord blood films and on placental histology, and at second visit by PCR only in peripheral blood films.
3. To compare maternal haemoglobin concentrations and anaemia prevalence and severity between groups
4. To investigate the prevalence and consequences (maternal haemoglobin, birth weight and placental pathology) of *P. vivax* infection in pregnancy
5. To compare the immune response to *P. vivax*  in pregnant and non-pregnant hosts
6. To determine the incidence of symptomatic malaria by passive case detection in each group.
7. To compare the proportion of women carrying azithromycin-sensitive sexually transmitted infections at second treatment visit between groups.
8. To document the incidence and severity of Adverse Events possibly or probably associated with study medications
9. To examine the prevalence of drug resistance markers in parasites infecting women in late pregnancy
10. To measure the prevalence and antibiotic sensitivity patterns of *S. pneumoniae* in nasopharyngeal swabs collected at delivery
11. To measure the impact of IPTp on development of immunity to malaria in pregnancy
12. To document maternal, perinatal and infant mortality rates in each group.
13. To collect maternal and child DNA to study the genetics of reproductive success, as part of a planned multinational consortium.
14. To study the characteristics of parasites infecting pregnant women, such as parasite gene and protein expression and adhesion phenotypes.

The primary objective will be assessed by measuring infant birth weight at delivery and comparing the proportion of infants with birth weight <2500 g between the two groups.

**Immunological, genetic and parasitological studies related to this project will be described in separate sub-protocols.**

## Outcome measures

The efficacy of IPTp (SP/AZ) will be assessed in relation to the following indicators:

**Primary Objective**

### Primary Outcome Measure

1. Proportion of infants with birth weight <2500 g.

### Secondary Outcome Measures

- 1. Prevalence of *P falciparum* at delivery in peripheral, placental and cord blood films and on placental histology, and at second visit by PCR only in peripheral blood films.
  2. Mean maternal hemoglobin concentration at delivery, and proportion of women anaemic (Hb < 11 g/dl).
  3. Prevalence (at enrolment, second treatment, and delivery) and consequences (maternal haemoglobin, birth weight and placental pathology) of *P. vivax* infection in pregnancy
  4. Incidence of symptomatic malaria during pregnancy
  5. Proportion of women carrying azithromycin-sensitive sexually transmitted infections at second treatment visit (28-34 weeks).
  6. Incidence of Adverse Events, including severe adverse events (SAEs), and AEs possibly or probably associated with study medications
  7. Prevalence of drug resistance markers in parasites infecting women in late pregnancy, particularly in the P falciparum and P vivax dihydrofolate reductase and dihydropteroate synthase enzymes, associated with SP resistance.
  8. Prevalence and antibiotic sensitivity patterns of *S. pneumoniae* in nasopharyngeal swabs collected at delivery
  9. Maternal, perinatal and infant mortality rates.
  10. Impact of IPTp on development of immunity to malaria in pregnancy
  11. Characteristics of parasites infecting pregnant women.

# Study Design

**The study design of this research project is a randomised placebo controlled single blinded trial in pregnant women enrolled between 14 and 26 weeks’ gestation.** All women participating in this study will be followed until 6 weeks post partum. Infant survival to 1 year of age will be documented.

The intervention consists of three treatment courses of SP (3 tablets: 1500/75 mg) + AZ (2 g/day for 2 days) administered at ANC booking (provided this is between 14 and 26 weeks’ gestation) and at 2 further antenatal visits at least 4 weeks apart. Recipients in the control arm will receive a treatment course of Sp and chloroquine at enrolment, followed by AZ and SP placebos at further treatment time points. All women will be provided with a long lasting insecticide treated bed net. A block randomisation procedure will be used to allocate women to respective control and intervention groups. Among study staff, only those dispensing drug will be aware of subjects’ treatment allocation. At enrolment and at delivery a 10 ml venous blood sample will be collected to determine prevalence of malarial infections and to measure haemoglobin levels, as well as to contribute plasma, serum and peripheral blood mononuclear cells (PBMCs) for immunological analyses. At each subsequent treatment visit a 250µl finger prick blood sample will be collected for retrospective identification of malaria infection status.

At enrolment all women will have blood taken to test for syphilis by RPR and TPHA test, and women with positive tests indicating active or latent syphilis will be treated with benzathine penicillin according to standard treatment guidelines. Women will be asked to self-collect a high vaginal swab, for molecular diagnosis of STIs. Consenting women will also undergo testing for HIV (provided routinely by Modilon Hospital), and infected women will be referred to a local treatment centre providing highly active antiretroviral therapy, for prevention of mother to child transmission, and to maintain health of infected women.

From blood samples collected the prevalence and density of infections will be determined by light microscopy and PCR.

A subset of women who can attend ultrasound clinics at Modilon Hospital will have up to 3 ultrasound scans, to establish gestational age, monitor fetal growth, and measure placental blood flow.

At other regular antenatal care visits women will not be actively followed for morbidity. However, women self reporting febrile illness will be immediately diagnosed by study staff and if required treated for malaria.

At the time of scheduled final treatment dose, women will have a high vaginal swab collected, and all women with symptoms of STIs (or a positive diagnosis by PCR) will be treated according to PNG Standard Treatment Guidelines for Syndromic Management of STIs.

During the entire study period a passive case detection system will be maintained at the participating hospital and health centres (HC) to determine the incidence of symptomatic malaria illness. Every participating woman who presents with a febrile illness or symptomatic anaemia will be clinically assessed and have venous blood (5 ml) taken for the determination of malaria infection (by Rapid Test, confirmed by microscopy and PCR) and Hb level.

The use of a relatively broad spectrum antibiotic for the prevention of malaria and STIs in pregnancy, raises the concern that this intervention my increase resistance in other important bacterial pathogens. Even though the dose given is significantly higher than that recommended for treatment of bacterial illnesses, we will be monitoring resistance level in pneumococci collect from nasal mucosa of a subset of women delivery. Pneumococci were selected as carriage rates are high, and they are easily collected and cultured.

Each participant will thus have up to 4 regular study contacts during gestation and 2 after delivery (6 weeks and 12 months) and will be asked to contribute 2 x 10 ml venous blood samples and 1 or 2 finger prick blood samples (250µl) (See Study Schedule, Appendix I). Additional blood samples will be collected at time of febrile illness during passive case detection.

The safety of all study participants will be assured by a specifically constituted data & safety monitoring board (DSMB), while the study integrity will be guaranteed by regular visits by an external clinical monitor.

# Study Population

## Selection of the Study Population

In order to be able to enrol 2792 pregnant women in 18 months (and to limit numbers of multigravid women to equivalence with primigravid women) we will need to recruit women at multiple sites. The studies will therefore be conducted at several Health Centres and the Provincial Hospital in Madang Province.

Modilon Hospital has approximately 1100 ANC bookings, and >1500 deliveries, each year. Clinics in Madang town see around 700 new patients each year. Mugil and Alexishafen Health Centres each have 500-600 ANC bookings, and Yagaum Health Centre has 250 deliveries annually. Average parity is approximately 5; we estimate that 50% of ANC attendees will be multigravidae and 20% primigravidae, and therefore about 70% of women will be eligible for the study. Of >4000 potential enrolees, we plan to enrol approximately 65%.

Women are eligible for participation in this study if they fulfil the following criteria:

- - permanent resident in study area
  - exclusive use of study health facilities for primary health care
  - primigravid, secundigravid, or multigravid women; the latter will be enrolled in a 1:1 ratio with primigravid women.
  - Age is between 16 and 49 years

For studies of the effects of pregnancy on P viva immunity, we will re-consent a subset of 250 consecutive women at delivery, for a further visit and blood collection at 3 months post partum.

50 non-pregnant controls will also be recruited from communities from which participating women are drawn.

## Inclusion/Exclusion Criteria

Upon informed consent all eligible participants (see 5.1 above for criteria) will be screened for the following exclusion criteria:

- Known chronic illness, e.g. TB, diabetes, renal failure
- Severe anaemia requiring hospitalisation (Hb < 6 g/dl accompanied by symptoms requiring urgent treatment), or
- permanent disability, that prevents or impedes study participation and/or comprehension
- Known multiple pregnancy

Any one or more of the criteria is sufficient to exclude study participation. Women with severe anaemia (Hb <6 g/dl) but without significant symptoms can be enrolled. Anaemia will be treated according to national standard treatment guidelines. All women excluded on health grounds will be referred to Modilon Hospital, where they will be treated according to PNG national treatment guidelines.

# Study Procedures/Evaluations

## Study Procedures

This study’s primary goal is to conduct a randomised controlled trial of intermittent preventive treatment with SP+ AZ (IPTp), compared to single dose curative treatment with SP plus chloroquine in pregnant women using ITNs, delivered through ANCs. The main study outcomes will be evaluated at delivery. Following the intervention women and their children will be followed at 6 weeks and 12 months to measure maternal, neonatal and infant mortality rates. During the antenatal period, women will be followed with passive case detection at participating health facilities.

An overview of the study procedures is provided below and discussed in greater detail in the following subsections.

At first ANC attendance, pregnant women estimated to be between 14 and 26 weeks’ gestation will be invited to join the study. Women presenting in the first trimester ≤13 gestation weeks will be eligible to enter the study and will have blood taken and ultrasound scan performed, but they will be asked to return to receive their initial treatment. Gestation at enrolment will be assessed by ultrasound (when available; primarily at Modilon Hospital), and in its absence by a combination of LMP history from women and abdominal palpation.

At first treatment visit, after informed consent has been obtained, women will be assigned a unique PNGIMR identification number. For each unique identification number, a prepared opaque envelope containing the treatment assignation of that women willl be opened to determine which treatment group she belongs to. Following enrolment a venous blood sample (10 ml) will be collected and the woman will receive the first IPTp treatment. Participants will be provided with a free, long-lasting ITN. Thereafter, the study team will see the woman on one or two further occasions during pregnancy. For women enrolled at or before 26 weeks gestation, they will be seen at two further visits at least 4 weeks apart. At each visit the allocated treatment (SP + AZ at all visits, or SP + CQ at first visit, SP and AZ placebos at subsequent visits) will be dispensed. Only women who spontaneously report illness will undergo an in-depth physical examination. At delivery the mother and her newborn baby will undergo an in-depth clinical examination (including Hb measurement) and a 10 ml venous blood sample will be collected for immunological investigations from mother and from cord blood.

After enrolment, women will attend ANC according to PNG standard recommendations, and their care will be provided by health centre or hospital staff. Women attending for second visit and final visit will receive a further fingerprick blood test, and a further treatment dose. At delivery, a further 10 ml venous blood sample will be taken, either during labour, or within 24 h of delivery. Placental samples and cord blood (from the placental side of the cut cord) for histology, preparation of blood smears and/or immunological studies will be collected. The follow-up of the women will cease at 6 weeks post partum, and of the babies at 12 months post partum. The exception to this is the follow up of 250 women to 3 months post partum for studies of P vivax immunity Schematic representation of study schedules and activities is given in the Study Schedule Diagram in Appendix I

During the entire study period the study will maintain a passive case detection system at participating HCs to detect and treat febrile illness episodes of study women. Active case detection will not be performed.

Ultrasound examinations will be performed on as many women as possible, using a machine based at Modilon Hospital. Standard gestational assessments (biparietal diameter, head and abdominal circumference, femur length) will be performed. At subsequent visits, fetal growth monitoring will be performed, and Doppler flow wave velocimetry (to measure placental blood flow) will be undertaken.

### Field Study Team

The study will be coordinated and day-to-day activities will be carried out by PNGIMR field study teams based at the ANC clinics. The total team is comprised of approximately 21 individuals who live and work in or near the study area. It consists of three study physicians (one expatriate senior physician/field manager, two PNG national), a minimum of 14 nursing officers, 2 microscopists and a laboratory technician. The study physicians and nursing officers are trained specifically to conduct information meetings (“tok saves” in Melanesian Pidgin), recruit participants, and organize and conduct the collection of blood and other measurements based on the research collaborators’ study protocols. Besides organising and supervising the field teams the study physicians are responsible for assuring adequate clinical care of study participants. They will have received appropriate human subjects protections and study conduct training to conduct the study according to good clinical practice (GCP) guidelines. Field study team members routinely work on large-scale malaria research studies that include the collection of blood for archives and laboratory analyses, making blood smears, obtaining haemoglobin concentrations, interviewing study participants, and explaining the results of blood smear and haemoglobin evaluations.

The study team will be supported by a network of village reporters that help the study team communicate with participating villages, assist in follow up of women and children after delivery and encourage women who miss their treatment or follow-up appointments to attend the health centre to make up for the missed visit. Village reporters will also have an important role in detecting out-of-facility deliveries, and ensuring children are weighed within the first 7 days of life.

## Participant Database and Study Visit Activities

After enrolment, the woman’s baseline information will be entered into a participants’ database that will provide scheduling information for subsequent study contacts. As the study will enrol women for 18 months, the study will have an open cohort. The participants’ database will be updated monthly to reflect the status of the cohort through indicators that distinguish women under active follow-up versus women who have delivered (reaching primary study endpoint), and women with completed follow-up or censored from follow-up. The participant database will serve to schedule treatment and follow-up visits for the study participants.

### First contact and pre-screening

The study team will contact potentially eligible women at first ANC visit. The objectives and procedures of the study will be explained and women will be invited to join the study if they are between 14 and 26 weeks pregnant (in which case they will be eligible for treatment that day), or if they are ≤13 weeks pregnant (in which case they will be enrolled, have a finger prick blood sample taken for determination of malarial infections and requested to come back when they reach 14 weeks gestation). Details of eligible women who are in first trimester will be kept in a pre-screening database to ensure they commence treatment at next scheduled visit.

Formal, written informed consent will be obtained at first ANC visit.

### Enrolment and Treatment

1. **Enrolment and 1^st^ Treatment Visit**

Baseline data collection and 1^st^ IPTp treatment will take place at first ANC visit; the exception to this is women in first trimester who will be enrolled at first visit but will only receive first IPTp treatment when they enter second trimester. After consent is obtained, the PNGIMR field study team will conduct the following tasks:

Woman:

- Assess age
- Assess gravidity and parity
- Take anthropometric measurements
  - Weight
  - Height
  - MUAC
- Collect venous blood sample (approximately 10 ml)
  - Prepare thin and thick blood smears
  - Test blood for serological evidence of active syphilis infection by card based RPR test.
  - Archive a portion of the blood sample for laboratory analyses
  - Measure Hb using Hemacue
- Assess for symptoms of severe anaemia, chronic illness or permanent disabilities
- Whenever possible, collect a self-administered high vaginal swab for determination of sexually transmitted infections (STIs)
- Interview and record
  - demographic information (including participant’s date of birth)
  - history of woman’s bednet use in two weeks prior
  - woman’s history of malaria treatment in two weeks prior
- Check the woman’s health records booklet for recent health centre visits and record applicable information
- In women without available date of birth, the age of the woman will be estimated using a calendar of notable local events.
- Book appointment to perform transabdominal ultrasound to measure biparietal diameter, femoral length and abdominal circumference (to estimate gestational age). In women who are ≥24 weeks’ gestation, Doppler studies will be performed, to measure uteroplacental blood flow, and to seek abnormal flow velocity waveforms in the uteroplacental arteries. We expect that this will be performed on all women at Modilon Hospital, but not at all the Health Centres due to logistic constraints.

Data and responses of the woman will be recorded on Enrolment case report forms (CRF) at the time the measurements and interview are conducted. The participant’s health records booklet (if any) will be checked to see if any visits to a health centre have been documented. If the participant has any record of a visit, the field study team will record the date of the most recent visit and any medication the participant received on the Enrolment Form. In women without a health record booklet, a new booklet will be provided free of charge by study staff.

The study team Physician or a trained study team Nursing Officer will assess the woman for sign of chronic illness, symptomatic severe anaemia or permanent disabilities that would preclude her participation into the study. (See 5.2 for list of exclusion criteria). If a woman is excluded on health grounds, she will be referred to nearest health facility for treatment.

Every enrolled woman’s health (record) booklet (already owned, or provided at enrolment) will be clearly marked with a study identifier and study ID number. At any study contact, the study team will record all medically relevant data collected during their examination into the health booklet.

Digital photographs will be taken of participating women at enrolment, and a printed version will be kept in a secure file, for tracking of pregnant women who fail to attend appointments, and to ensure accurate identification of women if required

Following assessment the woman will receive the 1^st^ IPTp treatment dose. The drugs will be dispensed in pre-randomised and pre-numbered Ziploc bags that contain all drugs for initial treatment. The first dose (SP and AZ or SP and chloroquine) will be dispensed directly by the study staff. Pack number, date and time of administration will be recorded on the enrolment forms. The tablets will be administered with water. The woman will then be instructed how to take subsequent AZ or chloroquine doses at home. The woman’s treatment allocation will be noted using randomly-generated codes. Treatment allocation will not be revealed to laboratory staff, and will be kept concealed to the maximum extent possible from other study staff (see 6.3.3.). Senior study investigators will be aware of treatment allocation; hence the study will be single blinded.

A thorough physical examination (including axillary temperature, measurement of Hb etc. will only be conducted on enrolled women who spontaneously report as ill to either the study team or the health centre staff. Women with a presumptive diagnosis of malaria will be tested on the spot for malaria infection using a rapid diagnostic test (RDT). If they have a positive RDT they will be treated with a full course of Coartem and their randomised IPTp treatment (drug or placebo) will be delayed for at least 4 weeks. For all women reported sick, detailed symptomatology, results of RDT and treatments given will be recorded on the ‘Morbidity Surveillance Form’.

At the end of the enrolment visit the study team will schedule the next treatment visit with the woman. Participants will be instructed to seek medical attention immediately if they have any sign or symptoms of illness or any other condition that they deem as a threat to their well-being following the treatment. They will also be encouraged to seek medical care if they note any symptoms of malaria, fever, or illness, and to report any antimalarial treatments received or used.

1. 2^nd^ Treatment Visit

During the 2^nd^ treatment visit the PNGIMR study team will conduct the following tasks:

Woman:

- Interview and record
  - history of bednet use in two weeks prior
  - history of malaria treatment in two weeks prior
- Take growth measurements
  - Maternal weight
  - Fundal height
- Collect 250 µl finger prick blood sample
  - Prepare thin and thick blood smears
  - Archive a portion of the blood sample for laboratory analyses
- Check the woman’s health records booklet for recent health centre visits and record applicable information
- Measure maternal blood pressure and check for oedema. If elevated blood pressure or oedema present, women will be tested for proteinuria. Presence of all three will be considered clinical evidence of pre-eclampsia, and women will be referred for obstetric care through Modilon Hospital.
- If possible, perform second ultrasound examination for fetal growth monitoring.

Data and responses of the participant will be recorded on the 2^nd^ Treatment Visit CRF at the time the measurements and interview are conducted. As at enrolment a thorough physical examination (including axillary temperature, measurement of Hb, etc.) will only be conducted on enrolled women who spontaneously report as ill to either the study team or the health centre staff conducting the clinic. Women diagnosed with presumptive malaria will be tested on the spot for malaria infections using a rapid diagnostic test (RDT). If the RDT is positive, they will be treated with a full course of Coartem instead of the scheduled treatment. For all women reported sick, detailed symptomatology, result of RDT and treatments given will be recorded on the ‘Morbidity Surveillance Form’.

Following the collection of data and blood sample women receiving SP + AZ will receive the 2^nd^ IPTp treatment dose, women in the control arm will receive SP and AZ placebos. The first dose will be dispensed directly by the study staff. Pack number, date and time of administration will be recorded on the enrolment forms. The participant will then be instructed how to take the 2^nd^ dose at home.

At the end of the visit the field study team will schedule the next treatment visit with the woman. Women who failed to attend the study ANC clinic will be contacted by the village reporters and encouraged to present at their health centre to receive the missed treatment.

1. Third IPTp Treatment Visit

During the final treatment visit the PNGIMR field study team will conduct the following tasks:

Woman:

- Interview and record
  - history of bednet use in two weeks prior
  - history of malaria treatment in two weeks prior
- Take growth measurements
  - Maternal weight
  - Fundal height
- Collect 250 µl finger prick blood sample (in first 150 women, collect instead 5 ml venous sample for plasma separation and immunological studies).
  - Prepare thin and thick blood smears
  - Archive a portion of the blood sample for laboratory analyses
- Where possible, collect a self-administered high vaginal swab for determination of sexually transmitted infections (STIs)
- Check the woman’s health records booklet for recent health centre visits and record applicable information
- Measure maternal blood pressure and check for oedema. If elevated blood pressure or oedema present, women will be tested for proteinuria. Presence of all three will be considered clinical evidence of pre-eclampsia, and women will be referred for obstetric care through Modilon Hospital.
- Ultrasound will be performed where available to (a) measure fetal growth using bi-parietal diameter, head circumference, abdominal circumference and femur length and (b) document uteroplacental blood flow patterns using color Doppler, as described

Data and responses of the participant will be recorded on the Final Treatment Visit CRF at the time the measurements and interview are conducted. As at enrolment a thorough physical examination (including axillary temperature, measurement of Hb, etc.) will only be conducted on enrolled women who spontaneously report as ill to either the study team or the health centre staff conducting the clinic. Women diagnosed with presumptive malaria will be tested on the spot for malaria infections using a rapid diagnostic test (RDT). If the RDT is positive, they will be treated according to PNG standard treatment guidelines with a full course of Coartem instead of the scheduled treatment. For all women reported sick, detailed symptomatology, result of RDT and treatments given will be recorded on the ‘Morbidity Surveillance Form’.

Following the collection of data and blood sample the woman will receive the Final IPTp treatment dose (if she is allocated to the SP and azithro arm of the study, or matching SP and Az placebo). Administration will be as for the second treatment dose.

At the end of the visit the field study team will remind the woman of the importance of delivering in the Health Centre or Hospital. Women who fail to attend the study ANC clinic will be contacted by the village reporters and encouraged to present at their local health centre to receive the missed treatment.

### Delivery and Assessment of IPTp intervention

The primary assessment of the IPTp intervention will take place at delivery.

The specific tasks to be completed by the field study team at the delivery visit are as follows:

Baby:

- Record pregnancy outcome (i.e. live birth, stillbirth, abortion)
- Record birth weight
- Collect a heel prick blood sample (200 µl)
  - Prepare thin and thick blood smears
  - Archive a portion of the blood sample for laboratory analyses
  - Measure Hb using Hemacue
- Collect a cord blood sample (approximately 10 ml)
  - Cord haemoglobin concentration
  - Archive a portion of the blood sample for laboratory analyses

Mother

- Interview and record
  - history of bednet use since previous visit
  - history of malaria treatment since previous visit
- Collect maternal peripheral blood (10 ml)
  - Prepare thin and thick blood smears
  - Measure Hb using Hemacue
  - Archive a portion of the blood sample for laboratory analyses
- Collect placental blood films and placental biopsies
- Collect a per-nasal swab

Check the health records booklet for recent health centre visits and record applicable information.

Mothers that deliver at home will be asked to visit Modilon Hospital or a participating health center within no more than a week. In these cases the following tasks will be performed:

Baby:

- Record day of birth
- Record pregnancy outcome (i.e. live birth, stillbirth, abortion)
- Record weight
- Collect a heel prick blood sample (200 µl)
  - Prepare thin and thick blood smears
  - Archive a portion of the blood sample for laboratory analyses
  - Measure Hb using Hemacue

Mother

- Interview and record
  - history of bednet use since previous visit
  - history of malaria treatment since previous visit
- Collect maternal peripheral blood (10 ml)
  - Prepare thin and thick blood smears
  - Measure Hb using Hemacue
  - Archive a portion of the blood sample for laboratory analyses

Check the health records booklet for recent health centre visits and record applicable information.

Before discharge, the field study team will schedule a 6 week post partum visit with the woman and her baby.

### Long-term follow-up following intervention

1. Post partum visit (6 weeks)

This visit will take place concurrent with Health Centre EPI clinics. A week before the scheduled date for the visit, village reporters will visit participating women and their babies, and explain the importance of attending the assessment visit and remind them of time and location of the clinic.

The specific tasks to be completed by the field study team during the Follow-up Visits are as follows:

Child:

- Take health indicators
  - Weight
- Collect heel prick blood sample (approximately 200-250 μl)
  - Prepare thin and thick blood smears
  - Archive a portion of the blood sample for laboratory analyses

Mother of child:

- Interview mother and record
  - history of bed net use since previous visit
  - history of malaria treatment for mother or child since previous visit
- Check the child’s health records booklet for recent health centre visits and record applicable information
- Record maternal weight

Data and responses will be recorded at the time the measurements and interview are conducted on the Follow-up CRF. The participant’s health records booklet will be checked to see if any visits to a health centre have been documented. If the participant has any record of a visit, the field study team will record the date of the most recent visit and any medication the participant received on the CRF.

Women who fail to attend this Visit will be followed-up at their homes by the study team. If found the study team will conduct examination and blood collections at the family’s home.

1. 3 month post partum follow up, selected women

We enrol 250 consecutive participating women into a sub-study of effects of pregnancy on P vivax immunity. Women will attend on one further occasion, 3 months post partum. Tasks to be completed at this visit include:

- Interview mother and record
  - history of bed net use since previous visit
  - history of malaria treatment for mother or child since previous visit
- Check the child’s health records booklet for recent health centre visits and record applicable information
- Record maternal weight
- Collect 10 ml venous sample from mother

1. Final Evaluation Visit (infants only, 12 months of age)

The final assessment of the IPTp intervention will take place at 12 months of age, concurrent with normal EPI clinics. A week before the scheduled date for the visit, village reporters will visit the parents of participating children, explain the importance of attending the assessment visit and remind them of time and location of the next clinic.

The specific tasks to be completed by the field study team during the Final Evaluation Visit are as follows:

Child:

- Take health indicators
  - Weight
  - Measure spleen size
- Collect a finger prick blood sample.
  - Prepare thin and thick blood smears
  - Obtain haemoglobin concentration
  - Archive a portion of the blood sample for laboratory analyses
- Determine if child is suffering from symptomatic malaria or moderate-to-severe anaemia (Hb < 8.0 g/dl)

Mother of child:

- Interview mother and record
  - history of child’s bednet use since previous visit
- Check the child’s health records booklet for recent health centre visits and record applicable information
- Record maternal weight

Data and responses of the participant will be recorded at the time the measurements and interview are conducted on the Final Evaluation CRF.

### Passive Surveillance

1. Health Centre Readiness and Participation

All study villages are situated within areas serviced by the Catholic Health Services through their health centres in Alexishafen and Mugil (Madang North Coast), by Modilon Hospital and nearby Health Centres (Town, Jumba and Sisiak Clinics) (Madang Town) and Yagaum Health Centre (Lutheran Health Services). The health centres outside Madang Town have both in-patient and out-patient facilities and are staffed with a health extension officer and several nurses and community health workers. The health centre staff routinely assess pregnant women for malaria and other intercurrent illnesses. Tertiary referral services are provided by Madang Provincial Hospital

As at all other PNG health centres attendance and admittance log books are kept. Each health centre in the province has a HF radio and radio communication protocols are established for reporting all severe health problems that require further medical support. This radio serves as a communication to the referral hospital in Madang.

The primary investigator and the field study team will meet with the health centre staff prior to initiation of the study to explain the study’s objectives and conduct and review the involvement of the health centres in the study. The field study team will coordinate with the health centre nursing staff for setting up both joint study ANC clinics and a morbidity surveillance system

For the purpose of morbidity surveillance, the PNGIMR maintains a study clinic within the larger health centres and in the Hospital during all out patient clinic hours. The study clinics are staffed with (at least) 1 nursing officer and rural laboratory assistant trained in diagnostic malarial microscopy. Participants will be encouraged to preferably or exclusively visit the participating health centres.

The health centre staff will be requested to screen pregnant women who are attending ANC or outpatient clinics in order to determine if they are study participants. Screening can be accomplished by checking for study symbol and ID numbers on the woman’s Helt Buk (Health Book) or by referring to the list of study participants that will be kept at the study clinics. In addition, women who are participating in the study will be requested to aid the health centres and study team by alerting their health care providers that they are study participants when independently seeking care at one of the participating health centres. After initial diagnosis health centre staff will refer study participants to the IMR morbidity surveillance staff for further clinical investigations and data collections.

1. Diagnosis & Treatment

All participants identified by health centre staff will be referred to the health centre based study nurse. The study nurse will take a medical history and fill in the Health Centre Surveillance form. In the case of participants diagnosed with presumptive malaria, s/he will take a finger prick blood sample to make blood smears, measure haemoglobin and confirm the presumptive diagnosis using on site with a rapid diagnostic test (RDT) or diagnostic microscopy.

Upon confirmation of diagnosis of malaria illness, the participant will be treated by the study nurse with antimalarials. All symptomatic episodes will be treated with the rescue medication (i.e. 3 day course of Coartem). In the case of a haemoglobin < 6.0 g/dl study staff will treat the woman according to PNG national guidelines with a full course of antimalarials, albendazole for hookworm control and iron infusion.

For women with negative RDT/bloodslide and/or Hb ≥6.0 g/dl the health centre staff will be advised to refine the diagnosis for other febrile illnesses and treat accordingly. All women with a diagnosis other than malaria and anaemia will be treated by the health centre /hospital staff.

The study surveillance will only be maintained during official outpatient clinic hours. For women presenting after hours, the health centre staff will be taught how to fill in a simplified After-Hours Surveillance Form, take blood slides, finger prick blood samples and conduct malaria RDTs. The health centre staff will be encouraged to only treat women with antimalarials if the RDT result is positive. The following morning the study surveillance staff will collect blood samples and blood slides from the health centre staff and review forms.

INTERACTIONS WITH AID POSTS Workers at local aid posts will be instructed to refer study women to the nearest health centre for diagnosis and treatment.

1. Health Centre Surveillance Records

The PNGIMR field study team will coordinate with the health centres for the collection and, if necessary, abstraction of specific health information on study participants. Data from the Health Centre Surveillance Forms also are entered into a Health Centre Surveillance Database at regular intervals and maintained by the PNGIMR.

1. Health Records Booklets

All pregnant mothers attending ANC clinics are required to purchase and carry a health records booklet when seeking medical care at health centres. This health records booklet provides information as to the pregnancy as well as illnesses and treatments an individual has had during pregnancy. Additionally, the health records booklets for participants in IMR research studies are marked with a brightly colored sticker.

At enrolment we will thus provide a standard health booklet to each study participant, if she does not have one, free of charge. At delivery of the newborn, we will also provide the mother with a health booklet for her baby free of charge. Each of the health records booklets will be marked with a brightly coloured sticker denoting participation in this study and their study ID number. The sticker number alerts health care providers at the health centres that the individual is participating in the IPTp study, while the ID number allows easy identification of the participant by the surveillance staff.

The field study team will review each participant’s health records booklet at the regular scheduled study visits, record all health care visits on the respective forms.

### Treatment of Sexually Transmitted Infections

All women diagnosed with symptoms of STIs at any treatment contact will be treated according to PNG Standard Treatment Guidelines for Syndromic Management of STIs, irrespective to treatment allocation.

At enrolment all women will have blood taken to test for syphilis by RPR and TPHA test, and women with positive tests indicating active or latent syphilis will be treated with benzathine penicillin according to standard treatment guidelines.

Self-collected high vaginal swabs will be sent for molecular diagnosis of STIs at the IMR in Goroka. Positive test results will be communicated to the participating women by specially trained staff that will provide counselling to women (and their partners). Women in the control group (as well as partners of all women) will receive treatment according to PNG Standard Treatment Guidelines for Syndromic Management of STIs. As azithromycin in dose given is highly effective in treating all bacterial STIs, women in the intervention group will only receive additional treatment if they are positive for trichomonas.

Consenting women will also undergo testing for HIV (provided routinely by Modilon Hospital), and infected women will be referred to a local treatment centre providing highly active antiretroviral therapy, for prevention of mother to child transmission, and to maintain health of infected women.

## Laboratory Evaluations

### Laboratory Evaluations/Assays

**6.2.1.1. Analyses to be performed at the PNGIMR Laboratories in Madang**

1. **Diagnosis of Malaria Infection by Blood Smear**

Thick (2) and thin (2) smears will be prepared on each participant in the study at every study visit and on an as needed basis during morbidity surveillance by PNGIMR staff who have extensive experience in field preparation of blood smears. Evaluation of the blood smear is performed by examining a total number of oil immersion fields to include 200 leukocytes, with the assumption that mean leukocyte count is 8000/μl blood. Results are expressed as the number of asexual parasites per μl blood. These methods will be described in the Field and Laboratory Procedures Manual, to be submitted separately.

1. **Blood Sample Processing**

All finger prick blood samples collected during study visits and morbidity surveillance will be separated into plasma and cells and frozen and stored separately. Blood from venous blood draws will be separated into plasma, WBC and RBC fractions using standard protocols. All fractions will be aliquoted and frozen or cryopreserved as required.

From venous and cord blood samples, peripheral blood mononuclear cells (PBMCs) will be extracted after separation of plasma by centrifugation. Cell pellets will be passed over Percoll and the PBMC layer washed, counted and cryopreserved. They will be stored at -80C before transfer to liquid nitrogen. Plasma and remaining cell pellets will be aliquoted for laboratory studies. Detailed procedures for sample separation, storage are given in the immunology sub-protocol and in the relevant SOPs

Detailed procedures for sample separation, storage are given in the immunology sub-protocol and in the relevant SOPs

1. **Genital & Per-Nasal Swabs**

High vaginal swabs (self-collected by study participants) will be obtained and placed into transport medium. Swabs will be stored at -20C and transferred to the IMR’s Goroka laboratory for processing including DNA extraction (6.2.1.2) and PCR as described in the laboratory sub-protocol

Per-Nasal swaps will soaked in the skim milk glucose glycerol broth and stored away at -20^0^ until transport to Goroka microbiology lab.

1. **Host genetic analyses**

In order to obtain high-quality DNA for genomic analysis, individual samples of maternal and infant blood will be extracted using protocols established by MalariaGEN (a malaria genomics study, currently in progress in Madang).

This DNA will be used for host genotyping including common red cell abnormalities like glycophorin C, south east asian ovalocytosis and alpha thalassemia and other know genetic polymorphisms that may alter the risk of malaria.

Results of genetic testing are for research purposes only and will not be passed on the participants.

**6.2.1.2. Analyses to be performed at PNGIMR Laboratories in Goroka**

1. **DNA extraction and diagnosis of Malaria Infection by Polymerase Chain Reaction (PCR) / Ligase Detection Reaction (LDR)**

DNA extraction will be performed using the QIAmp 96 blood extraction protocol (QIAGEN). This approach enables DNA extraction from 192 samples in 2-3 hours. Using blood samples, it has been used successfully to PCR amplify both human- and malaria parasite-specific amplicons for genotypic and species specific diagnostic assays, respectively. Because DNA extraction methodologies are based upon starting with whole blood, preliminary protease treatment will lyse leukocytes, erythrocytes and Plasmodium parasites. Therefore, purified genomic DNA contains a mixture of human and parasite DNA. The protocol for DNA extraction will be in accordance with the procedures set forth in the Field and Laboratory Procedures Manual.

As each individual’s genomic DNA preparation contains human and parasite DNA, blood samples can be used directly to perform species-specific PCR/LDR-based diagnosis of Plasmodium infection . The protocol for the PCR/LDR-based diagnosis of malaria infection will be in accordance with the procedures set forth in the Field and Laboratory Procedures Manual.

**Typing of Molecular Markers of SP resistance by Polymerase Chain Reaction (PCR) / Ligase Detection Reaction (LDR)**

A random sample of PCR positive infection detected at last treatment time point and delivery will be assess for the presence of know markers of SP resistance in the Pf*dhfr*, *Pfdhps* and *Pvdhfr* genes using established PCR/LDR-based assays.

1. **Diagnosis of sexually transmitted infections by Polymerase Chain Reaction (PCR)**

DNA will be extract directly for self-collected vaginal swabs using established protocol. The thus obtained DNA samples will be assessed for the presence of *N gonorrhoeae*, *C trachomatis* and *T vaginalis* using an established multiplex PCR. The protocol for DNA extraction and PCR-typing will be in accordance with the procedures set forth in the Field and Laboratory Procedures Manual.

1. **Determination of Pneumoccocal Carriage and Drug resistance**

The use of a relatively broad spectrum antibiotic for the prevention of malaria and STIs in pregnancy raised the concern that this intervention may increase resistance in other important bacterial pathogens. As the dose given for malaria control is significantly higher than that recommended for treatment of bacterial illnesses, this is very unlikely. Nevertheless we will assess if IPTp with AZ-SP does increase the risk of antibiotic resistance to AZ, by monitoring resistance levels in pneumococci collect from nasal mucosa for participating women. Pneumococci were selected as carriage rates are high, they are easily collected and cultured.

Per-nasal swab specimens will be cultured and *S. pneumoniae* isolates identified following standard procedures. Resistance to Azithromycin will be assessed by determination of MICs using strips from AB BioDisk International. Detailed procedures will be set forth in Laboratory Procedures Manual.

### Special Assays or Procedures

All drug used for the study intervention will be procured from manufacturers that can guarantee a GMP quality product and independent quality control of these drugs will be conducted. The Azithromycin will be procured from Pfizer. GMP quality SP and chloroquine has been obtained from Farmamundi, Spain.

### Specimen Collection, Preparation, Handling and Shipping

#### Instructions for Specimen Preparation, Handling, and Storage

1. **Blood Collection**
   1. Finger prick bleeds

At the second and third (where applicable) treatment dose blood samples of approximately 250 μl will be collected from each woman participating in the Protocol. Additional samples will be taken from symptomatic individuals during passive morbidity surveillance. These blood samples will be obtained by the finger prick method.

These samples will provide adequate blood for the preparation of thin/thick blood smears to detect the presence of malaria parasites, type of malaria parasites, and level of parasitemia. A portion of the sample also will be archived in a K^+^EDTA microtainer for laboratory analyses (e.g. serum & DNA preparation, PCR/LDR-based diagnosis of malaria parasites).

- 1. Venous blood draws

In order to have sufficiently large samples for the detailed immunological measurements detailed in the supplementary protocol, venous blood draws are needed at enrolment and delivery. The volumes of the venous draws are limited to 10 ml. Cell mediated immunology will be studied in separately funded studies.

Drawing venous blood samples will be done by specially trained staff. We will use butterfly needles of 21 gauge and heparinized vacutainers. Residual blood in the tube attached to the butterfly needle will be used to measure haemoglobin levels and make blood smears.

Venepunctures will be performed on veins in the participants’ arms. Upon removal of the needled localized bleeding is efficiently stopped with a small pressure bandage (e.g. a Band Aid). Properly done this procedure is virtually painless. However, if the needle punctures the blood vessel, there is a risk of local bleeding that can result in minor hematoma. In order to assure the safety of participants, they will be monitored following blood draw until it can be ascertained the local bleeding has stopped. Subjects will be instructed to present to the study nurses based at participating health centres in case of localized hematomas or infection at site of needle injection who will then attend to them and if necessary refer them for further treatment.

These samples will provide adequate blood for the preparation of thin/thick blood smears, direct measurement of haemoglobin (enrolment and delivery only), DNA extraction for parasite and host genetic typing as well as provide peripheral blood mononuclear cells (PBMC’s) and parasites for in-depth immunology studies. The sample will be collected in heparinized tubes and separated into red cell, white cell and serum fractions. Samples of these fractions may be stored frozen for later laboratory analyses.

1. **Preparation of Blood Smears**

The preparation of thin and thick blood smears will be in accordance with the procedures set forth in the Field and Laboratory Procedures Manual.

1. **Determining Haemoglobin Concentration**

At enrolment and delivery, haemoglobin concentration will be determined directly in the field by using a HemoCue® device (HemoCue, Ångholm, Sweden) and will be expressed as grams of haemoglobin per decilitre. The instrument records haemoglobin concentrations to the nearest tenth of a gram per decilitre.

#### Specimen Shipment

At regular intervals, blood smears, and blood samples will be transported from the hospital and health centres to the PNGIMR’s Madang laboratories as well as between the Madang and Goroka laboratories. Delivery and receipt of blood smears, and blood samples will be confirmed by both e-mail and telephone and recorded in sample shipment log books. Details for storing and shipping blood smears and blood samples are outlined in the Field and Laboratory Procedures Manual.

DNA aliquots, serum and cryopreserved PBMC and parasites may be shipped to the WEHI or UoM laboratories in Melbourne, Australia, to the Hospital Clinic in Barcelona, or CGHD, Cleveland, Ohio for in-depth laboratory analyses that can not be conducted at the PNGIMR laboratories in Madang or Goroka. This shipment will be done using Air Nuigini freight or specialized dangerous goods couriers. All shipments by air will comply with IATA rules for shipment of diagnostic specimens.

## Randomisation and Blinding

### Randomisation procedures

Women will be randomized to receive SP/AZ or SP/CQ using concealed allocation. Each treatment will be randomly assigned to 4 different treatment codes, resulting in a total of 8 treatment codes (i.e. A, B, C, D, E, F, G, H). A block randomization procedure will be used, with blocks of 32, each containing 4 women assigned to each treatment code. This will help maintain concealment of allocation from study staff to the greatest extent possible.

In order to facilitate field operations independent randomization lists will be produced for each health centre / hospital. The resulting list will contain 1500 women for Modilon Hospital, 500 for each of the peripheral clinics in Madang Town, 500 each for the two field teams operating in the Madang North Coast (Mugil & Alexishafen HC) and 500 for Yagaum Health Centre. .

The lists will be generated by an independent statistician, and the code will be held by the DSMB.

Administration of second and third treatment doses will be placebo controlled.

### Pre-packaging of drugs

Drugs and their respective placebo will be packaged in the PNG IMR into Zip-loc bags. The bags will be marked with the treatment groups A to H.

If the drugs are vomited within 1 hour of administration, replacement drugs will be issued from another bag using the same code.

### Allocation Concealment and Blinding

The study will be conducted as single blind trial. Second and third treatment doses will be SP and AZ or matching placebo. The treatment allocation codes (i.e. groups A to H) will be held in individual, opaque paper envelopes. The treatment allocation will only be revealed after completion of enrolment by opening the envelope immediately prior to treatment. Only staff directly involved in treatment provision will be provided access the allocation code. Study staff working at other clinical positions, laboratory as well as staff of participating Hospitals and health centres will be kept blinded in regards to the treatment allocation.

The code will be held by the DSMB, principal investigators, study coordinator and staff directly involved in treatment. The code will only be revealed other staff once all women have completed the intervention and have been followed up to delivery, and data has been entered and checked, and data bases are locked. Treatment allocation will be revealed and the effect of SP/AZ on primary and secondary outcome measures will be evaluated. Participants, field and laboratory staff will remain blinded for the entire duration of the study.

In order to assure that treatment allocation is concealed from non-treatment staff as much as possible, staff with access to the treatment codes will sign a confidentiality agreement prohibiting the revelation of the codes to other staff and participants.

# Study Schedule

**Table 2: Summary of scheduled study activities**

|  |  |  |  |
| --- | --- | --- | --- |
|  |  | **Time** | **Activities** |
|  |  |  |  |
|  |  |  |  |
| **Enrolment Visit** |  | 1^st^ antenatal care visit at < 14 -20 weeks of gestation | - Identification of eligible mother - Enrolment - Baseline Data collection - Collection of venous blood sample - Collection of vaginal swab - Ultra Sounds examination - 1^st^ IPTp treatment |
|  |  |  |  |
| **2^nd^ Treatment Visit** |  | At 22-26 weeks of gestation | - 2^nd^ IPTp treatment - Finger prick blood sample - Collection of data on net use, health seeking, etc - Collection of vaginal swab - Treatment for STIs |
|  |  |  |  |
| **3^rd^ Treatment Visit** |  | At 28-32 weeks of gestation | - 3^rd^ IPTp treatment - Finger prick blood sample - Collection of data on net use, health seeking, etc |
|  |  |  |  |
| **Delivery** |  | At delivery | - Health assessment of mother & child - Measurement of child’s birth weight, & maternal Hb - Collection venous blood & placental blood sample and placental specimen from mother - Cord blood collection from child |
|  |  |  |  |
| **6 Weeks Follow-up** |  | 6 weeks after delivery | - Health assessment of mother & child - Collection of heel prick blood sample from child |
| **3 months follow up** |  | 3 months post delivery (250 consecutive women) | - Health assessment of mother & child - Collection of venous blood from mother |
|  |  |  |  |
| **1 year Follow-up** |  | 1 year after delivery | - Health assessment of child |
|  |  |  |  |

A graphic representation of study activities is given in Appendix I.

## Enrolment

Upon presentation at first ANC visit, the PNGIMR study team will determine eligibility of participants (see 5.1 above for criteria) and administer written informed consent (see 14.3 for details of consent procedures).

Upon consent the baseline health assessment will be conducted and women will be assessed for the following exclusion criteria:

- chronic illness,
- symptomatic severe anaemia (Hb < 6 g/dl with symptoms such as breathlessness, weakness, dizziness), or
- permanent disability, that prevents or impedes study participation

Any one or more of the above criteria is sufficient to exclude women from study participation:

Women with severe anaemia and no, or minimal, symptoms, can be enrolled but will be treated according to local guidelines with a full course of antimalarial (SP+CQ, or SP + AZ, depending on randomisation group) and iron replacement treatment.

In the event an eligible woman is excluded from the study, the PNGIMR field study team will record the reason for exclusion in the study log book. Women diagnosed with symptomatic severe anaemia will be referred to Modilon hospital for treatment.

Enrolment and 1^st^ treatment will be done at the same time, provided the woman is >13 weeks’ gestation.

Details of the procedures of Enrolment & 1st treatment Visit are given under 6.1 above.

Community controls will be enrolled in their villages by IMR staff, following village tok saves. Villages will be selected based on enrolment of pregnant women from these villages.

## Treatment & Follow-up Visits

**7.3.1 Treatment Visits**

Following enrolment and 1^st^ treatment women will receive further IPTp treatments at two intervals, at least 4 weeks after the previous administration. For details of procedure see 6.1.4 b & c.

At every ANC visit, only women who spontaneously report illness to study or ANC staff will be assessed on the spot for presence of malaria infections or anaemia.

Women found to have symptomatic severe anaemia (Hb < 6 g/dl) or other severe illness will be referred to the health centre for appropriate treatment. They will not be suspended from participation in the study but will be monitored again for anaemia and clinical status at their next ANC visit. Should their condition not have resolved by that time, they will be considered chronically ill and excluded from further participation in the study.

**7. 2. 2 Non-treatment ANC visits**

At ANC visits when treatment is not administered, women will be seen by Health Centre/Hospital staff only. Blood pressure, weight, fundal height and other indicators of maternal health will be recorded. Should women have symptoms or signs warranting further investigation, they will be referred to study staff, and an Unscheduled Visit form will be completed. Women with hypertension will have physical examination and urinalysis to seek other evidence of pre-eclampsia. Study or Hospital physicians will assess women as requested by study and health centre nursing staff.

**7.2.3 Delivery**

At delivery, women will receive standard care from Health Centre or Hospital staff, assisted as necessary by study staff. Maternal venous blood (10 ml) will be collected either before or within 24 hours of delivery. Maternal haemoglobin concentration will be measured, and blood smears prepared. Plasma, PBMCs and red cells will be separated and stored, as detailed in the separate immunology protocol.

Samples will be collected from the placenta, for blood smears, histology and immunological studies. Cord blood samples will also be collected. The baby will be weighed, and gestation assessed using a modified Ballard’s assessment.

**7.2.4 Post delivery infant and maternal follow up**

Women and their babies will be seen at 6 weeks post partum and babies only at 12 months of age. Heel prick blood will be collected at 6 weeks (see also 6.1.5 b). A subset of 250 women will be followed on one further occasion, at 3 months post partum, to provide a further venous blood sample.

## Early Termination Visit

A woman might be terminated early from the study by either withdrawing from the study or by permanently moving outside the study areas. In addition the woman may be terminated from the study following the occurrence of a serious adverse event (SAE see below), that prevents the continued presence of the participant in the study.

In case of an early termination, the date and reason for termination will be noted in the participants’ database. No specific early termination visit will be done. If the woman’s participation is terminated due to a serious adverse event, the termination will also be noted on the SAE forms.

## Criteria for Discontinuation or Withdrawal of a Subject

**Participants may withdraw at any time from participation in this study.**

A participating woman will be discontinued from the study if one of the following criteria are fulfilled:

- the participant moves away from the selected study area for a period longer than 2 months
- the participant develops a chronic illness that impedes continuation in study.
- the participant displays or develops severe side effects to study drugs
- the participant dies.

# Assessment of Outcome MeasureS

## Specification of the Appropriate Outcome Measures

### Primary Outcome Measures

Low birth weight is defined as weight within 7 days birth of <2500 g. For Health Centre or Hospital deliveries, it will be recorded within the first hour of life. For home deliveries, it will be recorded by study staff within 7 days of delivery. Babies will be weighed at study health centres where possible. Otherwise a mobile team will visit them in the village.

### Secondary Outcome Measures

1. ***Prevalence of P. falciparum at 2^nd^ treatment time point and delivery***

The prevalence of *P. falciparum* in peripheral, placental and cord blood films will be determined by light microscopy and PCR-LDR. Prevalence of malaria parasites in placental histology will be determined by light microscopy and (where applicable) immuno-histochemistry. Imprint smears from placenta will be used to assess parasite morphology and assist in speciation.

1. ***Mean maternal hemoglobin concentration at delivery, and proportion of women anaemic (Hb < 11 g/dl).***

Hemoglobin concentration will be determined the Hemacue system. Where applicable confirmation may be done using a Coulter ActDiff system.

1. ***Prevalence of P. vivax at enrolment, second treatment, and delivery***

The prevalence of *P. vivax* in peripheral, placental and cord blood films will be determined by light microscopy and PCR-LDR. Prevalence of malaria parasites in placental histology will be determined by light microscopy and (where applicable) immuno-histochemistry. Imprint smears from placenta will be used to assess parasite morphology and assist in speciation.

1. ***Incidence of symptomatic malaria during pregnancy***

Symptomatic malaria will be monitored through passive case detection at participating health facilities. Symptomatic malaria will be defined as an axillary temperature >37.5°C or a history of febrile illness and (microscopically) confirmed malarial infection of any density.

1. **Proportion of women carrying azithromycin-sensitive sexually transmitted infections at second treatment visit (28-34 weeks).**

The prevalence of *N gonorrhoeae*, *C trachomatis* and *T vaginalis* will be determined by multiplex PCR from self-collected high vaginal swabs.

1. ***Incidence of Adverse Events, including severe adverse events (SAEs), and AEs possibly or probably associated with study medications***

For definitions and procedures of AEs see Section 9 below

1. **Prevalence of drug resistance markers in parasites infecting women in late pregnancy**

The prevalence of molecular markers of SP resistance will be determined in randomly selected, PCR-positive samples using an established PCR-LDR assay.

1. **Prevalence and antibiotic sensitivity patterns of *S. pneumoniae* in nasopharyngeal swabs collected at delivery**

The prevalence of carriage of S pneumoniae and of antibiotic resistance will be determined in nasopharyngeal swabs by routine culture and disc diffusion, respectively. Isolates will be stored for further characterization.

1. ***Maternal, perinatal and infant mortality rates.***

The following mortality rates will be determined:

1. Maternal mortality rate (enrolment to 6 weeks post partum). Determined by home visits to communities where mothers lived, and interview of family members and other relevant individuals.
2. Abortion/stillbirth rates. These are defined as death of a fetus before delivery. Abortion is a death before 22 weeks estimated gestation and stillbirth is a death at or after 22 weeks gestation
3. Perinatal mortality rates. These are defined as death (due to any cause) of a child from 22 weeks to 7 days post partum.
4. Infant mortality rate (0-12 months). All cause infant death will be recorded.
5. **Impact of IPTp on development of immunity to malaria in pregnancy**

A detailed description of immunological outcome measures will be given in a separate immunology sub-protocol.

1. **Characteristics of parasites infecting pregnant women.**

A detailed description of parasitological outcome measures will be given in a separate immunology sub-protocol.

# Safety assessment and reporting

Safety of our intervention is paramount. Although all drugs to be used have good safety records, adverse events will be diligently sought. Before each dose is administered, participants will be asked for new symptoms or signs, with specific questions regarding skin rash, or easy bruising or bleeding.

Although remote, there is a small, finite risk of infection associated with finger prick and venous blood draws; we do not anticipate that there will be any study-related AEs. Sterile procedures will be used during blood draws and all sharps and contaminated wipes or other objects will be disposed of properly as described in the Field and Laboratory Procedures Manual.

## Definition of Adverse Event (AE)

Possible adverse events will be assessed by the study clinician and graded in severity according to tables modified from those of the NIH Division of Microbiology and Infectious Diseases. Briefly, events will be graded in severity as:

Grade 1 (mild, self limited (<48 hours), no intervention required).

Grade 2 (moderate: some limitation in activity, some assistance possibly required; no or minimal medical intervention required).

Grade 3 (severe: marked limitation in activity; some assistance usually required; medical intervention required; hospitalization possible).

Grade 4 (life-threatening: extreme limitation in activity; significant assistance required, significant medical intervention required; hospitalization probable).

Grade 5 adverse event results in death

Any clinical event determined by the clinician to be serious or life threatening will be classed as grade 4. Such events include (but are not restricted to): seizures, coma, disseminated intravascular coagulation, desquamating skin rash suggestive of Stevens-Johnson syndrome, diffuse petechiae, paralysis, and altered behaviour suggestive of neurological disturbance.

## Definition of Serious Adverse Event (SAE)

Any untoward medical occurrence that:

- Results in death.
- Is life-threatening.
- Requires in-patient hospitalization or prolongation of existing hospitalization.
- Results in persistent or significant disability or incapacity or required intervention to prevent permanent impairment or damage.
- Is a congenital anomaly or birth defect
- Medical judgement is required in certain other circumstances such as cases requiring intensive outpatient management, blood dyscrasias or convulsions that do not require hospitalisation, and similar events.

Is to be considered a serious adverse event (SAE) and will be assessed following the PNGIMR SOPs for SAEs.

## Reporting Procedures

The DSMB and the Malaria in Pregnancy Safety Working Group will be notified by the principal investigators or their designate within 48 hours about SAE / Grade 4 AEs, while Grade 1-3 AEs will be reported 3 monthly in summary fashion.

### Serious Adverse Event Detection and Reporting

For all events meeting the above described definition of Serious Adverse Events and/or Grade 4 AE, the completion of the Serious Adverse Event report form is required.

The study physicians will inform the PIs immediately of any SAE or Grade 4 AE and complete a Serious Adverse Event Form within the following timelines:

- All deaths, whether associated or not associated, will be recorded on the Serious Adverse Event Form. The Form will be sent by fax to PIs within 24 hours of site awareness of the death.
- Serious adverse events other than death, regardless of relationship, will be reported via fax by the site within 72 hours of becoming aware of the event.

Other supporting documentation of the event will supplied as requested.

The SAE Form will be reviewed by the PIs or their designate, who may request additional information or carry out further investigations. Additionally, an independent local clinician will be appointed to review all SAEs and prepare separate reports for the PIs,DSMB and Malaria in Pregnancy Consortium Safety Working Group

The Principal Investigators will notify the DSMB and Malaria in Pregnancy Consortium Safety Working Group immediately of any serious adverse event, deaths or life-threatening problems that occur in the study within 48 hours and supply all subsequent information at the earliest.

If a grade 4 event is unexplained, or is attributed to study medication, the DSMB chair will instruct the investigators to report the event to the IMR Institutional Review Board, the PNG Medical Research Advisory Committee (MRAC) and the Royal Melbourne Hospital Human Research Ethics Committee (RMH HREC). Reports to the DSMB of severe adverse events (not believed to be due to the study drug) will be submitted to the IRBs electronically on a two-monthly basis.

Reporting of data to the MiP Consortium safety working group will occur in parallel to reporting to the DSMB, e.g. by copy of email report submissions.

Subjects will be withdrawn from the study if they experience a drug related SAE / grade 4 AE.

### Procedures to be followed in the Event of Abnormal Laboratory Test Values or Abnormal Clinical Findings

If a participant is found to have symptomatic severe anaemia (Hb < 6 g/dl with anaemia symptomatology) she will be referred to Modilon Hospital for treatment.

The study coordinator will report such cases as SAE to the PI who will inform the DSMB and MiP SWG.

### Type and Duration of the Follow-up of Subjects after Adverse Events

Management of adverse events will be according to best available practice. The study team will provide referral and transport to Madang (Modilon) Hospital where investigation and treatment will be provided through Government facilities.

All SAEs will be followed until satisfactory resolution or until the Principal Investigator or Study Clinician deems the event to be chronic or the patient to be stable.

## Halting Rules

There are no predefined halting rules, but the DSMB, IMR IRB, PNG MRAC and RMH HREC will each have the power to suspend the study, pending full review of toxicities encountered. The MiP Safety Working Group can make recommendations to the study investigators, DSMB and the IRBs but cannot order stopping or alteration of the study.

# Clinical Monitoring Structure

## Site Monitoring Plan

This study will be monitored both by a specifically constituted Data & Safety Monitoring Board (DSMB) and by an external clinical monitor.

The DSMB will have 3 members a local PNG physician, an Australian physician and a data statistician. The DSMB will generate and hold the allocation code, and will review documentation of all SAE / Grade 4 AEs. Before referral to the DSMB, all SAEs will be reviewed by a locally appointed clinician.

An independent clinician based in Madang will review the CRFs for all SAEs and Grade 4 AEs to ensure their completeness and accuracy, and to make an independent judgement regarding causality. Dr Paul Harianto has been appointed to this role

The study will be monitored externally by an external monitor who has experience with monitoring similar studies. S/he will be appointed centrally by the Malaria in Pregnancy Consortium, and s/he will visit the PNG field and laboratory sites every 12 months for the entire duration of the study (anticipated three visits total). S/he will assist the study team with implementation of the study according to ICH GCP standards and will report her/his findings to the principal investigators, the PNGIMR, the Malaria In Pregnancy Consortium Executive Committee, and the DSMB.

# Statistical Considerations

## Study Outcome Measures

The goal of this Protocol is to study assess the efficacy of intermittent preventive treatment with SP&AZ in improving birth outcomes in PNG women. In particular, will determine:

Primary Outcome Measures

- 1. Prevalence of low birth weight (<2500 g) in women receiving SP/AZ and controls

Secondary Outcome Measures

- 1. Prevalence of *P falciparum* at delivery in peripheral, placental and cord blood films and on placental histology, and at second visit by PCR only in peripheral blood films.
  2. Mean maternal hemoglobin concentration at delivery, and proportion of women anaemic (Hb < 11 g/dl).
  3. Prevalence (at enrolment, second treatment, and delivery) and consequences (maternal haemoglobin, birth weight and placental pathology) of *P. vivax* infection in pregnancy
  4. Immune responses to P vivax in pregnant and non-pregnant women
  5. Incidence of symptomatic malaria during pregnancy
  6. Proportion of women carrying azithromycin-sensitive sexually transmitted infections at second treatment visit (28-34 weeks).
  7. Incidence of Adverse Events, including severe adverse events (SAEs), and AEs possibly or probably associated with study medications
  8. Prevalence of drug resistance markers in parasites infecting women in late pregnancy
  9. Prevalence and antibiotic sensitivity patterns of *S. pneumoniae* in nasopharyngeal swabs collected at delivery
  10. Maternal, perinatal and infant mortality rates.
  11. Impact of IPTp on development of immunity to malaria in pregnancy

### Definitions

**Malaria infections: P falciparum and P vivax (Secondary objectives 2 and 4)**

**Microscopy**

Blood smear microscopy is the cornerstone for determining the presence of Plasmodium infections. Blood smears will be evaluated for each type of Plasmodium species and separate counts will be recorded for blood-stage parasitemia of each species and *P. falciparum* gametocytemia. A blood smear will be read as the number of a given Plasmodium spp. parasitized erythrocytes per parallel count of 200 leukocytes. The average leukocyte count for individuals in coastal PNG is 8,000 per µl. Therefore, the parasite count (Plasmodium spp. parasitized RBCs/µl) will be determined by multiplying the number of parasitized RBCs by 40. All blood slides will be read independently by two expert microscopists.

**PCR**

In addition, we will also determine the presence of malaria parasites using PCR-LDR typing (see 6.2.1.2 a). This method has a higher sensitivity than light microscopy and it is therefore common to find infections that are detectable by PCR-LDR, but not by microscopy. Samples will be considered positive for any infections if the mean fluorescent intensity exceed a species specific threshold defined by the mean + 2 SD of negative controls.

Histology

Placental biopsies will be collected at delivery, fixed in 10% neutral buffered formalin,and paraffin embedded. Sections cut onto glass slides will be examined following staining with Giemsa and haematoxylin and eosin. Placentas will be classified as (a) active infection (presence of any parasitised erythrocytes) (b) past infection (presence of malaria pigment haemozoin, in fibrin deposits or in mononuclear leukocytes) and (c) no infection. Active infection will be further divided into active acute (presence of parasites, but not of haemozoin deposits) and active chronic (parasites, and malaria pigment in leukocytes or in fibrin deposits).

Maternal haemoglobin concentration

Measurement of haemoglobin by Hemocue cuvette will be used. Anaemia will be defined as Hb <11.0 g/dl, moderate anaemia as Hb 6.0-8.9 g/dl, and severe anaemia as Hb <6.0 g/dl.

Symptomatic Plasmodium Infection

For diagnostic purposes, a symptomatic Plasmodium infection is defined as any type of Plasmodium parasitemia observed by blood smear and/or a positive rapid diagnostic test, plus an axillary temperature ≥ 37.5° C or a history of febrile illness in last 48 hours.

This definition will be used for analysis of the secondary outcome of symptomatic malaria in pregnancy.

However, for analytical purposes we will use two additional definitions for symptomatic Plasmodium infections: a) any type of Plasmodium parasitemia observed by blood smear and/or PCR/LDR diagnostic assay, plus an axillary temperature ≥ 37.5° C or a history of febrile illness in last 48 hours b) a parasite density of > 1000/μl for *P. falciparum* and/or > 250/μl for non-falciparum, plus an axillary temperature ≥ 37.5° C or a history of febrile illness in last 48 hours.

Asymptomatic Blood-stage Plasmodium Infection

An asymptomatic blood-stage Plasmodium infection is defined as any type of Plasmodium parasitemia observed by blood smear or PCR-LDR confirmation with an axillary temperature less than 37.5° C and no history of febrile illness in last 48 hours.

**Carriage of sexually transmitted infections**

Seropositivity for syphilis will be defined as presence of positive RPR, confirmed by a positive TPHA. Chlamydia trachomatis, Trichomonas vaginalis and N. gonorrhoeae infections will be defined as positive PCR on swabs collected at enrolment and at 28-34 weeks.

### Primary Outcome Measures

1. **Percentage of babies born with low birth weight**

This is the primary objective of the study and will be evaluated by assessing difference in proportion of LBW deliveries between the 2 treatment groups.

When women give birth outside of health facilities, they will be instructed to present with their newborns for checkups as soon as possible, and in any case within 7 days of delivery. Childhood immunisation will be commenced, maternal health assessed, and infant weight measured. Weight within 7 days of birth can be approximated to birth weight.

Only women who receive at least 1 IPTp treatment and have a recorded pregnancy outcome (abortion, still birth, or live birth) will be included into this analysis. This restriction applies to all analyses except for analyses of reported drug side effects (see 9. below).

### Secondary Outcome Measures

1. **Prevalence of *P. falciparum* malaria at delivery by microscopy**

This outcome measure will be analysed in women in whom blood films (peripheral and/or placental) are collected at delivery.

1. **Prevalence of *P. falciparum* malaria at delivery by histology**

This outcome measure will be analysed in all women from whom an assessable placental biopsy is collected at delivery.

1. **Maternal Hb at delivery by histology**

This outcome measure will be analysed in all women from whom an Hb measurement at delivery is available.

1. **Incidence of symptomatic malaria**This outcome will be analysed in all women that have had 1 IPTp treatment
2. **Prevalence of STIs on Vaginal swab at 28-34 weeks gestation**

This outcome measure will be analysed in all women in whom an assessable swab is collected at 28-34 weeks. Rates of carriage of N gonorrhoeae, C trachomatis and T vaginalis (assessed by PCR) will be compared between groups.

1. **Maternal, perinatal and infant mortality rates**

All deaths, irrespective of cause of death, occurring in the cohort of pregnant women and their fetuses or children between Enrolment and Final Evaluation Visit (6 weeks post partum for pregnant women, deaths from 22 weeks gestation to 28 days post partum for perinatal mortality, deaths from day 28 of life to 12 months of age for infants) will be considered for this analysis

1. **Reported side effects / Adverse events**

All reported side effects following IPTp treatment, irrespective of total number of treatments a woman received, will be considered for these analyses.

1. **Molecular markers of SP resistance**

This endpoint will be assessed in a random sample of PCR-positive samples collected at 2^nd^ treatment and at delivery.

## Sample Size Considerations

The primary objective of the study is to determine the effect of IPTp with SP/AZ compared to SP/CQ clearance treatment on prevalence of low birth weight (LBW) in women using ITNs. The overall power and sample size calculations are thus based on this outcome.

The analyses for this primary outcome measure will use Poisson regression (with or without random effects). Sample size calculations for these types of analyses are non-trivial and often require extensive simulations. For that reason we are using an approximate sample size calculation based on comparison of two Poisson rates.

**Table 3**: Sample size calculations for principal outcomes using appropriate two sided tests, a significance level of 5% and a power of 80%.

|  |  |  |  |  |
| --- | --- | --- | --- | --- |
| **End points** | **Expected Outcomes** | |  | **Sample Size** |
|  | Control | AZ-SP |  |  |
|  |  |  |  |  |
|  |  |  |  |  |
| **Primary** |  |  |  |  |
| Prevalence of LBW | 12% | 8.4% |  | 1163 |
| Mean birth weight | 2.94kg | 3.01kg |  | 970 |
|  |  |  |  |  |
| **Secondary** |  |  |  |  |
| Prevalence of *P. falciparum* at delivery (peripheral & placental) by PCR | 30% | 20% |  | 389 |
| Prevalence of malaria in placental histology | 30% | 20% |  | 389 |
| Mean Hemoglobin at delivery | 10.0 | 10.3 |  | 845 |
| Prevalence of bacterial STI at 2^nd^/final treatment time point | 30% | 10% |  | 72 |
|  |  |  |  |  |

Previous studies suggest that IPTp with SP can increase birth weight by approximately 130 g. ITNs increase birth weight by around 60 g. The effect of adding SP to ITNs is not clear, but we assume that it increases mean birth weight by approximately 70 g. LBW is seen in 16% of babies born to women not using ITNs in PNG. If ITNs decrease LBW by 25% then we will expect 12% of babies to be LBW. SP+AZ may have additional benefits (through control of STIs which impair fetal growth) over SP alone. Our conservative estimate is that SP + AZ will decrease LBW prevalence by 30%. Based on these assumptions, a sample size of 1,163 and 970 women per arm, respectively, are required to find a 30% reduction in LBW and a 70g increase in birth weight with a power of 80% (Table 3).

Assuming, a loss of 20% during follow-up, we will thus enroll 1396 women per arm.

Assuming that 40% of women have malaria on histology and that the introduction of ITN will decrease this by 25%, we expect a prevalence of malaria on histology of 30%. Based on this assumption and an expectation to collect a good quality placental specimen from 60% of all enrolled women, the chosen sample size is sufficient to detect a 29% reduction in prevalence of malaria histology in women receiving IPTp AZ-SP with a power of 80%(two sided test). This sample size will also be sufficient to detect a 22% reduction in *P. falciparum* prevalence at delivery, a reduction in prevalence of STIs from 25% to 20% and/or a 0.26g/dl increase in maternal Hb with a power of 80%.

An interim analysis will be performed when 1400 women enrolled in the study have delivered and had birth weights recorded. Analysis will compare percentage LBW between control and intervention groups.

Sample size calculations were done [STATA](../STATA) 8 software (College Station, TX)

## Participant Enrolment and Follow-Up

This study will enrol 2792 pregnant women over a period of 18-24 months. Enrolment and follow up will occur through Hospital and Health Centre Antenatal Clinics. Apart from some ultrasound visits (particularly at enrolment), no additional study visits will be required, beyond visits for standard antenatal care.

Currently there are high levels of ANC attendance in Madang. If women miss a treatment or follow-up visit they will be visited on the next day by a village reporter and encouraged to visit the health centre or the hospital ANC. We therefore expect >90% attendance at any follow-up time point.

However, people in the study areas do occasionally move the village of residence. In addition some people do grow tired of participating in intensive longitudinal follow up studies and withdraw consent for continued participation.

Women that missed more than 2 treatments will be excluded from all analyses except those on drug side effects. Consequently, we expect about 20% loss of follow-up.

## Analysis Plan

### Primary Outcome Measures

The analyses for the primary outcome measures will use logistic regression. The primary outcome will be defined as number women giving birth to a child with a birth weight of <2500g. These analyses will adjusted for different covariates such as sex, gestation, genotype, bed net use, number of IPTp treatments received, season of delivery (wet vs dry) and site.

Differences in birth weight will be assessed using analyses of variance (ANOVA) and adjusted for the same covariates.

### Secondary Outcome Measures

Logistic regression will also be used to analyses parasite prevalence at delivery (for all 4 Plasmodium species individually as well as combined) and prevalence of STIs at 2^nd^/final treatment time point. Mean haemoglobin levels will be compared using ANOVA.

Statistical analyses will be done using STATA, SPLUS and SAS statistical software.

# Access to Source Data/Documents

Each participating site will maintain appropriate medical and research records for this study, in compliance with Section 4.9 of ICH E6 GCP and institutional requirements for the protection of confidentiality of subjects. Each site will permit the clinical monitor and authorized representatives of DSMB or IRBs to examine (and when required by applicable law, to copy) clinical records for the purposes of quality assurance reviews, audits and evaluation of the study safety and progress.

Source data are all information, original records of clinical findings, observations, or other activities in a study necessary for the reconstruction and evaluation of the trial.

# Quality Control and Quality Assurance

This study will be conducted according with the procedures outlined in this protocol.

In order to assure both compliance with the protocol and assure the quality of the data collected local quality control procedures will be put in place. The different tasks to be performed in field and lab will be defined in specific standard operating procedures (SOPs). Adherence to both protocol and SOPs will monitored via regular spot checks on collection of primary field data, checks of all forms by study coordinator or designee upon receipt from field teams, double reading of all blood slides collected and regular data audits following 2^nd^ entry of all databases. In addition, the senior investigators will regularly perform internal audits that assess completeness and accuracy of study files, CRFs and source documents and ethical standards of study operations.

All specimens collected as well as their transfer between different sites will be recorded in specimen log book. The site laboratory investigators will regularly check lab books and documents to assure compliance with laboratory SOPs.

The detailed quality control and quality assurance procedures will be set out in specific standard operating procedures (SOPs) for quality management.

# Ethics/Protection of Human Subjects

## Declaration of Helsinki

The investigators will ensure that this study is conducted in full conformity with the current revision of the Declaration of Helsinki, or with the International Conference for Harmonisation Good Clinical Practice (ICH-GCP) regulations and guidelines, whichever affords the greater protection to the subject.

## Institutional Review Board

The participating institutions are reviewed by the following Institutional Review Boards:

PNGIMR: PNG IMR Institutional Review Board and PNG Medical Research Advisory Committee (MRAC).

WEHI: Institute Ethics Committee

University of Barcelona Ethics Committee

CGHD: Cleveland Hospital IRB (DHHS IRB00001691 & IRB00000684)

Univ. of Melbourne: Royal Melbourne Hospital Human Research Ethics Committee

Any amendments to the protocol or consent materials will be submitted for approval before they are placed into use.

## Informed Consent Process

Following standard practice for PNGIMR field studies, the informed consent process starts well prior to enrolment of participants and involves both community and individual consent.

Upon funding approval, the study was discussed with representative of provincial and mission health services as well as senior community members including community women’s groups to assess both feasibility and community acceptability of different study design features and field procedures. Comments and suggestions by community and health services were integrated into the final study protocol.

As a first step in the informed consent process, the PIs and community liaison staff will hold meetings with community leaders / elders in all potential study villages. At these meetings study objectives and procedures as well as risks and possible benefits for the individual participants and the community will be discussed in detail. The study brochure (see below) will be presented to the community leaders, but no written consent sought.

Following consent by community leaders to include their village into the study, the study team will invite all interested community members in particular young women and their partners to study information meetings (tok save in Melanesian Pidgin) held in several central locations throughout the study areas. At these meetings, the study team will describe the purpose and significance of the study, the procedures to be followed, the risks and benefits of participation, and state that participation in the study is voluntary and that declining to participate will not reduce the level of, or access to, health care for the eligible pregnant women. The study brochure will be made available (upon request) to interested community members.

Eligible women will be identified at first antenatal clinic attendance (see 6.1.3). After identification of eligible women the study team will advise prospective study participants about the study, its purpose, procedures as well as possible risks and benefits. The study team will stress that participation in the study is completely voluntary and that refusal to participate will not lead to reduce the level of, or access to, health care. At the end of the tok save the woman will be given a copy of the informed consent document and asked to consent to participation. The Enrolment and 1^st^ Treatment Visits are usually contemporaneous the exception is when a woman is <13 weeks gestation, when she will be asked to re-attend early in the second trimester.

Many people in Papua New Guinea, especially those with limited literacy skills are reluctant to sign lengthy documents. In order to accommodate this, the study will use two distinct informed consent documents: a detailed study brochure and a shorter informed consent signature form. The study brochure contains all the necessary information for informed consent in easily understandable language, supplemented by illustrations of study procedures. The informed consent signature form contains shortened summary of the study information and a signature page. Both forms are included in Appendix III & IV. All informed consent documents will be approved by the PNG IMR IRB and MRAC and overseas IRBs.

The tok saves will be held in Melanesian Pidgin and parents of the prospective participant will receives consent documents in the language in which the participants are most conversant (Melanesian pidgin or English). The study team will answer all questions the women may have prior signing of the informed consent signature form.

**Only women who have signed the informed consent signature form will be enrolled in the study.**

In the event the participants decline to participate, the PNGIMR field study team will record the reason in the study log book.

**The participant may withdraw consent for their participation at any time throughout the course of the study.**

At any time of the study, the study team will be available to answer questions of participants regarding any aspect of the present study.

Participants in this protocol will take part in in-depth immunology studies and drug resistance monitoring, provided they meet study eligibility criteria. The study brochure will contain all necessary information for these studies and no separate written informed consent will thus be administered.

Sample from this study may also be used for further laboratory studies on malaria and anaemia related research questions and the informed consent signature form contains a question for participants to authorise further use of sample for such studies.

DNA from mothers and babies may be used for host genetics studies, which will be subject of a separate application. However, consent for use of samples for such studies will be included

## Exclusion of Women, Minorities, and Children (Special Populations)

The study participants are all pregnant women at enrolment. The study expects these women to give birth to an approximately equal number of male and female children.

## Subject Confidentiality

The confidentiality of participant information will be maintained at all times. Participant information will be identified by the study identification number and/or bleed code and serial number as appropriate. Blood samples will be tagged using the appropriate unique bleed codes and serial numbers.

The study protocol, documentation, data and all other information generated will be held in strict confidence. No information concerning the study or the data will be released to any unauthorized third party without prior written approval of the sponsor.

The study monitor, the DSMB or other authorized representatives of the sponsor may inspect all documents and records required to be maintained by the Investigator, including but not limited to, medical records (office, clinic or hospital) and pharmacy records for the subjects in this study. The clinical study site will permit access to such records.

The investigators will generate summary reports on the epidemiologic features of malaria for children by village. An annual report will be made to the Malaria in Pregnancy Consortium, and supervising IRBs in PNG and Melbourne as required.

## Future Use of Stored Specimens

Specimens collected in this study will be used for a series of collaborating studies. Theses studies will be detailed in the immunology sub-protocol

# Data Handling and Record Keeping

## Data Management Responsibilities

The primary data will be collected by field and laboratory staff at the different field and laboratory sites.

Upon receipt of CRF and source documents from the field team, the study coordinator (or his/her designee) will cross-check all CRF’s for completeness, validity and legibility.

All data from CRFs and laboratory worksheets in PNG will be entered into a custom made study database set up using the DMSys Clinical Trial Data Management software. The database will confirm with all obligations under ICH GCP rules.

The database will be programmed and maintained by the PNGIMR database manager in Madang. This database uses double entry of batched data and allows thorough data consistency checks, queries and data cleaning on individual data batches as well as the complete data set. In addition it allows full audit of all inconsistencies, queries and changes made to the databases

Data entry will be done in batches. First entry will be done by trained data entry staff at the designated data entry unit directly after receipt of CRF. Second entry will be once a batch of first entry is completed. Discrepancies, missing values, and out-of-range values will be detected in queries run on a batch-by-batch basis by the data base manager. If necessary queries will be send back to the field site for resolution. All corrections made to the database by the data managers (or designees) will be recorded. The forms will be kept in separate files denoting the stage of data entry (e.g., to be entered, to be queried, completed). Upon resolution of all queries the batch will be merged into a locked master file.

Lab workbooks of lab work conducted in Goroka, Melbourne or Barcelona will be maintained and entered on site. In Goroka data will be entered into independent DMSys database using the same procedures outlined above. Entry and cleaning of laboratory data collected in Melbourne will be conducted on site under the supervision of the site investigators. Electronic copies of the final, cleaned data will be sent to the data manager in Madang.

Electronic versions of the locked datasets relating to this study will be maintained as a part of the PNGIMR’s research studies database. Access to the study database will be limited to the data entry personnel, data managers for the PNGIMR, the Study Physicians, Protocol Epidemiologist / Statistician, and Senior Investigators. The database for the study will be backed up on regular intervals. Copies of the databases generated in Goroka and outside PNG and interim data sets will be sent to the data manager in Madang at regular intervals. At the end of the study, the final database and analytical datasets for the study will be maintained on high-end computers located at PNGIMR, WEHI, UoM, U Barcelona and CGHD.

## Data Capture Methods

Source documents in this study will consist of the following:

- The Field study logbook relating to study enrolment and continued participation in cohort;
- Case Report Forms (CRFs) completed by the field study team during study visits (whether scheduled or non-scheduled) with each participant;
- Health Centre logbooks;
- Health Centre Surveillance form abstracts documenting illness and treatment histories for participants who seek care from participating health Centres;
- Children’s personal health books
- Microscopy Laboratory worksheets documenting the evaluation of blood smears;
- Laboratory notebooks

All data recorded in the study logbook and laboratory notebooks and on CRFs and laboratory worksheets will be legibly recorded in black or blue ink. The study coordinator and site investigator (for laboratory data) will check all CRFs prior to passing the forms on to data entry. If corrections need to be made, the incorrect entry will be crossed out with a single line and the correct information will be printed adjacent to it. The correction must be initialled and dated by designated, qualified study staff. Any requested information that is not obtained as specified in the protocol should have an explanation noted on the CRF as to why the required information was not obtained.

## Types of Data

The study will collect the following types of data:

- Demographic data (at enrolment only)
- Treatment data (IPTp and curative treatments)
- Behavioural data (e.g. bed net use, betel nut consumption)
- Delivery data
- Haemoglobin measured at delivery
- Prevalence and intensity of malaria infections by LM and PCR-LDR
- Anthropometric data for baby (birth weight, length, etc)
- Morbidity data (at times of symptomatic episodes)
- In a subset of women, analysis of immunity to *P. vivax*
- Measures of antibody immunity to *P. falciparum*
- *P falciparum* gene expression data using peripheral and placental blood samples from participants
- In future, data on gene polymorphisms in mother and baby that may predict pregnancy outcome.
- Genetic data on major red blood cell polymorphisms (i.e. α-thalassemia, Gerbich, Duffy, SAO, G6PD)

## Timing/Reports

Data entry and review will be ongoing activities during the entire field study period. Data will be frozen upon completion of data entry and cleaned prior to any data analyses.

## Study Records Retention

Hard Copies of study records will be maintained for at least 5 years following completion of the study.

Electronic copies of all frozen databases will be kept indefinitely at the PNGIMR in Goroka.

## Protocol Deviations

The protocol will be adhered to for the entire duration of the study. In particular, no deviations from enrolment and exclusion criteria are permitted.

Adherence to protocol will be monitored by the study coordinator and site PIs as well as by the external monitor. In addition, protocol violations (e.g. exclusion criteria) will be assessed regular as part of the QC/QA procedures.

Should protocol deviations occur, the study supervisor and external monitor will inform the principal investigator and sponsor of the nature of these deviations. The PI will then inform the IRBs and if indicated seek amendments to the protocol.

# Publication Policy

All results obtained from this study will be submitted to peer-reviewed biomedical journals for publication. Publication will be preceded by internal review by the Malaria in Pregnancy Consortium Executive Committee, as per the Consortium’s operating rules.

Summary results will be given back to participants and community on a yearly basis.

# Preparatory study

The proposed study is very large and complex. Setting up such a study poses significant logistical challenges and required careful planning, in particular as it involves working at several health facilities and requires testing for both malaria and STI. In order to facilitate the setting up the study and to test study procedures we are thus planning to conduct a limited preparatory study at Modilon Hospital. This study will follow exactly the sample protocol as the main trial, except that women will receive the control treatment of 1 dose of SP and CQ plus bednet. In addition, a number of women will be asked to provide a stool sample to determine the prevalence of intestinal helminths in pregnant women in PNG, Should the prevalence be high, we will consider providing anithelminthic treatment to all women in the main trial

This preparatory study will enrol between 200 and 300 women over a period of 3-6 months and follow them up to delivery, depending on how quickly the processes for extending the study to the other sites can be established and additional staff can be trained.

Besides serving as a preparation for the main trial, the study will contribute important data to a number of secondary objectives of the study, in particular to determination of

1. Prevalence (at enrolment, second treatment, and delivery) and consequences (maternal haemoglobin, birth weight and placental pathology) of *P. vivax* infection in pregnancy
2. Immune responses to P vivax in pregnant and non-pregnant women
3. Incidence of symptomatic malaria during pregnancy

As the non experimental treatment will be given to women in the preparatory study, a separate, simplified informed consent for will be used for women participating in this study (see Appendix V & VI).

# Data and Sample Sharing with P. vivax in Pregnacy (Pregvax) study

The current study will participate in an international multi-centre study that aims to quantify the burden and examine the pathology caused to mother and child by P. vivax infections in during pregnancy. World wide approximately 25 million pregnant women exposed yearly to malaria live in areas where *Plasmodium vivax* is endemic. While the effects of *falciparum* malaria in pregnancy have been well characterised and are responsible for considerable maternal and infant morbidity and mortality, surprisingly little is known about the impact of *P. vivax* infection during gestation. This PREGVAX multi-centre study aims to describe the epidemiological and clinical features of *vivax* malaria in pregnancy in Papua New Guinea, India, Brazil and Guatemala.

At all sites pregnant women will be enrolled at the time of routine antenatal care visits and followed-up at the health facility until delivery or end of pregnancy. *P. vivax* malaria parasitemia will be assessed at enrolment, at every contact with the health facility and at delivery. In a sub-sample of women, peripheral blood will be taken for immunological/molecular studies and placental samples will be collected. In addition to the clinical-epidemiological studies, immunological analysis will be performed to unveil whether there are pregnancy-specific immune responses. Lastly, phenotypic and genotypic analyses of parasites from the placenta should reveal, respectively, their adhesive properties and whether the accumulation of *P. vivax*-infected erythrocytes in the placenta selects unique parasite populations.

In PNG, this study will be conducted as an integral part of the current protocol.

Clinical and epidemiological observations for multicentre analyses will be obtained from women in the control group. Similarly, blood samples and placental pathology specimens collected as part of this protocol will be used to perform *P. vivax* specific laboratory analyses. The laboratory studies will be outlined in detailed a separate laboratory sub-protocol. Prior to sharing any data or samples for comparative multi-centre analyses, data and samples will be fully anonymised.

No supplementary data will be collected and no additional blood samples taken. The participation in this study does thus not add any additional risk to the women participating in the IPTp trial.

The informed consent documents in this protocol cover the collection of all data and samples required for the P. vivax objectives and include explicit permission by the participating women for samples from the study to be used for future malaria research studies. No separate consent will thus be performed for the studies.

As per requirements of the funding institution requires a separate ethics clearance, the PREGVAX proposal will be submitted to the IMR IRB in addition to the current protocol.

# Literature References

1. Steketee RW, Nahlen BL, Parise ME, Menendez C. The burden of malaria in pregnancy in malaria-endemic areas. *Am J Trop Med Hyg* 2001;**64**(1-2 Suppl)**:**28-35.

2. Guyatt HL, Snow RW. The epidemiology and burden of *Plasmodium falciparum*-related anemia among pregnant women in sub-Saharan Africa. *Am. J. Trop. Med. Hyg.* 2001;**64 (Suppl):**36-44.

3. Brabin B, Piper C. Anaemia- and malaria-attributable low birthweight in two populations in Papua New Guinea. *Ann. Human Biol.* 1997;**24:**547-555.

4. Benet A, Khong TY, Ura A, et al. Placental malaria in women with South-East Asian ovalocytosis. *Am J Trop Med Hyg* 2006;**75**(4)**:**597-604.

5. Allen SJ, Raiko A, O'Donnell A, Alexander ND, Clegg JB. Causes of preterm delivery and intrauterine growth retardation in a malaria endemic region of Papua New Guinea. *Arch. Dis. Child. Fetal Neonatal Ed.* 1998;**79**(2)**:**F135-40.

6. Schultz LJ, Steketee RW, Macheso A, Kazembe P, Chitsulo L, Wirima JJ. The efficacy of antimalarial regimens containing sulfadoxine-pyrimethamine and/or chloroquine in preventing peripheral and placental *Plasmodium falciparum* infection among pregnant women in Malawi. *Am. J. Trop. Med. Hyg.* 1994;**51:**515-522.

7. Parise ME, Ayisi JG, Nahlen BL, et al. Efficacy of sufadoxine-pyrimethamine for prevention of placental malaria in an area of Kenya with a high prevalence of malaria and human immunodeficiency virus infection. *Am. J. Trop. Med. Hyg.* 1998;**59**(5)**:**813-822.

8. Verhoeff FH, Brabin BJ, Chimsuku L, Kazembe P, Russell WB, Broadhead RL. An evaluation of the effects of intermittent sulfadoxine-pyrimethamine treatment in pregnancy on parasite clearance and risk of low birthweight in rural Malawi. *Annals Trop. Med. Parasitol.* 1998;**92:**141-150.

9. Shulman CE, Dorman EK, Cutts F, et al. Intermittent sulphadoxine-pyrimethamine to prevent severe anaemia secondary to malaria in pregnancy: a randomised placebo-controlled trial. *Lancet* 1999;**353:**632-636.

10. Rogerson SJ, Chaluluka E, Kanjala M, Mkundika P, Mhango CG, Molyneux ME. Intermittent sulphadoxine-pyrimethamine in pregnancy: effectiveness against malaria morbidity in Blantyre, Malawi 1997-1999. *Trans. R. Soc. Trop. Med. Hyg.* 2000;**94:**549-553.

11. Kublin JG, Dzinjalamala FK, Kamwendo DD, et al. Molecular markers for treatment failure of sulfadoxine-pyrimethamine and chlorproguanil-dapsone for falciparum malaria and a model for practical application in Africa. *J. Infect. Dis.* 2001**:**(submitted).

12. Mueller I, Bockarie M, Alpers M, Smith T. The epidemiology of malaria in Papua New Guinea. *Trends Parasitol* 2003;**19**(6)**:**253-9.

13. Casey GJ, Ginny M, Uranoli M, et al. Molecular analysis of Plasmodium falciparum from drug-treatment failure patients in Papua New Guinea. *Am J Trop Med Hyg* 2004;**70**(3)**:**251-5.

14. ter Kuile FO, van Eijk AM, Filler SJ. Effect of sulfadoxine-pyrimethamine resistance on the efficacy of intermittent preventive therapy for malaria control during pregnancy: a systematic review. *Jama* 2007;**297**(23)**:**2603-16.

15. Kalilani L, Mofolo I, Chaponda M, et al. A randomized controlled pilot trial of azithromycin or artesunate added to sulfadoxine-pyrimethamine as treatment for malaria in pregnancy. *JAMA (submitted)*.

16. DUNNE MW, SINGH N, SHUKLA M, et al. A DOUBLE-BLIND, RANDOMIZED STUDY OF AZITHROMYCIN COMPARED TO CHLOROQUINE FOR THE TREATMENT OF PLASMODIUM VIVAX MALARIA IN INDIA. *Am J Trop Med Hyg* 2005;**73**(6)**:**1108-1111.

17. Dunne MW, Singh N, Shukla M, et al. A Multicenter Study of Azithromycin, Alone and in Combination with Chloroquine, for the Treatment of Acute Uncomplicated Plasmodium falciparum Malaria in India. *J Infect Dis* 2005;**191**(10)**:**1582-1588.

18. Mayxay M, Pukrittayakamee S, Newton PN, White NJ. Mixed-species malaria infections in humans. *Trends. Parasitol.* 2004;**20**(5)**:**233-40.

19. Perandin F, Manca N, Calderaro A, et al. Development of a Real-Time PCR Assay for Detection of Plasmodium falciparum, Plasmodium vivax, and Plasmodium ovale for Routine Clinical Diagnosis. *J. Clin. Microbiol.* 2004;**42**(3)**:**1214-1219.

20. Nosten F, McGready R, Simpson JA, et al. Effects of *Plasmodium vivax* malaria in pregnancy. *Lancet* 1999;**354:**546-9.

21. Mendis K, Sina BJ, Marchesini P, Carter R. The neglected burden of Plasmodium vivax malaria. *Am. J. Trop. Med. Hyg.* 2001;**64**(1-2 Suppl)**:**97-106.

22. Smith T, Genton B, Baea K, Gibson N, Narara A, Alpers MP. Prospective risk of morbidity in relation to malaria infection in an area of high endemicity of multiple species of *Plasmodia*. *Am. J. Trop. Med. Hyg.* 2001;**64:**262-267.

23. Mehlotra RK, Kasehagen LJ, Baisor M, et al. Malaria infections are randomly distributed in diverse holoendemic areas of Papua New Guinea. *Am. J. Trop. Med. Hyg.* 2002;**67**(6)**:**555-62.

24. Luxemburger C, Ricci F, Nosten F, Raimond D, Bathet S, White NJ. The epidemiology of severe malaria in an area of low transmission in Thailand. *Trans R Soc Trop Med Hyg* 1997;**91**(3)**:**256-62.

25. Williams TN, Maitland K, Bennett S, et al. High incidence of malaria in α-thalassaemic children. *Nature* 1996;**383:**1996.

26. Price RN, Simpson JA, Nosten F, et al. Factors contributing to anemia after uncomplicated falciparum malaria. *Am. J. Trop. Med. Hyg.* 2001;**65:**614-622.

27. Duffy PE, Fried M. Antibodies that inhibit Plasmodium falciparum adhesion to chondroitin sulfate A are associated with increased birth weight and the gestational age of newborns. *Infect. Immun.* 2003;**71**(11)**:**6620-3.

28. Staalsoe T, Shulman CE, Bulmer JN, Kawuondo K, Marsh K, Hviid L. Variant surface antigen-specific IgG and protection against clinical consequences of pregnancy-associated Plasmodium falciparum malaria. *Lancet* 2004;**363**(9405)**:**283-9.

29. Beeson JG, Mann EJ, Elliott SR, et al. Antibodies to variant surface antigens of *Plasmodium falciparum*-infected erythrocytes and adhesion inhibitory antibodies are associated with placental malaria and have overlapping and distinct targets. *J. Infect. Dis.* 2004;**189**(3)**:**540-51.

30. Rogerson SJ, Pollina E, Getachew A, Tadesse E, Lema VM, Molyneux ME. Placental monocyte infiltrates in response to *Plasmodium falciparum* infection and their association with adverse pregnancy outcomes. *Am. J. Trop. Med. Hyg.* 2003;**68:**115-119.

31. Ordi J, Ismail MR, Ventura P, et al. Massive chronic intervillositis of the placenta associated with malarial infection. *Am. J. Surg. Path.* 1998;**22:**1006-1011.

32. Menendez C, Ordi J, Ismail MR, et al. The impact of placental malaria on gestational age and birth weight. *J. Infect. Dis.* 2000;**181:**1740-5.

33. Genton B, al-Yaman F, Mgone CS, et al. Ovalocytosis and cerebral malaria. *Nature* 1995;**378**(6557)**:**564-5.

34. Allen SJ, O'Donnell A, Alexander ND, et al. Prevention of cerebral malaria in children in Papua New Guinea by southeast Asian ovalocytosis band 3. *Am. J. Trop. Med. Hyg.* 1999;**60**(6)**:**1056-60.

35. Maier AG, Duraisingh MT, Reeder JC, et al. Plasmodium falciparum erythrocyte invasion through glycophorin C and selection for Gerbich negativity in human populations. *Nat. Med.* 2003;**9**(1)**:**87-92.

# SUPPLEMENTS/APPENDICES

## Study Follow-up Schedule Diagram

|  | ≤13 wk | 14-20 wk | 22-26 wk | 28-34 wk | delivery | 6 wk pp | 3 m pp | 12 month |
| --- | --- | --- | --- | --- | --- | --- | --- | --- |
| Study contacts | • | • | • | • | • | • |  | • |
| Ultrasound scans | • | • | • | • |  |  |  |  |
| Treatment visits |  | • | • | • |  |  |  |  |
| Sample collections  Venous blood | • |  |  |  | • |  |  |  |
| Finger prick |  | • | • | • |  |  |  |  |
| Main outcome measurements |  |  |  |  | • |  |  |  |
| Immunology studies | • |  |  |  | • |  |  |  |
| 250 consecutive women (immunology studies) |  |  |  |  |  |  | • |  |

The sample schedule indicated above is for women first attending at or before 13 weeks’ gestation. When women first attend between 14--26 weeks, the initial venous blood sample and ultrasound scan will take place at this visit.

## Clinical Definitions

1. **Malaria**
   1. **Asymptomatic malaria infections**

Asymptomatic malaria infections are defined as:

- presence of malaria parasites on blood slide, PLUS
- axillary temperature < 37.5ºC AND NO history of febrile illness in the previous 48 hours
  1. **Simple malaria illness / simple malaria fevers / uncomplicated malaria**

All these definitions are used synonymously.

Simple / uncomplicated malaria is defined as:

- axillary temperature > 37.5ºC or a history of febrile illness in the previous 48 hours, PLUS
- presence of malaria parasites on blood slide
- with no signs of severe malaria
  1. **Severe Malaria**

Severe malaria is defined as:

- axillary temperature > 37.5ºC or a history of febrile illness in the previous 48 hours, PLUS
- presence of malaria parasites on blood slide
- with any of the following:
  - impaired consciousness
  - respiratory distress
  - HB < 6 g/dl

1. **Anaemia**

The following definitions are used to describe the degree of anaemia:

- Severe Anaemia: Haemoglobin (Hb) < 6 g/dl
- Moderate Anaemia: 6.0 g/dl ≤ Hb < 9 g/dl
- Mild Anaemia: 9.0 g/dl ≤ Hb < 11 g/dl

**3. Sexually transmitted infection**

- Syphilis is defined as an RPR positive (latent syphilis) or typical clinical manifestations of primary or secondary syphilis associated with positive identification of spirochaetes from lesions.

- Gonorrhoea, Trichomonas and Chlamydia are defined as a HVS that is PCR positive for evidence of any of these infections

- Genital discharge is defined as a clinical complaint of foul, offensive and/or copious discharge from the vagina. It will be classified according to whether there was confirmation by study staff, and whether a swab was collected, and what the result of PCR on that swab was.

## Informed Consent – Study Information Brochure (English)

**Intermittent Preventive Treatment (IPTp) for the Prevention of Low birth weight, Malaria and infections in pregnancy**

PNG Institute of Medical Research, University of Melbourne, Walter & Eliza Hall Institute of Medical Research, University of Barcelona, Case Western Reserve University,

**Principal Investigators: A/Prof Stephen Rogerson, Dr. Ivo Mueller & Prof. Peter Siba**

**Study Location**: Madang Province, Papua New Guinea

**WHY IS THIS STUDY BEING DONE?**

You and many other pregnant women are being asked to take part in a research study being conducted by the PNG Institute of Medical Research, with help from scientists in Australia, Spain and America to investigate a new method for the prevention of malaria and anaemia in pregnant women, and low birth weight in infants.

As you will know malaria is in important illness in your community. Pregnant women are at higher risk than other adults, and malaria can affect both the mother and the baby. One possible way to reduce the effect of malaria on mothers and their babies is to give all pregnant women regular treatment with common antimalarial drugs. These drugs are given at time of antenatal clinic visits to all women whether they are sick or healthy - a bit like a ‘vaccine’.

This type of intervention is called ‘Intermittent preventive treatment’ and in earlier studies in African countries has been found to reduce the amount of malaria illness, anaemia and low birth weight babies. The PNG Health Department and mission health services are very keen to find out if the same approach would work as well in Papua New Guinea. All the women in our study will be given a treated bed net to sleep under to prevent new malaria infections.

**WHAT WILL WE DO?**

When they enter the study, half of the women in this study will be given a dose of SP and one of chloroquine. The other half will be treated with SP, plus a drug called azithromycin. At one or two later visits to antenatal clinic, you will either receive the SP and azithromycin treatments, or identical “placebo”, tablets which look like the drug but contain only harmless sugar and salt. At the end of the study we will discover which treatment is a better option for pregnant PNG women.

The treatment with azithromycin may also help treat sexually transmitted infections that pregnant women may carry which can cause severe effects in the baby. We will look for these infections by asking you to collect a swab from inside the vagina. When you come back for another study visit, we will be asking you to take a second swab to check if you still have infection. If you have not taken azithromycin – or treatment was not successful - we will give you treatment for any infection you might have.

By learning if and how this intervention works, we can advise the Health Department and Mission Health Services on the possible introduction of this intervention into the current antenatal services.

If you decide to participate in this study, you will receive drug treatments at Antenatal Clinic on up to three occasions in pregnancy. Following that you will continue to be seen until delivery, and your child will be seen at 6 weeks and 12 months of age. Study visits will stop when

- you do not want to continue in the study and withdraw your consent
- you move away from the study area, or
- your child reaches 12 months of age.

**WHO CAN TAKE PART IN THE STUDY?**

Before you take part in the study, a Nursing Officer will determine at Antenatal Clinic if you can take part. You can take part in the study, if you are up to 26 weeks pregnant

You can not take part in the study, if

- You are already more than 6 months (26 weeks) pregnant
- You live far away from any of our study sites
- You plan to have your baby outside of Madang Province

or

- You refuse to take part in the study.

**ENROLMENT & 1^st^ TREATMENT VISIT.** If you agree to take part in the study, you will be fully enrolled in to the study at your first Antenatal Clinic (ANC) visit. A study team nurse will examine you for signs of chronic illness, and check your weight and height and blood pressure. If you are currently ill due to anaemia or other problems, you can not be enrolled into the study at this visit, but if you have recovered by the time of your next visit, you could be enrolled then.

We will offer some participating women an ultrasound scan at their first visit. This scan uses harmless radio waves to create a picture of your uterus and the baby inside it. By measuring the size of the baby we cal tell how many weeks pregnant you are and how well the baby is growing. These scans can only be done at Modilon Hospital. Some women will have up to three scans, so we can measure how well the baby grows at different times in pregnancy.

The study team also will check to see if you are infected with malaria parasites. This means that the study team will take a blood sample (about 10 mls or 2 teaspoons of blood). This blood will be sent to the IMR laboratory in Goroka to see if you have malaria parasites that cannot be seen on blood smears. The blood sample also will tell us if you have inherited traits that affect how your body responds to malaria infections. We will use some of the left over blood to perform studies of malaria immunity, to discover what factors protect pregnant women from malaria.

We will use a small part of the blood sample to examine how differences in genes of different mothers might affect the outcome of the pregnancy, including how healthy and well grown your baby is. This work will be carried out after the study is finished.

The study team also will ask you some questions about you and your pregnancy, and look in your health booklet. If you do not have one, we will provide one. The Nursing Officer will review your weight & haemoglobin value, record values in the health book and advise on treatment if necessary.

We will also ask you if you could collect a swab from your vagina so we can check if you have any bacterial infections that may harm you or your child. The IMR will test the sample in its laboratory in Goroka. If you have any infection, then you will receive free treatment at the next study visit. Should you feel uncomfortable with collecting the swab, please let our nurse know and you may not need to collect it.

Following examination enrolment, you will receive a study number and treatment group and will receive the appropriate treatment. The study team will watch you taking the first dose. You will be given further doses, with instructions to take them over the next 1-2 days. A study village reporter may visit you during these days to see if you had any problems with administering the remaining treatments

**FURTHER TREATMENT VISITS.** Following enrolment you will be seen at each Antenatal Clinic visit by the study team during the normal ANC clinics. A village reporter may contact you to remind you of the time for the next visit.

During the visit the study team will check your weight and blood pressure, check the health book, and ask a few questions. A finger prick blood sample will be taken and will be sent to the IMR laboratory in Goroka to see if you have malaria parasites that cannot be seen on blood smears.

At one or two later visits, you will receive either SP and azithromycin or placebo (inactive) tablets. This will happen at 2 further visits. As with the first treatment, the study team will watch you taking the first dose. You will be given further doses, with instructions to take them over the next 1-2 days. A study village reporter will again assist you with any problems. At this visit we will ask you to collect a vaginal swab to see if you are still carrying any sexually transmitted infection.

**DELIVERY.**

We will encourage all women to have their babies in the hospital or health centre. If you do, as well as weighing and examining your baby, we will take a blood sample (10 mls or 2 teaspoons) from you, and we will also take blood and tissue samples from the placenta, and will collect blood from the umbilical cord. If you do not deliver in the Health Centre, we will need to see you and your baby as soon as possible after delivery, and certainly in the first week. At that time we will weigh and examine the baby, and take a blood sample from you.

**LONG TERM FOLLOW-UP**.

After delivery we will arrange to see you and your baby at 6 weeks of age. At this time, your baby will get vaccines that are due, and we will weigh the baby, and take a small blood sample from the heel of the baby. If your child has any health problems, our staff and the clinic staff will assess them. We will weigh you and take a small blood sample from your finger.

We will see you and your baby one final time at 12 months after delivery, when we will weigh and examine the baby. We will take a finger prick sample (about 4 drops of blood) to check the baby for malaria and anaemia

**WHAT SHOLD YOU DO IF YOU ARE SICK**

If you are sick at the time of any clinic visit, then please tell the health centre or study staff. The study team will then assist the local health centre to diagnose and treat you.

For any other illness, please come to the clinic or to the emergency service at the hospital with your study health book. A study nurse will help with your diagnosis and treatment, and if needed she will ask one of the study or hospital doctors to see you. The nurse will examine you, and she will take a small sample of blood (approximately 200-250 μl, about 4-5 drops of blood) from your finger to test for malaria and anaemia. If you are diagnosed with malaria it will be treated by the study staff. For treatment of all other illnesses you will be treated by the health centre or hospital staff.

**HOW DID WE DECIDE WHICH DRUGS TO USE?**

SP, or Fansidar is commonly used in PNG to treat malaria. It works well in pregnancy, especially with added azithromycin. We will compare this to SP and chloroquine, which has been used in PNG for some time. Both combinations are expected to work well with use of a bed net, which we will give you if you don’t already have one.

**HOW WILL WE DECIDE WHICH DRUG YOU GET?**

When you are enrolled into the study, you will be given a study number. The nurse will then open small envelop with your number on it. In that letter is a small paper with a letter that corresponds to one of the treatments printed on it. All treatment that you receive from the study team will be from the box with that letter on it.

The nurses do not know which letter corresponds to which treatment nor which envelop contain which letter. These assignments were done at random using a computer by a person that is not involved with the study.

**WHAT ARE THE RISKS OF THE STUDY?**

We do not know of any risks or side effects related to examining or weighing you. We also do not know of any side effects of having an ultrasound scan, which most women will have. The puncture from the blood draw may cause a small amount of discomfort for a few minutes, slight bruising, irritation, or tenderness. Trained study staff will use sterile methods to prevent the finger prick puncture from becoming infected. The amount of blood drawn is very small compared to the blood you have, and does not constitute a risk to your health. We will take two blood samples from the baby’s heel (at birth and at six weeks) to check for malaria, and a finger prick at 12 months of age. Each blood sample is very small (4 to 5 drops only).

All women in this study will receive Fansidar (SP) at least once. Half will receive chloroquine once. Both these drugs are regularly used in PNG to treat people with malaria. They are generally well tolerated, but as you will probably know from experience, take these drugs may sometimes be associated with mild headaches, stomach aches, diarrhoea or tiredness. Severe side effects are very rarely observed.

The women who do not get a course of chloroquine will get azithromycin. Azithromycin has not been used much in PNG, but it has been used a lot in Africa and in the USA to treat infections in pregnant women, especially chlamydia. It appears to work well against such infections and to be well tolerated. Azithromycin is generally well tolerated. The most common side effects are diarrhea or loose stools, nausea, abdominal pain, and vomiting, each of which may occur in fewer than one in twenty persons who receive azithromycin. Rarer side effects include abnormal liver tests, allergic reactions, and nervousness.

Based on earlier studies in Africa we do not expect that participation in this study carries any long-term health risk to you.

We cannot predict all risks or potential side effects. If you feel that your health has been negatively affected by the study, then do not hesitate to contact the study physician or any other study team member. We will then examine you to determine the cause of its health problems.

For more information about risks and side effects, ask the researcher or contact the local study coordinator at the IMR in Madang.

**ARE THERE BENEFITS TO TAKING PART IN THE STUDY?**

The drugs you receive will decrease the risk of malaria, which ever group you are in. We do not know if one will have more effect than the other. The azithromycin will also treat infections in the vagina you may not know you are carrying, and which could harm mothers or their babies. If you don’t get treated with azithromycin, we will test you for these infections, and give treatment if the swab shows infection.

During the time that you take part in the study, you will have access to study nursing officers and doctors, if you fall ill and present at your health centre or at Antenatal Clinics at Modilon Hospital. If you are found to be sick with malaria, you will be treated at the study’s expense.

If the intervention works well, it is likely that the Department of Health may introduce the IPTp are part of national health guidelines. In addition the study will help our understanding of malaria in pregnancy, how the growth of your baby is affected by malaria infections, and how pregnant women acquire immunity to malaria.

**WHAT ABOUT THE LABORATORY TESTS?**

Malaria parasites are very small and thus sometimes difficult to see on blood slide using a microscopy. In particular, as there are 4 different types of malaria parasites found in PNG. They can occur together in a single blood sample and are very difficult identify correctly by microscopy only. In order correctly detect all parasites in the blood collected from you, we will use special, very sensitive laboratory methods. We will thus make DNA from all blood samples collected from you and examine them for the presence of different types of malaria parasites. In addition we check for inherited traits that protect against malaria infection.

The blood collected from you (or you baby) will be used to the test how well your body has learned to defend itself against malaria infections and to look for genes that might play a part on determining easy your pregnancy was and how healthy your baby is.

Swabs from the vagina will be tested for infections. If we find these infections, we will give you counselling and treat you (and you partner) with a course of antibiotics to eradicate them.

Some women will be asked to give permission to collect a sample from your nose that will be used to see whether they are carrying certain bacteria called pneumococci that cause pneumonia in young children. We will use these samples to check if treatment with azithromycin does also have an effect on these bacteria.

These laboratory tests will be conducted at the PNG IMR laboratories in Madang and Goroka. Only a few specialised tests will be conducted in laboratories overseas. The results of these tests will not be given to you on an individual basis.

Your blood samples and DNA, and DNA from a sample of your baby’s blood collected from the umbilical cord, will be stored for research purposes. If you agree, these samples may be used as for future studies on malaria and anaemia or in an international study investigating genes that influence pregnancy outcomes in general. Please indicate your agreement by ticking the box on the signature form.

**WHAT ARE THE STUDY COSTS?**

You will not receive payment for taking part in this study.

This study will pay for all measurements, blood samples, blood smears, and laboratory tests we make, including future testing. If you fall sick with malaria the study team will treat you for free. In case of any other illness during a study team visit, you will be provided transportation to the local health centre or hospital where testing and medical care are available. Transportation will be provided to you free of charge. If you come for treatment to the health centres or to Modilon Hospital the study will cover the outpatient fees (for participating women and their newborn babies only).

**WHAT ARE MY RIGHTS AS A PARTICIPANT?**

Your participation in this study is voluntary. You may choose not to take part in this study. In this case, you will receive all the usual Antenatal Care from your clinic, as normal.

If you decide to take part in the study now, but change your mind, you may withdraw from the study at any time. If you want to stop participating, please tell a study team member. Refusing to take part or leaving the study will not result in any penalty or loss of benefits to which you or your baby are entitled.

If we learn of new information that may affect your health and well-being, risks or benefits associated with this study, or your willingness to stay in this study, we will have a study team member contact you. You can decide whether to continue to \take part in the study.

If you have a physical injury or an illness as a result of taking part in this study, you will be examined by the study physician. In such cases medical care is available at the health centres or Madang (Modilon) Hospital The PNGIMR will cover all cost for treatment of study related illness or injury. Compensation will only be paid for costs incurred for transportation and lost wages.

**WHAT ABOUT CONFIDENTIALITY?**

We will work hard to keep your personal information confidential. The results of your study visits, laboratory tests, and health centre visits will be collected and entered into computer files at the PNGIMR. After blood samples are collected, they will be identified by a bleed code and serial number and not by your name.

This information you give us will not be shared with other people. We however cannot guarantee absolute confidentiality. Your personal information may be disclosed if required by law. Organizations that may inspect and/or copy your study records or your interview information for quality assurance and data analysis include the PNG Ministry of Health, the PNGIMR, and representatives of the Data and Safety Monitoring or the PNG Medical Research Advisory Council. Data on drug safety (i.e. whether you have any side effects that might be due to the drugs you receive) will also be shared with the Malaria in Pregnancy Consortium’s Safety Working Group, based in the UK (who are collecting this data from several countries) and an independent safety panel. These groups will review the safety data from in order to ensure the better monitoring of the products involved on this research. They will not be given information that identifies you.

**WHOM DO I CALL IF I HAVE QUESTIONS OR PROBLEMS?**

If you have questions about the study or if you think you have a study-related injury, you can talk to any study team member, the PNG IMR site manager in Madang (Mr John Taime) or directly with Dr. Ivo Mueller. He can be telephoned at 732 2800 or 852 2909. For questions about your rights as a study participant, contact Mr Willie Pomat at the IMR IRB (the group of people who review the study to protect your rights) at 732-1469

## Informed Consent – Signature Form (English)

**Intermittent Preventive Treatment (IPTp) for the Prevention of Malaria and Anaemia in Pregnancy**

**PNG Institute of Medical Research, Goroka, University of Melbourne, Australia, The Walter & Eliza Hall Institute of Medical Research, Melbourne, University of Barcelona, Spain and Case Western Reserve University, Cleveland, USA**

**Principal Investigators: A/Prof Stephen Rogerson, Dr. Ivo Mueller & Prof. Peter Siba**

**Study Location: Madang Province Papua New Guinea**

**SIGNATURE FORM**

Signing below indicates that you have been informed about the research study in which you voluntarily agree to participate; that you have received the study information brochure and you have asked any questions about the study that you may have; and that the information given to you has permitted you to make a fully informed and free decision about your participation in the study.

By signing this consent form, you do not waive any legal rights, and the investigator(s) or sponsor(s) are not relieved of any liability they may have. A copy of this consent form will be provided to you.

If you do not know how to write, then please indicate your consent to your participation by making a thumb / finger mark on the signature line. An independent witness to the consent process will then be asked to also sign this document.

_______________________________________ Date________________

Printed Name of Participant

________________________________________

Participant signature or mark

If you do not know how to write, then please indicate your consent to your participation by making mark on the signature line. An independent witness to the consent process will then be asked to also sign this document. Without an independent witness signature the participant’s mark is insufficient proof for consent.

_________________________________________ Date________________

Printed Name of Witness

___________________________________________

Signature of Witness

Samples obtained from you during this study might be suitable to use in other research studies of malaria and anaemia. If you would like to authorise us to use your specimens then please indicate this be ticking the box below and write your initials on the line next to it

__________

This consent was obtained by:

_________________________________________ Date________________

Printed Name of Person Obtaining Consent

___________________________________________

Signature of Person Obtaining Consent

(Must be an individual who has been designated in the Checklist to obtain consent.)

_________________________________________ Date________________

Signature of Study Co-ordinator (Affirming subject

eligibility for the study and that informed consent has been obtained.)

## Preliminary Study: Informed Consent – Study Information Brochure (English)

## Preliminary Study: Informed Consent – Signature Form (English)

**Preliminary Study on Prevalence of Low birth weight, Malaria and Infections in Pregnancy**

**PNG Institute of Medical Research, Goroka, University of Melbourne, Australia, The Walter & Eliza Hall Institute of Medical Research, Melbourne, University of Barcelona, Spain and Case Western Reserve University, Cleveland, USA**

**Principal Investigators: A/Prof Stephen Rogerson, Dr. Ivo Mueller & Prof. Peter Siba**

**Study Location: Madang Province Papua New Guinea**

**SIGNATURE FORM**

Signing below indicates that you have been informed about the research study in which you voluntarily agree to participate; that you have received the study information brochure and you have asked any questions about the study that you may have; and that the information given to you has permitted you to make a fully informed and free decision about your participation in the study.

By signing this consent form, you do not waive any legal rights, and the investigator(s) or sponsor(s) are not relieved of any liability they may have. A copy of this consent form will be provided to you.

If you do not know how to write, then please indicate your consent to your participation by making a thumb / finger mark on the signature line. An independent witness to the consent process will then be asked to also sign this document.

_______________________________________ Date________________

Printed Name of Participant

________________________________________

Participant signature or mark

If you do not know how to write, then please indicate your consent to your participation by making mark on the signature line. An independent witness to the consent process will then be asked to also sign this document. Without an independent witness signature the participant’s mark is insufficient proof for consent.

_________________________________________ Date________________

Printed Name of Witness

___________________________________________

Signature of Witness

Samples obtained from you during this study might be suitable to use in other research studies of malaria and anaemia. If you would like to authorise us to use your specimens then please indicate this be ticking the box below and write your initials on the line next to it

__________

This consent was obtained by:

_________________________________________ Date________________

Printed Name of Person Obtaining Consent

___________________________________________

Signature of Person Obtaining Consent

(Must be an individual who has been designated in the Checklist to obtain consent.)

_________________________________________ Date________________

Signature of Study Co-ordinator (Affirming subject

eligibility for the study and that informed consent has been obtained.)

**Study of Pregnancy and Immunity to Vivax Malaria**

**PNG Institute of Medical Research, Goroka, University of Melbourne, Australia, University of Barcelona, Spain**

**Principal Investigators: A/Prof Stephen Rogerson, Dr. Ivo Mueller & Prof. Peter Siba**

**Study Location: Madang Province Papua New Guinea**

**SIGNATURE FORM**

Signing below indicates that you have been informed about the research study in which you voluntarily agree to participate; that you have received the study information brochure and you have asked any questions about the study that you may have; and that the information given to you has permitted you to make a fully informed and free decision about your participation in the study.

By signing this consent form, you do not waive any legal rights, and the investigator(s) or sponsor(s) are not relieved of any liability they may have. A copy of this consent form will be provided to you.

If you do not know how to write, then please indicate your consent to your participation by making a thumb / finger mark on the signature line. An independent witness to the consent process will then be asked to also sign this document.

_______________________________________ Date________________

Printed Name of Participant

________________________________________

Participant signature or mark

If you do not know how to write, then please indicate your consent to your participation by making mark on the signature line. An independent witness to the consent process will then be asked to also sign this document. Without an independent witness signature the participant’s mark is insufficient proof for consent.

_________________________________________ Date________________

Printed Name of Witness

___________________________________________

Signature of Witness

Samples obtained from you during this study might be suitable to use in other research studies of malaria and anaemia. If you would like to authorise us to use your specimens then please indicate this be ticking the box below and write your initials on the line next to it

__________

This consent was obtained by:

_________________________________________ Date________________

Printed Name of Person Obtaining Consent

___________________________________________

Signature of Person Obtaining Consent

(Must be an individual who has been designated in the Checklist to obtain consent.)

_________________________________________ Date________________

Signature of Study Co-ordinator (Affirming subject

eligibility for the study and that informed consent has been obtained.)

**Study of Pregnancy and Immunity to Vivax Malaria**

PNG Institute of Medical Research, University of Melbourne, University of Barcelona

**Principal Investigators: A/Prof Stephen Rogerson, Dr. Ivo Mueller & Prof. Peter Siba, Prof Clara Menendez**

**Study Location**: Madang Province, Papua New Guinea

**WHY IS THIS STUDY BEING DONE?**

You have been participating in a study of antimalarial drug treatment in pregnancy. Linked to this study we want to investigate how pregnancy affects a woman’s immune response to malaria, especially to vivax malaria. We want to do this by comparing responses in women *while*  they are pregnant, compared to the same women’s responses  *after*  their pregnancy.

**WHO CAN TAKE PART IN THE STUDY?**

You can take part in the study, if you have participated in the study of malaria in pregnancy.

**ADDITIONAL FOLLOW UP VISIT.** If you agree to take part in the study, you will be asked to come for an additional visit to the clinic. This visit will take place at the time your child is due for their 3 month vaccinations. At that visit the study nurse will take 10 ml, or 2 teaspoons, of blood from you, to test for malaria and anaemia, and to see whether your white blood cells respond differently to malaria now you are no longer pregnant. We will also give you and your baby a health check.

**WHAT ARE THE RISKS OF THE STUDY?**

The puncture from the blood draw may cause a small amount of discomfort for a few minutes, slight bruising, irritation, or tenderness. The amount of blood drawn is very small compared to the blood you have, and does not constitute a risk to your health.

**ARE THERE BENEFITS TO TAKING PART IN THE STUDY?**

During the time that you take part in the study, you will have access to study nursing officers and doctors, if you fall ill and present at your health centre or at Antenatal Clinics at Modilon Hospital. If you are found to be sick with malaria, you will be treated at the study’s expense.

**WHAT ABOUT THE LABORATORY TESTS?**

The blood collected from you at this extra visit will be used to the test how well your body has learned to defend itself against malaria infections. These laboratory tests will be conducted at the PNG IMR laboratories in Madang and Goroka. Only a few specialised tests will be conducted in laboratories overseas. The results of these tests will not be given to you on an individual basis.

**WHAT ARE THE STUDY COSTS?**

You will not receive payment for taking part in this study.

**WHAT ARE MY RIGHTS AS A PARTICIPANT?**

Your participation in this study is voluntary. You may choose not to take part in this study. If you decide to take part in the study now, but change your mind, you may withdraw from the study at any time. If you want to stop participating, please tell a study team member. Refusing to take part or leaving the study will not result in any penalty or loss of benefits to which you or your baby are entitled.

**WHAT ABOUT CONFIDENTIALITY?**

We will work hard to keep your personal information confidential. The results of your laboratory tests will be collected and entered into computer files at the PNGIMR. After blood samples are collected, they will be identified by a bleed code and serial number and not by your name. This information you give us will not be shared with other people.

**WHOM DO I CALL IF I HAVE QUESTIONS OR PROBLEMS?**

If you have questions about the study or if you think you have a study-related injury, you can talk to any study team member, the PNG IMR site manager in Madang (Mr John Taime) or directly with Dr. Ivo Mueller. He can be telephoned at 732 2800 or 852 2909. For questions about your rights as a study participant, contact Mr Willie Pomat at the IMR IRB (the group of people who review the study to protect your rights) at 732-146
