# Supplementary figures and images for: Sulphadoxine-pyrimethamine plus azithromycin for the prevention of low birthweight in Papua New Guinea: a randomised controlled trial
Source: BMC Med. 2015 Jan 16;13:9. doi: 10.1186/s12916-014-0258-3 (PMC4305224; doi:10.1186/s12916-014-0258-3)

# MADANG DISTRICT HEALTH FACILITIES

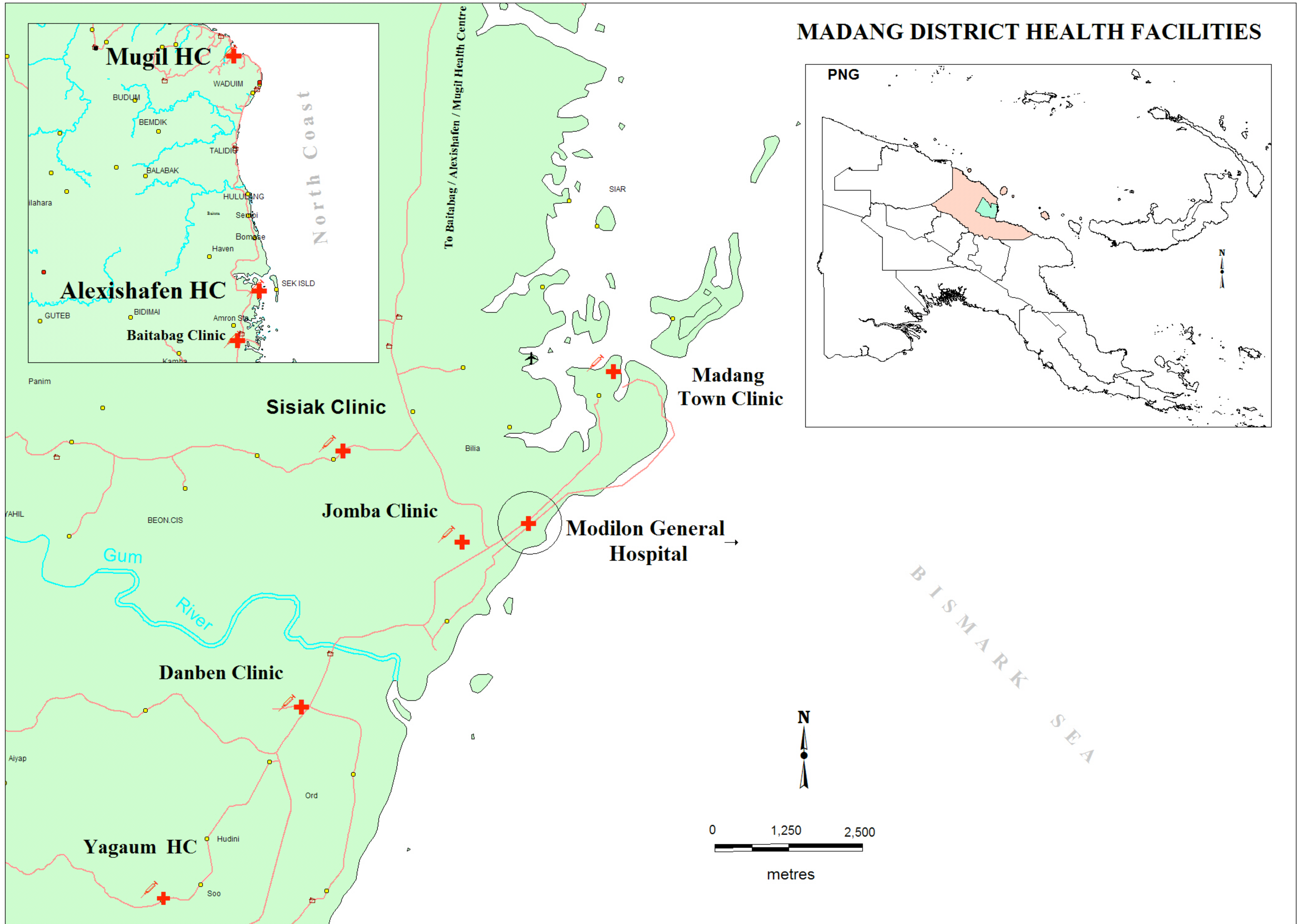

Supplement: Additional file 3: — Map of study area including location of recruitment antenatal clinics. [file 12916_2014_258_MOESM3_ESM.pdf]
