# Supplementary material for: Sulphadoxine-pyrimethamine plus azithromycin for the prevention of low birthweight in Papua New Guinea: a randomised controlled trial
Source: BMC Med. 2015 Jan 16;13:9. doi: 10.1186/s12916-014-0258-3 (PMC4305224; doi:10.1186/s12916-014-0258-3)
Supplement: Additional file 4: Table S1. — Comparison of baseline characteristics of study participants included in, and excluded from, the intention-to-treat (ITT) birthweight analysis. Table S2. Comparison of baseline characteristics of study participants excluded from the ITT birthweight analysis, by treatment arm (n = 754). Table S3. Factors associated with low birthweight on crude analysis. Table S4. Stratified analysis of low birthweight for gravidity, maternal height, maternal ethnic parentage, and bed net use before enrolment, by treatment group. [file 12916_2014_258_MOESM4_ESM.doc]

SUPPLEMENTARY TABLE 1

Comparison of baseline characteristics of study participants included in, and excluded from, the ITT birthweight analysis

| Characteristic | | Included  (n = 2,021) | | | Excluded  (n = 754) | | | *P* |
| --- | --- | --- | --- | --- | --- | --- | --- | --- |
|  | | Total |  |  | Total |  |  |  |
| Age, years | | 2,021 | 24 | (20-28) | 754 | 23 | (20-27) | 0.121 |
| Height, cm | | 1,988 | 154.3 | [5.8] | 741 | 153.9 | [6.0] | 0.173 |
| Body mass index, kg/m^2^ | | 1,983 | 22.5 | (20.9-24.3) | 738 | 22.4 | (20.7-24.3) | 0.308 |
| Mid-upper arm circumference, cm | | 1,978 | 24 | (22-25) | 736 | 24 | (22-25) | 0.656 |
| Haemoglobin (Hb), g/dL | | 1,935 | 9.7 | [1.5] | 715 | 9.7 | [1.6] | 0.711 |
| Fundal height, cm | | 2,018 | 22 | (19-24) | 753 | 22 | (18-24) | **0.004** |
| Anaemia (Hb < 11 g/dL) | | 1,935 | 1,587 | (82.0) | 715 | 570 | (79.7) | 0.178 |
| Syphilis | | 1,669 | 27 | (1.6) | 619 | 8 | (1.3) | 0.365 |
| No. of previous pregnancies | | 2,018 |  |  | 752 |  |  |  |
|  | 0 |  | 1,000 | (49.6) |  | 390 | (51.9) |  |
|  | 1 |  | 419 | (20.8) |  | 152 | (20.2) |  |
|  | ≥2 |  | 599 | (29.7) |  | 210 | (27.9) | 0.538 |
| Previous adverse pregnancy outcome | | 2,013 | 155 | (7.7) | 751 | 67 | (8.9) | 0.293 |
| Used bed net before enrolment (2 wks) | | 2,013 |  |  | 752 |  |  |  |
|  | Not used |  | 466 | (23.2) |  | 203 | (27.0) |  |
|  | Used, without insecticide |  | 842 | (41.8) |  | 307 | (40.8) |  |
|  | Used, insecticide-treated |  | 705 | (35.0) |  | 242 | (32.2) | 0.092 |
| Used antimalarials in this pregnancy | | 1,971 | 224 | (11.4) | 735 | 83 | (11.3) | 0.958 |
| Malaria infection (light microscopy) | | 2,015 | 135 | (6.7) | 751 | 69 | (9.2) | **0.026** |
| Smoker | | 2,019 | 390 | (19.3) | 753 | 134 | (17.8) | 0.363 |
| Residence | | 2,013 |  |  | 751 |  |  |  |
|  | Urban |  | 316 | (15.7) |  | 95 | (12.6) |  |
|  | Peri-urban |  | 366 | (18.2) |  | 112 | (14.9) |  |
|  | Rural |  | 1,227 | (61.0) |  | 493 | (65.6) |  |
|  | Migrant^a^ |  | 104 | (5.2) |  | 51 | (6.8) | **0.010** |
| Literate | | 2,016 | 1,826 | (90.6) | 751 | 645 | (85.9) | **<0.001** |
| Income-generating activity (woman) | | 1,919 | 1,074 | (56.0) | 711 | 383 | (53.9) | 0.336 |
| Income-generating activity (father) | | 1,994 | 1,380 | (69.2) | 742 | 506 | (68.2) | 0.611 |
| Highlander parentage | | 2,019 | 120 | (5.9) | 754 | 57 | (7.6) | 0.121 |
| Assigned to SPCQ | | 2,021 | 1,008 | (49.9) | 754 | 374 | (49.6) | 0.898 |

**Note.** Data are no. of women with outcome (%), median (IQR) or mean [standard deviation]. *P* < 0.05 marked in bold.

^a^ Women had been staying at designated residence < 2 months prior to enrolment

SUPPLEMENTARY TABLE 2

Comparison of baseline characteristics of study participants excluded from the ITT birthweight analysis, by treatment arm (n = 754)

| Characteristic | | SPCQ (n = 374) | |  | SPAZ (n = 380) | | *P* |
| --- | --- | --- | --- | --- | --- | --- | --- |
|  | | Total |  |  | Total |  |  |
| Age, years | | 374 | 23 (20-27) |  | 380 | 23 (20-28) | 0.714 |
| Height, cm | | 369 | 153.7 [5.9] |  | 372 | 154.2 [6.2] | 0.200 |
| Body mass index, kg/m2 | | 368 | 22.5 (20.8-24.1) |  | 370 | 22.3 (20.6, 24.5) | 0.664 |
| Mid-upper arm circumference, cm | | 367 | 24 (22-25) |  | 369 | 24 (22-25) | 0.198 |
| Haemoglobin (Hb), g/dL | | 350 | 9.7 [1.5] |  | 365 | 9.7 [1.6] | 0.956 |
| Fundal height, cm | | 374 | 22 (19-24) |  | 379 | 21 (18-24) | 0.198 |
| Anaemia (Hb < 11 g/dL) | | 350 | 278 (79.4) |  | 365 | 292 (80.0) | 0.849 |
| Syphilis | | 303 | 3 (1.0) |  | 316 | 5 (1.6) | 0.386 |
| No. of previous pregnancies | | 373 |  |  | 379 |  |  |
|  | 0 |  | 195 (52.3) |  |  | 195 (51.5) |  |
|  | 1 |  | 75 (20.1) |  |  | 77 (20.3) |  |
|  | ≥2 |  | 103 (27.6) |  |  | 107 (28.2) | 0.973 |
| Previous adverse pregnancy outcome | | 372 | 31 (8.3) |  | 379 | 36 (9.5) | 0.575 |
| Used bed net before enrolment (2 wks) | | 374 |  |  | 378 |  |  |
|  | Not used |  | 98 (26.2) |  |  | 105 (27.8) |  |
|  | Used, without insecticide |  | 158 (42.3) |  |  | 149 (39.4) |  |
|  | Used, insecticide-treated |  | 118 (31.6) |  |  | 124 (32.8) | 0.729 |
| Used antimalarials in this pregnancy | | 364 | 44 (12.1) |  | 371 | 39 (10.5) | 0.500 |
| Malaria infection (light microscopy) | | 372 | 32 (8.6) |  | 379 | 37 (9.8) | 0.582 |
| Smoker | | 374 | 65 (17.4) |  | 379 | 69 (18.2) | 0.767 |
| Residence | | 372 |  |  | 379 |  |  |
|  | Urban |  | 49 (13.2) |  |  | 46 (12.1) |  |
|  | Peri-urban |  | 52 (14.0) |  |  | 60 (15.8) |  |
|  | Rural |  | 243 (65.3) |  |  | 250 (66.0) |  |
|  | Migrant^a^ |  | 28 (7.5) |  |  | 23 (6.1) | 0.755 |
| Literate | | 373 | 318 (85.3) |  | 327 | 327 (86.5) | 0.622 |
| Income-generating activity (woman) | | 351 | 191 (54.4) |  | 360 | 192 (53.3) | 0.772 |
| Income-generating activity (father) | | 367 | 243 (66.2) |  | 375 | 263 (70.1) | 0.252 |
| Maternal highlander parentage | | 374 | 32 (8.6) |  | 380 | 25 (6.6) | 0.304 |

**Note.** Data are no. of women with outcome (%), median (IQR) or mean [standard deviation]. *P* < 0.05 marked in bold.

^a^ Women had been staying at designated residence < 2 months prior to enrolment

SUPPLEMENTARY TABLE 3

Factors associated with low birthweight on crude analysis

| **Characteristic** | |  | | | |
| --- | --- | --- | --- | --- | --- |
|  | | **% LBW** | **(n with LBW/**  **total)** | **Risk ratio**  **(95% CI)** | ***P*** |
| **Infant gender** | |  |  |  |  |
|  | Male | 12.2 | (108/889) | 1.00 |  |
|  | Female | 17.5 | (196/1,122) | 1.19 (1.08, 1.31) | **<0.001** |
| **Gravidity** | |  |  |  |  |
|  | Primigravida | 21.5 | (215/1,000) | 1.00 |  |
|  | Secundigravida | 10.7 | (45/419) | 0.50 (0.37, 0.68) |  |
|  | Multigravida (≥ 3) | 7.4 | (44/599) | 0.34 (0.25, 0.47) | **<0.001** |
| **Recruitment location** | |  |  |  |  |
|  | Modilon & Town | 16.3 | (92/563) | 1.00 |  |
|  | Danben & Yagaum | 19.0 | (85/448) | 1.16 (0.89, 1.52) |  |
|  | Alexishafen & Baitabag | 14.3 | (62/433) | 0.88 (0.65, 1.18) |  |
|  | Jomba & Sisiak | 11.9 | (52/436) | 0.73 (0.53, 1.00) |  |
|  | Mugil | 9.9 | (14/141) | 0.61 (0.36, 1.03) | **0.014** |
| **Bed net use during pregnancy^a^** | |  |  |  |  |
|  | Regular | 13.8 | (198/1,432) | 1.00 |  |
|  | Intermittent | 18.0 | (105/583) | 1.33 (1.08, 1.66) | **0.017** |
| **Maternal mid-upper arm circumference, cm** | |  |  |  |  |
|  | ≥ 23 | 13.1 | (193/1,469) | 1.00 |  |
|  | < 23 | 20.3 | (112/552) | 1.54 (1.25, 1.90) | **<0.001** |
| **Maternal height, cm** | |  |  |  |  |
|  | ≥ 150 | 13.6 | (225/1,650) | 1.00 |  |
|  | < 150 | 21.6 | (80/371) | 1.58 (1.26, 1.99) | **<0.001** |
| **Highlander parentage (woman)** | |  |  |  |  |
|  | No | 15.6 | (297/1,901) | 1.00 |  |
|  | Yes | 6.8 | (8/120) | 0.43 (0.22, 0.84) | **0.006** |
| **Income-generating activity (partner)** | |  |  |  |  |
|  | No | 18.4 | (113/614) | 1.00 |  |
|  | Yes | 13.1 | (181/1,380) | 0.71 (0.58, 0.88) | **0.002** |
| **Number of IPTp study visits** | |  |  |  |  |
|  | ≤ 1 | 26.0 | (20/77) | 1.00 |  |
|  | 2 | 18.2 | (54/296) | 0.70 (0.45, 1.10) |  |
|  | ≥ 3^b^ | 14.0 | (231/1,648) | 0.54 (0.36, 0.80) | **0.004** |
| **Time difference between birth and infant weight measurement** | |  |  |  |  |
|  | < 24 hours postpartum | 14.0 | (251/1,791) | 1.00 |  |
|  | ≥ 24 & < 48 hours | 26.5 | (35/132) | 1.89 (1.39, 2.57) |  |
|  | ≥ 48 & < 72 hours | 26.5 | (13/49) | 1.89 (1.17, 3.06) |  |
|  | ≥ 72 & < 7 days | 12.3 | (6/49) | 0.88 (0.41, 1.87) | **<0.001** |

**Note.** LBW, low birthweight; CI, confidence interval. *P* < 0.05 marked in bold.

^a^ Composite variable based on self-reported bed net use during previous 2 weeks (insecticide-treated and untreated nets combined) as assessed at recruitment and study visits; regular: usage reported each time; intermittent: mother reported not using net at ≥1 visit – includes non-users (n = 9)

^b^ Four women had 4 treatment courses and birthweight follow-up

SUPPLEMENTARY TABLE 4

Stratified analysis of low birthweight for gravidity, maternal height, maternal ethnic parentage and bed net use before enrolment, by treatment group

| Characteristic | |  |  |  |  |  |  |  |
| --- | --- | --- | --- | --- | --- | --- | --- | --- |
|  | | Control  (SPCQ & placebo) | Intervention (SPAZ) | Absolute risk difference  (95% CI) | Risk ratio  (95% CI) | *P* | Adjusted risk ratio  (95% CI) | *P* |
| Gravidity | |  |  |  |  |  |  |  |
|  | Primigravida | 123/486 (25.3) | 92/514 (17.9) | 7.4 (2.3, 12.5) | 0.71 (0.56, 0.90) | 0.004 | 0.71 (0.56, 0.89) | **0.004** |
|  | Multigravida (≥ 2) | 51/520 (9.8) | 38/498 (7.6) | 2.2 (1.3, 5.6) | 0.79 (0.45, 1.37) | 0.395 | 0.77 (0.52, 1.13) | 0.182 |
| Maternal height | |  |  |  |  |  |  |  |
|  | < 150 cms | 45/177 (25.4) | 35/194 (18.0) | 7.4 (1.0, 15.8) | 0.71 (0.48, 1.05) | 0.084 | 0.71 (0.49, 1.05) | 0.088 |
|  | ≥ 150 cm | 130/831 (15.6) | 95/819 (11.6) | 4.0 (0.7, 7.4) | 0.74 (0.58, 0.95) | 0.017 | 0.74 (0.58, 0.94) | **0.013** |
| Maternal ethnic parentage | |  |  |  |  |  |  |  |
|  | Highlander | 7/67 (10.5) | 1/53 (1.9) | 8.6 (0.4, 16.8) | 0.18 (0.02, 1.42) | 0.076 | 0.22 (0.03, 1.63) | 0.138 |
|  | Other | 168/941 (17.9) | 129/960 (13.4) | 4.4 (1.2, 7.7) | 0.75 (0.61, 0.93) | 0.008 | 0.74 (0.60, 0.91) | **0.004** |
| Used bed net before enrolment (2 wks) | |  |  |  |  |  |  |  |
|  | Not used | 40/224 (17.9) | 40/242 (16.5) | 1.3 (-5.5, 8.2) | 0.93 (0.62, 1.38) | 0.704 | 0.92 (0.62, 1.34) | 0.652 |
|  | Used | 134/781 (17.2) | 89/769 (11.6) | 5.6 (2.1, 9.1) | 0.68 (0.53, 0.87) | 0.002 | 0.67 (0.52, 0.85) | **0.001** |

**Note.** Ajdusted risk ratios were obtained after adjusting for infant gender, gravidity, number of study visits, study location, bed net use, nutritional status, season of delivery, maternal height, maternal highlander heritage and time difference between birth and weight measurement. *P* < 0.05 marked in bold.
